# Polymer Conductive Membrane-Based Circular Capacitive Pressure Sensors from Non-Touch Mode of Operation to Touch Mode of Operation: An Analytical Solution-Based Method for Design and Numerical Calibration

**DOI:** 10.3390/polym14183850

**Published:** 2022-09-14

**Authors:** Qi Zhang, Fei-Yan Li, Xue Li, Xiao-Ting He, Jun-Yi Sun

**Affiliations:** 1 School of Civil Engineering, Chongqing University, Chongqing 400045, China; 2 Key Laboratory of New Technology for Construction of Cities in Mountain Area (Chongqing University), Ministry of Education, Chongqing 400045, China

**Keywords:** capacitive pressure sensor, polymer conductive membrane, non-touch and touch mode of operation, analytical solution, numerical calibration

## Abstract

Polymer-based conductive membranes play an important role in the development of elastic deflection-based pressure sensors. In this paper, an analytical solution-based method is presented for the design and numerical calibration of polymer conductive membrane-based circular capacitive pressure sensors from non-touch mode of operation to touch mode of operation. The contact problem of a circular membrane in frictionless contact with a rigid flat plate under pressure is analytically solved, and its analytical solution is used for the design of touch mode circular capacitive pressure sensors for the first time. The analytical relationship with input pressure as independent variable and output capacitance as dependent variable is precisely derived and is used for the numerical calibrations of the analytical relationships with input capacitance as the independent variable and output pressure as the dependent variable in order to meet the capacitive pressure sensor mechanism of detecting pressure by measuring capacitance. For the first time, an example showing the design and numerical calibration of a given (given design parameters) polymer conductive membrane-based circular capacitive pressure sensor from non-touch mode of operation to touch mode of operation is provided. Then, the influence of changing several important design parameters on input capacitance–output pressure relationships is comprehensively investigated in order to clarify the desired input–output relationships when changing design parameters.

## 1. Introduction

Membrane structures, which are often characterized by large flexibility and thus endure large deformation under external loads [1,2,3,4,5,6], are widely applied to many engineering and technical fields [7,8,9]. In particular, the flexibility of membranes leads to the possibility of the design and development of elastic deflection-based devices [10,11,12,13,14,15,16,17]. Among these devices, capacitive pressure sensors are a representative example of physical quantity detection (pressure) by deflection measurement. Capacitive pressure sensors have many advantages, including high sensitivity, stability, reliability, performance-to-price ratio, low power consumption, sensitivity to side stress, no turn-on temperature drift, and other environment effects. Small volume capacitive pressure sensors used in microelectromechanical systems (MEMS) are usually used in parallel (multiple sensors) [18,19,20], and the materials used are usually silicon, silicon carbide thin films [21,22,23], polymer/ceramic thin films [24], graphene-polymer heterostructure thin films [25,26,27,28], or low-temperature co-fired ceramic thin films [29].

A membrane elastic deflection-based capacitive pressure sensor is mainly composed of a movable electrode plate (an initially flat conductive elastic membrane) and a fixed electrode plate on a substrate. On application of pressure, the initially flat conductive membrane freely deflects towards the fixed electrode plate. When the pressure is high enough, the conductive membrane touches the insulator layer coating on the fixed electrode plate. Before the conductive membrane touches the insulator layer, the capacitive pressure sensor is said to be in non-touch mode of operation [30,31,32,33,34,35], and after the conductive membrane touches the insulator layer, it is said to be in touch mode of operation [28,36,37,38,39,40]. Obviously, a change in pressure gives rise to a change in membrane deflection, which in turn causes a change in the capacitance between the conductive membrane and the insulator layer. Thus, the applied pressure can be expected to be determined by measuring the change in capacitance if the analytical relationship between pressure and deflection can be obtained, which is the basic principle of such capacitive pressure sensors.

However, the analytical relationship between the applied pressure and the deflection of the conductive membrane is often difficult to establish precisely due to the following two aspects of the difficulties. For non-touch mode capacitive pressure sensors, large pressure measurement ranges require the conductive membrane under pressure to produce larger elastic deflections, leading to difficulties in analytical solutions due to the large rotation angle of the membrane. For touch mode capacitive pressure sensors, in addition to the difficulty due to the large rotation angle of the membrane, the large deflection problem of a membrane in contact with a rigid flat plate has always been difficult to solve analytically. In fact, non-touch mode capacitive pressure sensors have long been considered inferior to touch mode capacitive pressure sensors in terms of ease of achieving nearly linear input pressure–output capacitance relationships [36]. Therefore, in recent years, it has become difficult to find research reports on non-touch mode capacitance pressure sensors in the existing literature, while a large number of research reports have dealt with touch mode capacitive pressure sensors [38,41,42,43,44,45,46].

However, our recent study [47] shows that nearly linear input–output relationships with input capacitance as independent variable and output pressure as the dependent variable can be easily achieved with the proposed analytical solution-based numerical calibration method for non-touch mode circular capacitive pressure sensors. Thus, the view in the literature, that is, that with non-touch mode capacitive pressure sensors it is not easy to achieve nearly linear input–output relationships, is open to debate. It should be pointed out that the capacitive pressure sensor mechanism of detecting pressure by measuring capacitance requires the input capacitance–output pressure relationships with capacitance to be the independent variable and pressure to be the dependent variable; thus, input pressure–output capacitance relationships with pressure as the independent variable and capacitance as the dependent variable are not applicable. However, due to the lack of accurate analytical solutions and their effective applications, input capacitance–output pressure relationships with capacitance as independent variable and pressure as dependent variable have not been provided in previous studies, whether for non-touch mode capacitive pressure sensors [30,31,32,33,34,35] or for touch mode capacitive pressure sensors [36,37,38,39,40,41,42,43,44,45,46]. This is why previous studies have often focused on deriving nearly linear input pressure–output capacitance relationships.

In this paper, the analytical solution of a circular membrane in frictionless contact with a rigid flat plate under pressure is precisely derived and used for the design of touch mode circular capacitive pressure sensors for the first time. The analytical relationship with input pressure as the independent variable and output capacitance as the dependent variable is precisely derived and is used for numerical calibration of the analytical relationships with input capacitance as the independent variable and output pressure as the dependent variable in order to meet the capacitive pressure sensor mechanism of detecting pressure by measuring capacitance. Obviously, any touch mode capacitive pressure sensor is the result of continuously increasing pressure on its corresponding non-touch mode capacitive pressure sensor. Thus, in this sense, there are only separate non-touch mode capacitive pressure sensors and no separate non-touch mode capacitive pressure sensors. This is the polymer conductive membrane-based circular capacitive pressure sensor from non-touch mode of operation to touch mode of operation in the title of this paper, whereas the numerical calibrations of the input capacitance–output pressure relationships of non-touch mode circular capacitive pressure sensors use the method presented in reference [47].

This paper is organized as follows. In the following section, the configuration and working principle of a polymer conductive membrane-based circular capacitive pressure sensor from non-touch mode of operation to touch mode of operation is briefly introduced. The accurate analytical relationship with input pressure as the independent variable and output capacitance as the dependent variable is derived in detail. The analytical solution of a circular membrane in frictionless contact with a rigid flat plate under pressure is precisely derived, which is arranged in the Supplementary Materials S.1 for better continuity of the article. Finally, we describe how to design and numerically calibrate the touch mode circular capacitive pressure sensors in detail. In Section 3, an example of how to arrive at the design and numerical calibration of a given (given design parameters) polymer conductive membrane-based circular capacitive pressure sensor from non-touch mode of operation to touch mode of operation is first provided. Then, the influence of changing certain important design parameters on input capacitance-output pressure relationships is comprehensively investigated in order to clarify the direction be which the desired input–output relationships can be approached by changing design parameters. Finally, several important issues are addressed and concluding remarks are given in Section 4.

## 2. Materials and Methods

**Figure 1:**
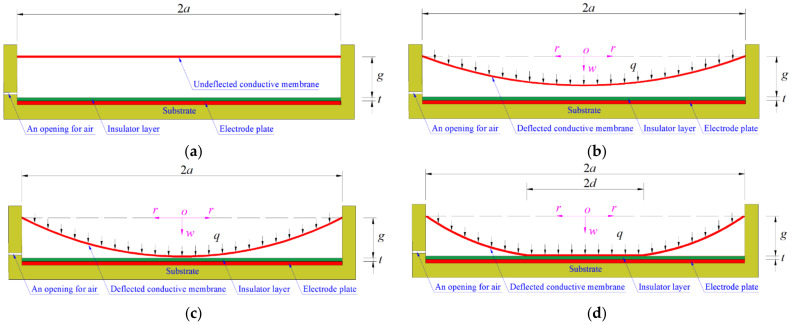
Sketch of a capacitive pressure sensor from non-touch mode of operation to touch mode of operation: (**a**) initial state; (**b**) non-touch mode of operation; (**c**) critical state from non-touch mode of operation to touch mode of operation; (**d**) touch mode of operation.

Figure 1 shows the configuration and working principle of a polymer conductive membrane-based circular capacitive pressure sensor that goes from non-touch mode of operation to touch mode of operation. A circular conductive membrane with radius *a*, thickness *h*, Poisson’s ratio *v* and Young’s modulus of elasticity *E*, which is initially flat and keeps a parallel gap *g* from the insulator layer of thickness *t* coated the substrate electrode plate, is peripherally fixed, thus forming a parallel plate capacitor with air dielectric between the conductive membrane and the insulator layer, as shown in Figure 1a. Under the action of the pressure *q*, the initially flat circular conductive membrane elastically deflects towards the substrate electrode plate but does not touch the insulator layer due to the insufficient pressure *q*, as shown in Figure 1b, and the sensor is said to be in the non-touch mode of operation. When the freely deflecting circular conductive membrane just touches the insulator layer, its maximum deflection stops changing and is equal to the parallel gap *g*, as shown in Figure 1c, and the sensor is in a critical state between non-touch mode of operation to touch mode of operation. If the pressure *q* is further increased, the circular conductive membrane forms a contact radius *d* with the insulation layer, as shown in Figure 1d, and the sensor goes from the non-touch mode of operation to the touch mode of operation. In Figure 1, the dash-dotted line represents the geometric middle plane of the initially flat circular conductive membrane, *o* denotes the origin of the introduced cylindrical coordinate system (*r*, *φ*, *w*), *r* is the radial coordinate, *φ* is the angle coordinate (not represented due to axial symmetry), and *w* is the transverse coordinate and denotes the deflection of the circular conductive membrane under the pressure *q*.

Due to the application of the pressure *q*, the air capacitor between the circular conductive membrane and the insulator layer first changes from the initial parallel plate capacitor in Figure 1a to the non-parallel plate capacitor in Figure 1b, then to the non-parallel plate capacitor in Figure 1d after the critical state of the mode conversion in Figure 1c. Its capacitance is increased, as the air gap between the deflected circular conductive membrane and the insulator layer is decreased from the initial parallel gap *g* to the uneven gap *g*-*w*(*r*). Before the circular conductive membrane touches the insulator layer, the total capacitance *C* of the capacitive pressure sensor operating in non-touch mode consists of the following two parts: one is the capacitance *C*_1_ of the capacitor whose dielectric is the insulator layer, and the other part is the capacitance *C*_2_′ of the air capacitor between the deflected circular conductive membrane and the insulator layer. Because the two capacitors are connected in series, the total capacitance *C* of the capacitive pressure sensor operating in non-touch mode may be written as [47]

(1) $$C=\frac{C_{1}{C^{\prime }}_{2}}{C_{1}+{C^{\prime }}_{2}}=\frac{\frac{\varepsilon_{0}\varepsilon_{r1}\pi a^{2}}{t}2\pi \varepsilon_{0}\varepsilon_{r2}\int_{0}^{a}\frac{r}{g-w(r)}dr}{\frac{\varepsilon_{0}\varepsilon_{r1}\pi a^{2}}{t}+2\pi \varepsilon_{0}\varepsilon_{r2}\int_{0}^{a}\frac{r}{g-w(r)}dr},$$

where *ε*_0_ denotes the vacuum permittivity, *ε_r_*_1_ denotes the relative permittivity of the insulator layer with thickness *t*, and *ε_r_*_2_ denotes the relative permittivity of the air. The detailed derivation of *C*_2_′ in Equation (1) is given in reference [47]. Obviously, before the pressure *q* is applied, the circular conductive membrane is initially flat, and its deflection *w*(*r*) is equal to zero. Therefore, after substituting *w*(*r*) = 0 into Equation (1), the total capacitance *C* of the capacitive pressure sensor without application of pressure is

(2) $$C_{0}=\frac{\frac{\varepsilon_{0}\varepsilon_{r1}\pi a^{2}}{t}2\pi \varepsilon_{0}\varepsilon_{r2}\frac{a^{2}}{2g}}{\frac{\varepsilon_{0}\varepsilon_{r1}\pi a^{2}}{t}+2\pi \varepsilon_{0}\varepsilon_{r2}\frac{a^{2}}{2g}}=\frac{\varepsilon_{0}\varepsilon_{r1}\varepsilon_{r2}\pi a^{2}}{\varepsilon_{r2}t+\varepsilon_{r1}g}.$$

Comparing Equation (2) with Equation (4) in reference [47], we can see that the Equation (4) in reference [47] is incorrect, and there is an error behind its derivation.

After the circular conductive membrane touches the insulator layer, the total capacitance *C* of the capacitive pressure sensor operating in touch mode can be regarded as consisting of the following two parts: one is the capacitance *C′* in the non-contact region of *d* ≤ *r* ≤ *a*, and the other is the capacitance *C*″ in the contact region of 0 ≤ *r* ≤ *d*. The capacitance *C′* consists of two parts: one is the capacitance *C*_1_′ of the insulator layer capacitor within *d* ≤ *r* ≤ *a*, and the other is the capacitance *C*_2_′ of the air capacitor between the deflected circular conductive membrane and the insulator layer within *d* ≤ *r* ≤ *a*. The capacitance *C*_1_′ may be written as

(3) $${C^{\prime }}_{1}=\frac{\varepsilon_{0}\varepsilon_{r1}\pi (a^{2}-d^{2})}{t}.$$

Referring to the derivation of *C*_2_′ in reference [47], it is not difficult to find that the capacitance *C*_2_′can be written as

(4) $${C^{\prime \prime }}_{2}=\int_{d}^{a}\int_{0}^{2\pi }\varepsilon_{0}\varepsilon_{r2}\frac{r}{g-w(r)}d\varphi dr=2\pi \varepsilon_{0}\varepsilon_{r2}\int_{d}^{a}\frac{r}{g-w(r)}dr.$$

As *C*_1_′ and *C*_2_′ are connected in series, the capacitance *C′* in the non-contact region of *d* ≤ *r* ≤ *a* may be written as

(5) $$C^{\prime }=\frac{{C^{\prime }}_{1}{C^{\prime \prime }}_{2}}{{C^{\prime }}_{1}+{C^{\prime \prime }}_{2}}=\frac{\frac{\varepsilon_{0}\varepsilon_{r1}\pi (a^{2}-d^{2})}{t}2\pi \varepsilon_{0}\varepsilon_{r2}\int_{d}^{a}\frac{r}{g-w(r)}dr}{\frac{\varepsilon_{0}\varepsilon_{r1}\pi (a^{2}-d^{2})}{t}+2\pi \varepsilon_{0}\varepsilon_{r2}\int_{d}^{a}\frac{r}{g-w(r)}dr}=\frac{2\pi \varepsilon_{0}\varepsilon_{r1}\varepsilon_{r2}(a^{2}-d^{2})\int_{d}^{a}\frac{r}{g-w(r)}dr}{\varepsilon_{r1}(a^{2}-d^{2})+2t\varepsilon_{r2}\int_{d}^{a}\frac{r}{g-w(r)}dr}.$$

Obviously, in the contact region of 0 ≤ *r* ≤ *d* the air gap between the deflected circular conductive membrane and the insulator layer is equal to zero. Therefore, the capacitance *C*″ in the contact region of 0 ≤ *r* ≤ *d* consists of only one part, which is the capacitance of the insulator layer capacitor within 0 ≤ *r* ≤ *d*, which may be written as

(6) $$C^{\prime \prime }=\varepsilon_{0}\varepsilon_{r1}\pi d^{2}/t.$$

Because *C′* and *C*″ are parallel, the total capacitance *C* of the capacitive pressure sensor operating in touch mode may finally be written as

(7) $$C=C^{\prime }+C^{\prime \prime }=\frac{2\pi \varepsilon_{0}\varepsilon_{r1}\varepsilon_{r2}(a^{2}-d^{2})\int_{d}^{a}\frac{r}{g-w(r)}dr}{\varepsilon_{r1}(a^{2}-d^{2})+2t\varepsilon_{r2}\int_{d}^{a}\frac{r}{g-w(r)}dr}+\frac{\pi \varepsilon_{0}\varepsilon_{r1}d^{2}}{t}.$$

It can be seen by comparing Equation (7) with Equation (1) that Equation (7) can revert to Equation (1) when letting the contact radius *d* in Equation (7) be equal to zero; when letting the deflection *w*(*r*) in Equation (1) be equal to zero, Equation (1) can revert to Equation (2). In other words, the expression for the total capacitance of the capacitive pressure sensor operating in touch mode, Equation (7), can be regressed into the expression for the total capacitance of the capacitive pressure sensor operating in non-touch mode, Equation (1), and can further be regressed into the expression for the total capacitance of the capacitive pressure sensor without application of pressure, Equation (2). This means that the above derivation for Equation (7) is reliable.

Equation (7) shows that the total capacitance *C* of a capacitive pressure sensor operating in touch mode can be determined as long as the analytical expression for the deflection *w*(*r*) of the circular conductive membrane in the non-contact region of *d* ≤ *r* ≤ *a* and the contact radius *d* in the contact region of 0 ≤ *r* ≤ *d* can be determined. Therefore, essential to the design of capacitive pressure sensors operating in touch mode is the analytical solution of the elastic behavior of the circular conductive membrane in axisymmetric contact with the insulator layer. For the continuity of the article, the detailed derivation of the analytical solution of this axisymmetric contact deformation problem is arranged in the Supplementary Materials S.1. The analytical expressions for the stress *σ*(*r*) and deflection *w*(*r*) in the non-contact region of *d* ≤ *r* ≤ *a* can be written as, from Equations (S20), (S29), and (S30),

(8) $$\sigma (r)=E\sum_{i=0}^{\infty }b_{i}{\left(\frac{r}{a}-\frac{d}{2a}-\frac{1}{2}\right)}^{i}$$

and

(9) $$w(r)=a\sum_{i=0}^{\infty }c_{i}{\left(\frac{r}{a}-\frac{d}{2a}-\frac{1}{2}\right)}^{i},$$

where the power series coefficients *bi* (*i* = 2,3,4,…) and *ci* (*i* = 1,2,3,4,…) are listed in the Supplementary Materials S.2 and the remaining coefficients, *b*_0_, *b*_1_, and *c*_0_, including d (or α = d/a), are known as undetermined constants. From the Supplementary Materials S.2, it can be seen that the coefficients *b_i_* (*i* = 2,3,4,…) and *c_i_* (*i* = 1,2,3,4,…) are expressed into the polynomials with regard to the undetermined constants *b*_0_, *b*_1_, and *α*, Poisson’s ratio *v*, initial gap *g*, and dimensionless pressure *Q* (*Q* = *aq*/*Eh*; see Equation (S20). For a given Poisson’s ratio *v*, Young’s modulus of elasticity *E*, thickness *h*, radius *a*, and pressure *q*, the undetermined constants *b*_0_, *b*_1_, and *α* can be determined by simultaneously solving Equations (S33), (S34), and (S37). The contact radius *d* can thus be determined, and the last undetermined constant *c*_0_ can be determined by solving Equation (S31) or (S32) with the known *b*_0_, *b*_1_, and *α*. In this way, the analytical expressions for the stress *σ*(*r*) and deflection *w*(*r*) in the non-contact region of *d* ≤ *r* ≤ *a* can be determined, that is, the contact radius *d* and the power series coefficients *b_i_* and *c_i_* in Equations (8) and (9) are all known. The maximum working stress *σ*_m_ of the circular conductive membrane in axisymmetric contact with the insulator layer is located in the contact region of 0 ≤ *r* ≤ *d*, and hence is given by

(10) $$\sigma_{m}=E\sum_{i=0}^{\infty }b_{i}{\left(\frac{d}{2a}-\frac{1}{2}\right)}^{i}.$$

Therefore, for a given circular conductive membrane, that is, when the Young’s modulus of elasticity *E*, Poisson’s ratio *v*, thickness *h*, radius *a*, and yield strength *σ*_y_ are known in advance, Equation (10) can be used for determining the maximum stress *σ*_m_ at any pressure *q*, assuming that the working stress of the circular conductive membrane is always controlled below 70% of the yield strength *σ*_y_ to ensure the safety requirements of material strength. Thus, if the pressure *q* which is applied onto a given circular conductive membrane and makes the membrane produce a maximum stress *σ*_m_ = 0.7*σ*_y_ is just greater than and close to the maximum pressure of a required pressure measurement range, the given conductive membrane meets the design requirements; otherwise, a new conductive membrane needs to be selected. In this case, the design parameters, such as membrane thickness *h*, Young’s modulus of elasticity *E*, and Poisson’s ratio *v* need to be adjusted. The direction of the adjustment for meeting the required pressure measurement range is provided in the next section.

However, Equation (7) only provides the input–output relationship with pressure as input and capacitance as output, that is, the input pressure-output capacitance relationship with the pressure *q* as the independent variable and the total capacitance *C* as the dependent variable. In order to achieve the sensor mechanism of detecting pressure by measuring capacitance, it is necessary to know the input–output relationship with capacitance as input and pressure as output, that is, the input capacitance–output pressure relationship with the total capacitance *C* as the independent variable and the pressure *q* as the dependent variable. However, we cannot obtain such an input capacitance–output pressure relationship through Equation (7), because the relationship between the pressure *q* and the total capacitance *C* in Equation (7) is strongly nonlinear (the dimensionless pressure *Q* (*Q* = *aq*/*Eh*) is included in the power series coefficients ci of the deflection *w*(*r*); see the Supplementary Materials S.1 and S.2). Therefore, the input capacitance–output pressure relationship with the total capacitance C as the independent variable and the pressure *q* as the dependent variable has to be established by least-squares data fitting based on numerical calculations using Equation (7), which is called numerical calibration and is shown in the next section.

## 3. Results and Discussion

In this section, we provide an example of how to use Equation (7) and the analytical solution in the Supplementary Materials S.1 to realize the design and numerical calibration of a given (given design parameters) polymer conductive membrane-based circular capacitive pressure sensor that goes from non-touch mode of operation to touch mode of operation (see Section 3.1 for details). However, the input capacitance-output pressure relationship with the total capacitance *C* as the independent variable and the pressure *q* as the dependent variable obtained by this numerical calibration, may not meet specific design requirements, especially requirements for the output pressure per unit capacitance and the range of the output pressure *q*. Therefore, if the design requirements are not met, the design parameters need to be changed, including the selection of a circular polymer conductive membrane with other thickness *h*, radius *a*, and Young’s modulus of elasticity *E*. In order to clarify the direction of changing design parameters to arrive at the desired input capacitance-output pressure relationship, the influence of changing design parameters on input capacitance-output pressure relationships is comprehensively investigated here, including changing the initial air parallel gap *g* between the insulator layer coating on the substrate electrode plate and the initially flat undeflected circular conductive membrane as well as changing the membrane thickness *h*, Young’s modulus of elasticity *E*, insulator layer thickness *t*, and the radius *a* of the circular conductive membranes (see Section 3.2 for details).

### 3.1. An Example of Design and Numerical Calibration Based on Analytical Solutions

Assume that a capacitive pressure sensor uses a circular conductive membrane with radius *a* = 100 mm, thickness *h* = 1 mm, Young’s modulus of elasticity *E* = 7.84 MPa, Poisson’s ratio *v* = 0.47, and yield strength *σ*_y_ = 2.4 MPa. If the insulator layer is assumed to take 0.1 mm of polystyrene, then *t* = 0.1 mm and *ε*_r1_ = 2.7. In addition, the vacuum permittivity *ε*_0_ = 8.854 × 10^−3^ pF/mm and the air relative permittivity *ε*_r2_ = 1.00053. From Table 1 in reference [47] it can be seen that the maximum working stress *σ*_m_ of this circular conductive membrane at 21.225 KPa pressure is equal to 1.68 MPa (about 0.7*σ*_y_) and its maximum deflection is about 40 mm. Therefore, the initial air parallel gap *g* can take 10 mm, 20 mm, 30 mm, and 37 mm, respectively, to investigate the influence of changing the initial air parallel gap *g* on the variation trend of input capacitance–output pressure relationships.

Under the action of the pressure *q*, the circular conductive membrane freely elastically deflects towards the insulator layer, then comes into axisymmetric contact with the insulator layer due to the sufficient pressure *q*, as shown in Figure 1. The maximum pressure *q*_m_ that can be applied to the circular conductive membrane can be determined by Equation (10) under the condition of *σ*_m_ = 0.7 *σ*_y_ = 1.68 MPa. If the pressure *q*_m_ is just greater than and close to the maximum pressure of a required pressure measurement range, then the circular conductive membrane is properly selected and meets the design requirements; otherwise, it should be re-selected by changing design parameters such as membrane thickness *h*, Young’s modulus of elasticity *E*, and Poisson’s ratio *v*.

**Figure 2:**
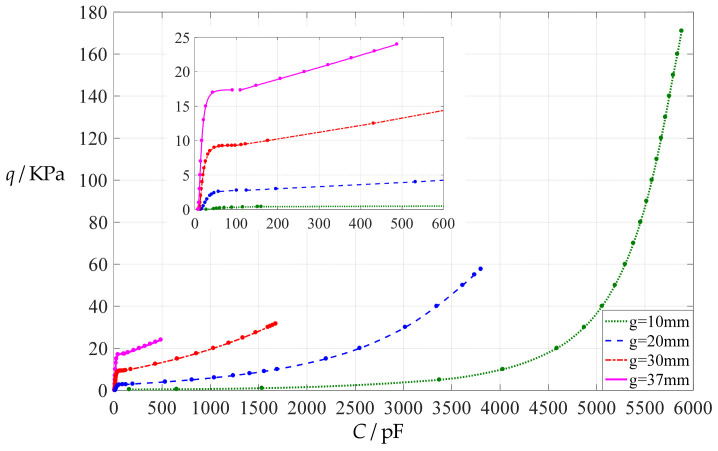
The input capacitance-output pressure relationships of a circular capacitive pressure sensor from non-touch mode of operation to touch mode of operation, where *a* = 100 mm, *h* = 1 mm, *t* = 0.1 mm, *E* = 7.84 MPa, *ν* = 0.47 and the initial air parallel gap *g* takes 10 mm, 20 mm, 30 mm, and 37 mm, respectively.

The total capacitance *C* of this capacitive pressure sensor is determined by Equation (1) before the circular conductive membrane touches the insulator layer and by Equation (7) after the circular conductive membrane touches the insulator layer. The power series expression for the deflection *w*(*r*) in Equation (1) is determined using the analytical solution provided in reference [47], and that for the deflection *w*(*r*) in Equation (7) is determined using the analytical solution given in the Supplementary Materials S.1. The calculation results are listed in Tables S1–S8 in the Supplementary Materials S.3. Figure 2 shows the input capacitance-output pressure relationships with capacitance as the independent variable and pressure as the dependent variable when the initial air parallel gap *g* takes 10 mm, 20 mm, 30 mm, and 37 mm, respectively. It can be seen from Figure 2 that increasing the initial air parallel gap *g* seems to be an effective way to linearize the capacitance-pressure relationships. Moreover, Figure 2 shows that the capacitance–pressure curves are discontinuous at the critical points from the non-touch mode of operation to the touch mode of operation. As such, they cannot be expressed as continuous functions, only as piecewise functions, that is, only piecewise least squares data fitting can be performed.

**Figure 3:**
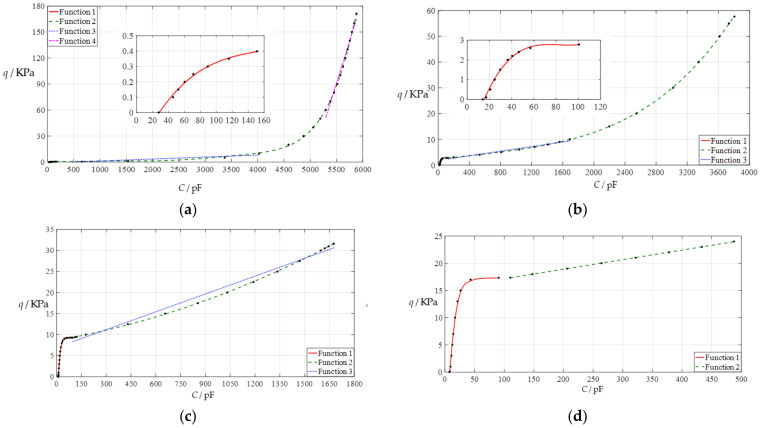
Least-squares fitting of the input capacitance–output pressure relationships in Figure 2: (**a**) for *g* = 10 mm; (**b**) for *g* = 20 mm; (**c**) for *g* = 30 mm; (**d**) for *g* = 37 mm.

Figure 3a shows the results of least-squares fitting for the data when *g* = 10 mm, where Function 1 is the fitting result for the non-touch mode of operation (fitted with a cubic function) and Functions 2, 3, and 4 are the fitting results for the touch mode of operation (fitted with a sixth power function for Function 2 and a straight line for Functions 3 and 4); the function expressions of Functions 1–4 are listed in the row in Table 1 under *g* = 10 mm. Figure 3b shows the results of least-squares fitting for the data when *g* = 20 mm, where Function 1 is the fitting result for the non-touch mode of operation (fitted with a cubic function) and Functions 2 and 3 are the fitting results for the touch mode of operation (fitted with a fourth power function for Function 2 and a straight line for Function 3); the function expressions of Functions 1–3 are listed in the row in Table 1 where *g* = 20 mm is located. Figure 3c shows the results of least-squares fitting for the data when *g* = 30 mm, where Function 1 is the fitting result for the non-touch mode of operation (fitted with a fourth power function) and Functions 2 and 3 are the fitting results for the touch mode of operation (fitted with a quadratic function for Function 2 and a straight line for Function 3); the function expressions of Functions 1–3 are listed in the row in Table 1 where *g* = 30 mm. Figure 3d shows the results of least-squares fitting for the data when *g* = 37 mm, where Function 1 is the fitting result for the non-touch mode of operation (fitted with a cubic function) and Function 2 is the fitting result for the touch mode of operation (fitted with a straight line); the function expressions of Functions 1 and 2 are listed in the row in Table 1 where *g* = 37 mm. In addition, the ranges of the input capacitance and output pressure of each fitting function as well as the average fitting error squares are listed in Table 1.

**Table 1:** The analytical expressions of the fitting Functions in Figure 3, Figure 4, Figure 5 and Figure 6 and the ranges of the input capacitance *C* and output pressure *q*, where *a* = 100 mm, *h* = 1 mm, *t* = 0.1 mm, *E* = 7.84 MPa, *ν* = 0.47, and *g* = 10 mm, 20 mm, 30, mm and 37 mm.

*g*	Functions	Range of *C* (pF)	Range of *q* (KPa)	Analytical Expressions	Average Fitting Error Squares
10 mm	Function 1	27.692~151.298	0~0.399	*q* = 1.398221 × 10^−7^*C*^3^ − 6.490307 × 10^−5^*C*^2^ + 1.099330 × 10^−2^*C* − 2.642701 × 10^−1^	0.0000642
	Function 2	160.066~5880.785	0.4~171	*q* = 1.098169 × 10^−19^*C*^6^ − 1.495897 × 10^−15^*C*^5^ + 7.763423 × 10^−12^*C*^4^ − 1.883144 × 10^−8^*C*^3^ + 2.186898 × 10^−5^*C*^2^ − 1.051067 × 10^−2^*C* + 2.232839	0.9234196
	Function 3	160.066~4028.67	0.4~10	*q* = 2.290661 × 10^−3^*C* − 1.084606	1.8866
	Function 4	5295.641~5880.785	60~171	*q* = 1.943135 × 10^−1^*C* − 977.7616	1.8975
20 mm	Function 1	13.874~100.702	0~2.779	*q* = 9.497546 × 10^−6^*C*^3^ − 2.357107 × 10^−3^*C*^2^ + 1.926258 × 10^−1^*C* − 2.418598	0.007901
	Function 2	124.485~3805.312	2.78~57.65	*q* = 1.492638 × 10^−13^*C*^4^ + 2.648123 × 10^−10^*C*^3^ − 1.962660 × 10^−7^*C*^2^ + 3.323254 × 10^−3^*C* + 2.325193	0.0258063
	Function 3	124.485~1691.536	2.78~10	*q* = 4.462928 × 10^−3^*C* + 1.824869	0.1407778
30 mm	Function 1	9.255~89.325	0~9.293	*q* = −7.477549 × 10^−7^*C*^4^ + 2.178148 × 10^−4^*C*^3^ − 2.319943 × 10^−2^*C*^2^ + 1.071135*C* − 8.876659	0.0703889
	Function 2	97.515~1676.712	9.294~31.64	*q* = 4.077032 × 10^−6^*C*^2^ + 6.863856 × 10^−3^*C* + 8.637091	0.0041431
	Function 3	97.515~1676.712	9.294~31.64	*q* = 1.412844 × 10^−2^*C* + 6.950865	0.9247063
37 mm	Function 1	7.504~43.287	0~17	*q* = 4.488971 × 10^−4^*C*^3^ − 5.453453 × 10^−2^*C*^2^ + 2.257596*C* − 15.20127	0.3464759
	Function 2	110.091~487.552	17.333~23.99	*q* = 1.757338 × 10^−2^*C* + 15.38161	0.0005588

**Figure 4:**
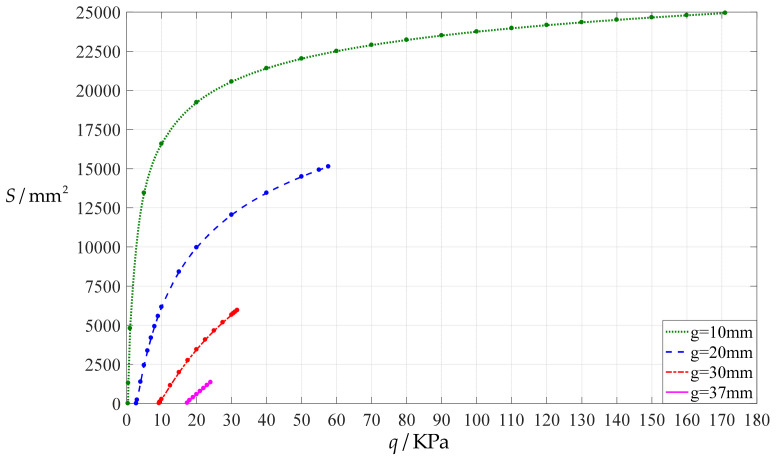
The variations of the contact areas *S* = π*d*^2^ with the pressure *q* for *a* = 100 mm, *h* = 1 mm, *t* = 0.1 mm, *E* = 7.84 MPa, *ν* = 0.47, and *g* = 10 mm, 20 mm, 30 mm, and 37 mm.

Figure 4 shows the variations of the contact areas (*S* = π*d*^2^) with the pressure *q*, indicating that the degree of nonlinearity increases with the decrease of the initial air parallel gap *g*. Figure 4 suggests that the claim that the contact area *S* can be expressed as *S* = π*d*^2^ = *K*_1_*q*^2^ + *K*_2_*q* (*K*_1_ and *K*_2_ are two fitting coefficients [36,37]) is open to question (the average fitting error squares are 2.7257 × 10^7^ when *g* = 10 mm, 6.0300 × 10^5^ when *g* = 20 mm, 2.4512 × 10^5^ when *g* = 30 mm, and 1.6280 × 10^3^ when *g* = 37 mm).

### 3.2. Parametric Analysis

In this section, we address the influence of changing all design parameters (other than the initial air parallel gap *g*) on the variation trend of input capacitance–output pressure relationships, such as changing the thickness *h* and Young’s modulus of elasticity *E* of the circular conductive membranes, the thickness *t* or relative permittivity *ε*_r1_ of the insulator layers (only the thickness *t* is changed here, because the effect of changing the relative permittivity *ε*_r1_ is inversely proportional to the effect of changing the thickness *t*), and the radius *a* of the circular conductive membranes. To this end, we take the design parameters used in Section 3.1 as a reference, that is, circular conductive membrane radius *a* = 100 mm and thickness *h* = 1 mm, Young’s modulus of elasticity *E* = 7.84 MPa, Poisson’s ratio *v* = 0.47, insulator layer thickness *t* = 0.1 mm and relative permittivity *ε*_r1_ = 2.5, vacuum permittivity *ε*_0_ = 8.854 × 10^−12^ F/m = 8.854 × 10^−3^ pF/mm, air relative permittivity *ε*_r2_ = 1.00053, membrane yield stress *σ*_y_ = 2.4 MPa and membrane maximum working stress *σ*_m_ ≤ 0.7 *σ*_y_ ≈ 1.68 MPa; on this basis, we change each parameter one by one. In addition, the research results in Reference [47] show that the change in Poisson’s ratio has a very limited effect on input–output relationships thus, it is not addressed here.

#### 3.2.1. Effect of Membrane Thickness on Capacitance–Pressure Relationships

**Figure 5:**
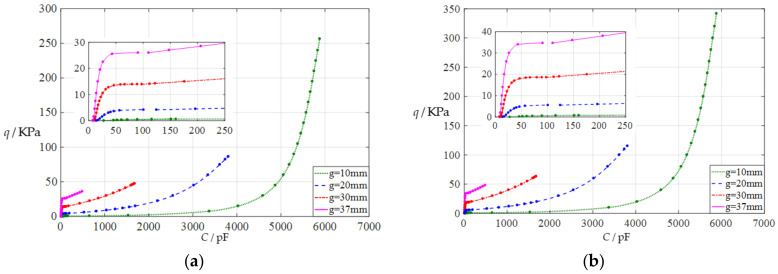
The capacitance-pressure relationships of a circular capacitive pressure sensor from non-touch mode of operation to touch mode of operation: (**a**) for *a* = 100 mm, *h* = 1.5 mm, *t* = 0.1 mm, *E* = 7.84 MPa, *ν* = 0.47, and *g* = 10 mm, 20 mm, 30 mm, and 37 mm; (**b**) for *a* = 100 mm, *h* = 2 mm, *t* = 0.1 mm, *E* = 7.84 MPa, *ν* = 0.47, and *g* = 10 mm, 20 mm, 30 mm, and 37 mm.

In this section, the thickness of the circular conductive membrane is first increased from the reference thickness *h* = 1 mm to *h* = 1.5 mm, then further to *h* = 2 mm. The initial air parallel gap *g* takes 10 mm, 20 mm, 30 mm, and 37 mm. The calculation results can be found in the Supplementary Materials S.4. The input capacitance–output pressure relationships with capacitance as the independent variable and pressure as the dependent variable are shown in Figure 5a for *h* = 1.5 mm and in Figure 5b for *h* = 2 mm. By comparing Figure 2 and Figure 5a,b, it can be found that while increasing the thickness of the circular conductive membrane can increase the range of the output pressure *q*, the range of the input capacitance *C* is almost unchanged, which can be seen more clearly in the Supplementary Materials S.4. Therefore, increasing the thickness of the circular conductive membrane increases the output pressure per unit capacitance to an extent, as the range of the output pressure *q* increases while the range of the input capacitance *C* remains constant.

#### 3.2.2. Effect of Young’s Modulus of Elasticity on Capacitance-Pressure Relationships

**Figure 6:**
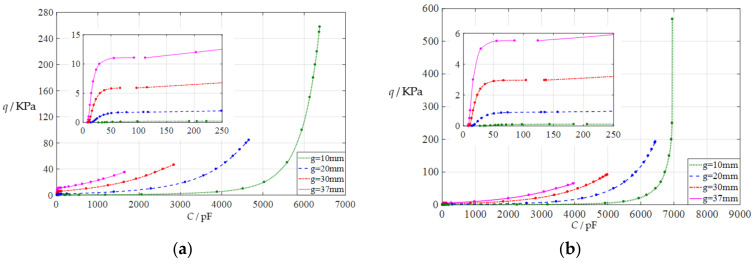
The capacitance–pressure relationships of a circular capacitive pressure sensor from non-touch mode of operation to touch mode of operation: (**a**) for *a* = 100 mm, *h* = 1 mm, *t* = 0.1 mm, *E* = 5 MPa, *ν* = 0.47, and *g* = 10 mm, 20 mm, 30 mm, and 37 mm; (**b**) for *a* = 100 mm, *h* = 1 mm, *t* = 0.1 mm, *E* = 2.5 MPa, *ν* = 0.47, and *g* = 10 mm, 20 mm, 30 mm, and 37 mm.

In this section, the Young’s modulus of elasticity *E* of the circular conductive membrane is first decreased from the reference value *E* = 7.84 MPa to *E* = 5 MPa, then further to *E* = 2.5 MPa. The initial air parallel gap g takes 10 mm, 20 mm, 30 mm, and 37 mm. The calculation results can be found in the Supplementary Materials S.5. The input capacitance–output pressure relationships with capacitance as independent variable and pressure as dependent variable are shown in Figure 6a for *E* = 5 MPa and in Figure 6b for *E* = 2.5 MPa. By comparing Figure 2 and Figure 6a,b, it can be found that decreasing the Young’s modulus of elasticity *E* of the circular conductive membrane can increase both the range of the output pressure *q* and the range of the input capacitance C, which can be seen more clearly from the Supplementary Materials S.5. Therefore, the effect of decreasing the Young’s modulus of elasticity *E* on the output pressure per unit capacitance is uncertain.

#### 3.2.3. Effect of Thickness of Insulator Layer on Capacitance–Pressure Relationships

**Figure 7:**
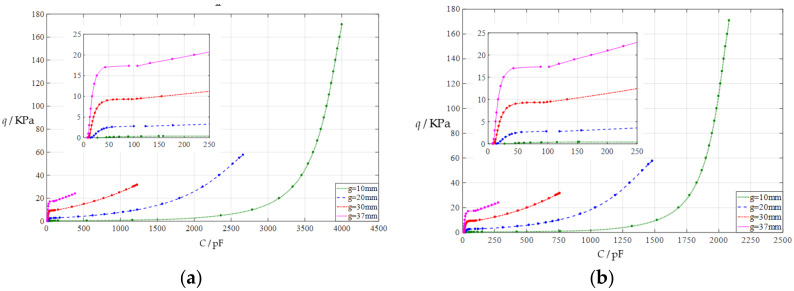
The capacitance–pressure relationships of a circular capacitive pressure sensor from non-touch mode of operation to touch mode of operation: (**a**) for *a* = 100 mm, *h* = 1 mm, *t* = 0.15 mm, *E* = 7.84 MPa, *ν* = 0.47, and *g* = 10 mm, 20 mm, 30 mm, and 37 mm; (**b**) for *a* = 100 mm, *h* = 1 mm, *t* = 0.3 mm, *E* = 7.84 MPa, *ν* = 0.47, and *g* = 10 mm, 20 mm, 30 mm, and 37 mm.

In this section, the insulator layer thickness *t* is first increased from the reference value *t* = 0.1 mm to *t* = 0.15 mm, then further to *t* = 0.3 mm. The initial air parallel gap *g* takes 10 mm, 20 mm, 30 mm, and 37 mm. The calculation results can be found in the Supplementary Materials S.6. The input capacitance-output pressure relationships with capacitance as independent variable and pressure as dependent variable are shown in Figure 7a for *t* = 0.15 mm and in Figure 7b for *t* = 0.3 mm. By comparing Figure 2 and Figure 7a,b, it can be found that increasing the insulator layer thickness *t* can only increase the range of the input capacitance *C*, while the range of the output pressure q is almost unchanged, which can be seen more clearly in the Supplementary Materials S.6. Therefore, increasing the insulator layer thickness *t* can decrease the output pressure per unit capacitance, because the range of the input capacitance *C* increases while the range of the output pressure *q* remains constant.

#### 3.2.4. Effect of Membrane Radius on Capacitance-Pressure Relationships

**Figure 8:**
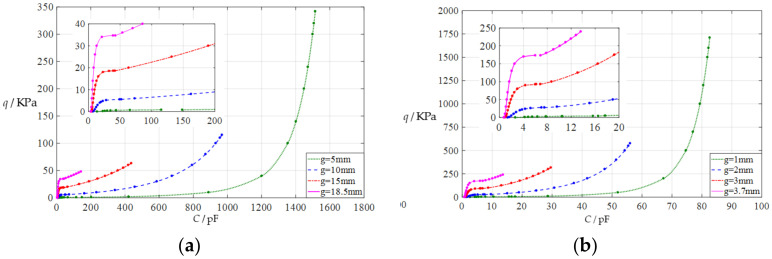
The capacitance–pressure relationships of a circular capacitive pressure sensor from non-touch mode of operation to touch mode of operation: (**a**) for *a* = 50 mm, *h* = 1 mm, *t* = 0.1 mm, *E* = 7.84 MPa, *ν* = 0.47, and *g* = 5 mm, 10 mm, 15 mm, and 18.5 mm; (**b**) for *a* = 10 mm, *h* = 1 mm, *t* = 0.1 mm, *E* = 7.84 MPa, *ν* = 0.47, and *g* = 1 mm, 2 mm, 3 mm, and 3.7 mm.

In this section, the radius *a* of the circular conductive membrane is first decreased from the reference value *a* = 100 mm to *a* = 50 mm, then further to *a* = 10 mm. The initial air parallel gap *g* takes 5 mm, 10 mm, 15 mm, and 18.5 mm for *a* = 50 mm, and takes 1 mm, 2 mm, 3 mm, and 3.7 mm for a = 10 mm. The calculation results can be found in the Supplementary Materials S.7. The input capacitance-output pressure relationships with capacitance as the independent variable and pressure as the dependent variable is shown in Figure 8a for *a* = 50 mm and in Figure 8b for *a* = 10 mm. By comparing Figure 2 and Figure 8a,b, it can be found that decreasing the membrane radius *a* can increase the range of the output pressure *q* but decreases the range of the input capacitance C, which can be seen more clearly in the Supplementary Materials S.7. Therefore, decreasing the membrane radius *a* can greatly increase the output pressure per unit capacitance, because the range of the output pressure *q* increases since the thickness *h* of the circular conductive membrane is kept constant at 1 mm, while the range of the input capacitance *C* is greatly decreased because the area of the circular conductive membrane (movable electrode plate) is greatly reduced.

**Figure 9:**
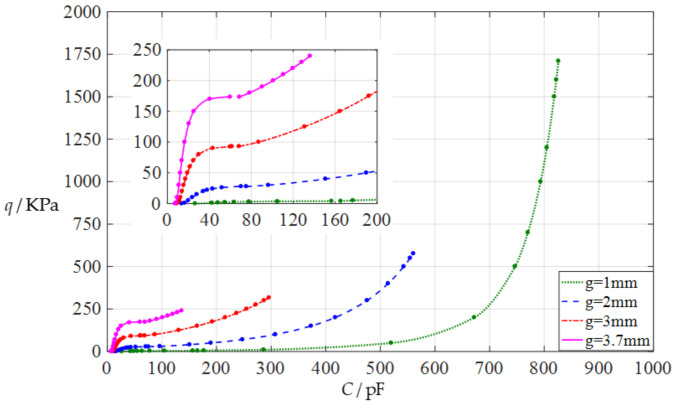
The capacitance–pressure relationships of ten parallel circular capacitive pressure sensors from non-touch mode of operation to touch mode of operation, where *a* = 10 mm, *h* = 1 mm, *t* = 0.1 mm, *E* = 7.84 MPa, *ν* = 0.47, and *g* = 1 mm, 2 mm, 3 mm, and 3.7 mm.

In addition, small-volume capacitive pressure sensors are often used in parallel in many applications, especially integrated ones [18,19,20]. This application can incidentally reduce the output pressure per unit capacitance. For example, ten circular capacitive pressure sensors of *a* = 10 mm could be used in parallel (a sensor array); the corresponding capacitance–pressure relationships are shown in Figure 9. Comparing Figure 8b and Figure 9, it can be found that the range of the input capacitance *C* is increased by a factor of 10 while the range of the output pressure *q* is kept constant. Therefore, it can be calculated from Tables S65–S72 in the Supplementary Materials S.7 that the output pressure per unit capacitance decreases from 21.385 KPa/pF to 2.1385 KPa/pF for *g* = 1 mm, from 10.541 KPa/pF to 1.0541 KPa/pF for *g* = 2 mm, from 11.029 KPa/pF to 1.1029 KPa/pF for *g* = 3 mm, and from 18.647 KPa/pF to 1.8647 KPa/pF for *g* = 3.7 mm.

## 4. Concluding Remarks

In this paper, an analytical solution-based method for the design and numerical calibration of polymer conductive membrane-based circular capacitive pressure sensors from non-touch mode of operation to touch mode of operation is presented for the first time. This novel method can provide effective theoretical support for the design and fabrication of such capacitive pressure sensors. It should be pointed out that due to the lack of accurate analytical solutions for design research, there has always been a meaningless debate over which is better, non-touch mode of operation or touch mode of operation. However, as can be seen from this study, both non-touch mode of operation and touch mode of operation can meet user requirements as long as the analytical solution-based method proposed in this paper is adopted for design and numerical calibration. For instance, the use requirement to avoid touch mode of operation can be achieved by increasing the initial air parallel gap *g* or Young’s modulus of elasticity *E* (assuming the pressure *q* remains constant). From this study, the following conclusions can be drawn.

The input capacitance-output pressure relationships of polymer conductive membrane-based circular capacitive pressure sensors are discontinuous at the critical points from the non-touch mode of operation to the touch mode of operation, and thus can only be expressed as two piecewise functions.

The increase of the initial air parallel gap *g* can effectively linearize the input capacitance–output pressure relationships of the sensors, including non-touch mode of operation and touch mode of operation.

The increase of the thickness *h* of the conductive membranes can increase the range of the output pressure *q*, but does not change the range of the input capacitance *C*, resulting in the increase of the output pressure per unit capacitance.

The decrease of the Young’s modulus of elasticity *E* of the conductive membranes can increase both the range of the output pressure *q* and the range of the input capacitance *C*, making little change in the output pressure per unit capacitance.

The increase of the thickness *t* of the insulator layers can only increase the range of the input capacitance *C* and does not change the range of the output pressure *q*, thus decreasing the output pressure per unit capacitance.

The decrease of the radius *a* of the conductive membranes can increase the range of the output pressure *q* and decrease the range of the input capacitance *C*, thus greatly increasing the output pressure per unit capacitance. However, small-volume capacitive pressure sensors are often used in parallel in many applications, especially integrated ones, which can incidentally reduce the output pressure per unit capacitance.

Finally, we would like to talk about the implications of this study for polymer research. As can be seen from this study, a capacitive pressure sensor can be designed to meet the specified use requirements only by adjusting the design parameters over a wide range, which is particularly important when adjusting the Young’s modulus of elasticity *E* of the conductive membranes, including the relative permittivity *ε_r_*_1_ of the insulator layer (its effect is inversely proportional to the effect of adjusting the thickness *t* of the insulator layer). Therefore, the significance of this study to polymer researches mainly lies in the proposal of a targeted application research direction for polymer research, that is, to carry out the study of polymer or polymer-based conductive membranes with a wider range of Young’s modulus of elasticity *E*, including polymer coatings (used as the insulator layer) with a wider range of relative permittivity *ε_r_*_1_.

## Data Availability

Not applicable.

## Supplementary Materials

The following supporting information can be downloaded at: https://www.mdpi.com/article/10.3390/polym14183850/s1, S.1: Membrane Equations and Its Solution; S.2: Recursive Relations for Power Series Coefficients; S.3: An Example of Design and Numerical Calibration Based on Analytical Solutions; S.4: Effect of Membrane Thickness on Capacitance–Pressure Relationships; S.5: Effect of Young’s Modulus of Elasticity on Capacitance–Pressure Relationships; S.6: Effect of Thickness of Insulator Layer on Capacitance–Pressure Relationships; S.7: Effect of Membrane Radius on Capacitance–Pressure Relationships. References [3,5,6,47] are cited in the supplementary materials.
