# Supplementary material for: Polymer Conductive Membrane-Based Circular Capacitive Pressure Sensors from Non-Touch Mode of Operation to Touch Mode of Operation: An Analytical Solution-Based Method for Design and Numerical Calibration
Source: Polymers (Basel). 2022 Sep 14;14(18):3850. doi: 10.3390/polym14183850 (PMC9501485; doi:10.3390/polym14183850)
Supplement: Supplementary file 1 [file polymers-14-03850-s001.zip › polymers-1889554-supplementary.pdf]

polymers

Article

Polymer Conductive Membrane-Based Circular Capacitive Pressure Sensors from Non-Touch Mode of Operation to Touch Mode of Operation: An Analytical Solution-Based Method for Design and Numerical Calibration

Qi Zhang [1], Fei-Yan Li [1], Xue Li [1], Xiao-Ting He [1,2] and Jun-Yi Sun [1,2,*]

[1] School of Civil Engineering, Chongqing University, Chongqing 400045, China
[2] Key Laboratory of New Technology for Construction of Cities in Mountain Area (Chongqing University), Ministry of Education, Chongqing 400045, China
* Correspondence: sunjunyi@cqu.edu.cn; Tel.: +86-(0)23-65120720

SUPPLEMENTARY MATERIALS

S.1. Membrane Equations and Its Solution

An initially flat, peripherally fixed, linearly elastic circular membrane with Poisson's ratio ν, Young's modulus of elasticity E, thickness h, and radius a is subjected to a pressure q. When the pressure q reaches a large enough value, the deflected circular membrane will come in contact with a frictionless rigid flat plate being parallel to the initially flat circular membrane, as shown in Figure S1, where r is the radial coordinate, w is the transversal displacement, d is the contact radius between the deflected circular membrane contacting and the frictionless rigid plate, and g is the parallel gap between the frictionless rigid plate and the initially flat circular membrane. Such a contact problem can be viewed as consisting of two local membrane problems in the central portion of $0 \le r \le d$ and in the annular portion of $d \le r \le a$, which are connected by the continuity conditions at $r=d$. The problem in $0 \le r \le d$ may be simplified as a plane stretching problem, while the problem in $d \le r \le a$ is the large deflection problem of an annular membrane under the pressure q. In the annular portion of $d \le r \le a$, let us take a piece of the circular membrane with radius r ($d \le r \le a$) from the central portion of the deflected whole circular membrane (that is, this piece of the circular membrane includes the deflected circular membrane in the contact region $0 \le r \le d$), to study the static problem of equilibrium of this piece of the circular membrane, as shown in Figure S2, where σ_r is the radial stress, and θ is the rotation angle of the deflected circular membrane.

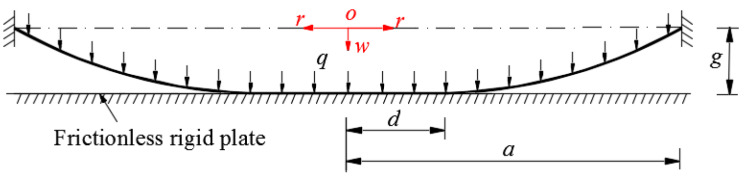

Figure S1. Sketch of a deflected circular membrane in contact with a frictionless rigid plate.

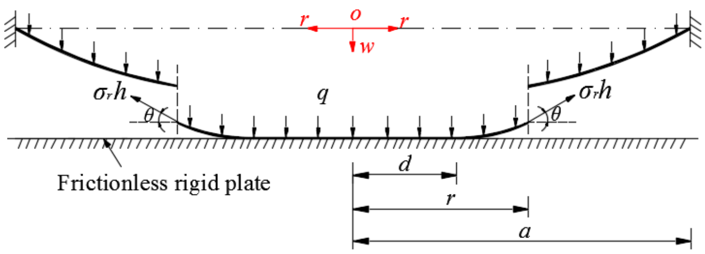

Figure S2. Sketch of the static equilibrium of the deflected circular membrane with radius $d \le r \le a$.

In the vertical direction perpendicular to the initially flat circular membrane (see Figure S2), there are three vertical forces, the vertical force $2\pi r\sigma_r h\sin\theta$ produced by the membrane force $\sigma_r h$, the total reaction force $\pi d^2 q$ from the frictionless rigid plate, and the total action force $\pi r^2 q$ of the pressure q within radius r, where $d \le r \le a$. So, the equilibrium condition in the vertical direction is

$$2\pi r\sigma_r h\sin\theta + \pi d^2 q = \pi r^2 q\,, \tag{S1}$$

where

$$\sin\theta = 1/\sqrt{1+1/\tan^2\theta} = 1/\sqrt{1+1/(-\mathrm{d}w/\mathrm{d}r)^2}\;. \tag{S2}$$

Substituting Equation (S2) into Equation (S1) one has

$$2r\sigma_r h = (r^2 - d^2)q\sqrt{1+1/(\mathrm{d}w/\mathrm{d}r)^2}\;. \tag{S3}$$

If the circumferential stress is denoted by σ_t, then the equilibrium condition in the horizontal direction parallel to the initially flat circular membrane may be written as [5]

$$\frac{\mathrm{d}}{\mathrm{d}r}(r\sigma_r h) - \sigma_t h = 0\,. \tag{S4}$$

If the radial and circumferential strain, and the radial displacement are respectively expressed as e_r, e_t and u, then the geometric equations for large deflection problems may be written as [6]

$$e_r = [(1+\frac{\mathrm{d}u}{\mathrm{d}r})^2 + (\frac{\mathrm{d}w}{\mathrm{d}r})^2]^{1/2} - 1 \tag{S5}$$

and

$$e_t = \frac{u}{r}\,. \tag{S6}$$

Moreover, the relationships between stress and strain, the physical equations, still satisfy the generalized Hooke's law and are given by [3]

$$\sigma_r = \frac{E}{1-\nu^2}(e_r + \nu e_t) \tag{S7}$$

and

$$\sigma_t = \frac{E}{1-\nu^2}(e_t + \nu e_r)\,. \tag{S8}$$

Substituting Equations (S5) and (S6) into Equations (S7) and (S8) yields

$$\sigma_r = \frac{E}{1-\nu^2}\{[(1+\frac{\mathrm{d}u}{\mathrm{d}r})^2 + (\frac{\mathrm{d}w}{\mathrm{d}r})^2]^{1/2} - 1 + \nu\frac{u}{r}\} \tag{S9}$$

and

$$\sigma_t = \frac{E}{1-\nu^2}\{\frac{u}{r} + \nu[(1+\frac{\mathrm{d}u}{\mathrm{d}r})^2 + (\frac{\mathrm{d}w}{\mathrm{d}r})^2]^{1/2} - \nu\}. \tag{S10}$$

By means of Equations (S4), (S9) and (S10), one has

$$\frac{u}{r} = \frac{1}{E}(\sigma_t - \nu\sigma_r) = \frac{1}{E}[\frac{\mathrm{d}}{\mathrm{d}r}(r\sigma_r) - \nu\sigma_r]. \tag{S11}$$

Substituting the u of Equation (S11) into Equation (S9) yields

$$[\frac{1}{E}\sigma_r - \frac{v}{E}\frac{\mathrm{d}}{\mathrm{d}r}(r\sigma_r) + 1]^2 - \{\frac{1}{E}\frac{\mathrm{d}}{\mathrm{d}r}[r\frac{\mathrm{d}}{\mathrm{d}r}(r\sigma_r)] - \frac{v}{E}\frac{\mathrm{d}}{\mathrm{d}r}(r\sigma_r) + 1\}^2 - (\frac{\mathrm{d}w}{\mathrm{d}r})^2 = 0. \tag{S12}$$

In the plate/membrane contact region of the central portion ($0 \le r \le d$), the strain and stress are always uniformly distributed [5], that is,

$$e_r = e_t = \frac{u(d)}{d} \tag{S13}$$

and

$$\sigma_r = \sigma_t = \frac{E}{1-\nu}\frac{u(d)}{d}. \tag{S14}$$

So, the boundary conditions at $r=a$ and the continuous conditions at $r=d$ are

$$w = 0 \quad \text{at} \quad r = a, \tag{S15}$$

$$u = 0 \quad \text{at} \quad r = a \tag{S16}$$

and

$$w = g \quad \text{at} \quad r = d, \tag{S17}$$

$$(\frac{u}{r})_A = (\frac{u}{r})_B = \frac{u(d)}{d} \quad \text{at} \quad r = d, \tag{S18}$$

$$(\sigma_r)_A = (\sigma_r)_B = \frac{E}{1-\nu}\frac{u(d)}{d} \quad \text{at} \quad r = d, \tag{S19}$$

where the subscripts A and B denote the two sides of the inter-connecting circle of $r=d$, A refers the side to plate/membrane contact, and B refers the side to plate/membrane non-contact.

Introduce the following dimensionless variables

$$Q = \frac{qa}{Eh}, \quad W = \frac{w}{a}, \quad S_r = \frac{\sigma_r}{E}, \quad S_t = \frac{\sigma_t}{E}, \quad x = \frac{r}{a}, \quad \alpha = \frac{d}{a}, \tag{S20}$$

and transform Equations (S3), (S12), (S4), (S15), (S16), (S17), (S18) and (S19) into

$$[4x^2 S_r{}^2 - Q^2(x^2-\alpha^2)^2](\frac{\mathrm{d}W}{\mathrm{d}x})^2 - Q^2(x^2-\alpha^2)^2 = 0, \tag{S21}$$

$$[S_r - v\frac{\mathrm{d}}{\mathrm{d}x}(xS_r) + 1]^2 - \{\frac{\mathrm{d}}{\mathrm{d}x}[x\frac{\mathrm{d}}{\mathrm{d}x}(xS_r)] - v\frac{\mathrm{d}}{\mathrm{d}x}(xS_r) + 1\}^2 - (\frac{\mathrm{d}W}{\mathrm{d}x})^2 = 0, \tag{S22}$$

$$S_t = S_r + x\frac{\mathrm{d}S_r}{\mathrm{d}x}, \quad \text{(S23)}$$

$$W = 0 \text{ at } x = 1, \quad \text{(S24)}$$

$$S_t - \nu S_r = 0 \text{ at } x = 1 \quad \text{(S25)}$$

and

$$W = \frac{g}{a} \text{ at } x = \alpha, \quad \text{(S26)}$$

$$(S_t - \nu S_r)_A = (S_t - \nu S_r)_B = \frac{u(b)}{b} \text{ at } x = \alpha, \quad \text{(S27)}$$

$$(S_r)_A = (S_r)_B = \frac{1}{1-\nu}\frac{u(b)}{b} \text{ at } x = \alpha. \quad \text{(S28)}$$

Expanding S_r and W into the power series of x-(1+α)/2, i.e., letting

$$S_r = \sum_{i=0}^{\infty} b_i (x - \frac{1+\alpha}{2})^i, \quad \text{(S29)}$$

$$W = \sum_{i=0}^{\infty} c_i (x - \frac{1+\alpha}{2})^i. \quad \text{(S30)}$$

Substituting Equations (S29) and (S30) into Equations (S21) and (S22), the power series coefficients c_i (i=2,3,4,…) and d_i (i=1,2,3,4,…) can be expressed by the polynomial of b_0, b_1 and α, which are listed in this Supplementary Materials S.2. The coefficient c_0 is another unknown constant.

The remaining three coefficients b_0, b_1 and c_0 and the dimensionless variable α (α = d/a) are usually known as undetermined constants, and can be determined by using the boundary conditions and continuous conditions as follows. From Equation (S30), Equations (S24) and (S26) give

$$\sum_{i=0}^{\infty} c_i (\frac{1-\alpha}{2})^i = 0 \quad \text{(S31)}$$

and

$$\sum_{i=0}^{\infty} c_i (\frac{\alpha-1}{2})^i = \frac{g}{a}. \quad \text{(S32)}$$

Equation (S32) minus Equation (S31) yields

$$\sum_{i=1}^{\infty} c_i [(\frac{\alpha-1}{2})^i - (\frac{1-\alpha}{2})^i] = \frac{g}{a}. \quad \text{(S33)}$$

From Equations (S23) and (S29), Equations (S25), (S27) and (S28) yield

$$(1-\nu)\sum_{i=0}^{\infty} b_i (\frac{1-\alpha}{2})^i + \sum_{i=1}^{\infty} i b_i (\frac{1-\alpha}{2})^{i-1} = 0, \quad \text{(S34)}$$

$$(1-\nu)\sum_{i=0}^{\infty} b_i (\frac{\alpha-1}{2})^i + \alpha\sum_{i=1}^{\infty} i b_i (\frac{\alpha-1}{2})^{i-1} = \frac{u(d)}{d} \quad \text{(S35)}$$

and

$$\sum_{i=0}^{\infty} b_i (\frac{\alpha-1}{2})^i = \frac{1}{1-\nu} \frac{u(d)}{d} . \tag{S36}$$

Eliminating the $u(d)/d$ from Equations (S35) and (S36), one has

$$\alpha \sum_{i=1}^{\infty} i b_i (\frac{\alpha-1}{2})^{i-1} = 0 . \tag{S37}$$

For the given problem where a, h, E, υ, g and q are known in advance, the undetermined constants b_0, b_1 and α can be determined by the simultaneous solutions of Equations (S33), (S34) and (S37). Furthermore, substituting the known b_0, b_1 and α into Equation (S31) or Equation (S32), the last undetermined constant c_0 can also be determined. The problem dealt with here is thus solved.

S.2. Recursive Relations for Power Series Coefficients

$$\begin{aligned} b_2 = {} & \frac{1}{2\beta^2}(\beta\nu b_1 - 3\beta b_1 + \nu b_0 - b_0 - 1 + (\beta^2\nu^2 b_1^2 + 2\beta\nu^2 b_0 b_1 - 2\beta\nu b_0 b_1 + \nu^2 b_0^2 - 2\beta\nu b_1 \\ & -2\nu b_0^2 - 2\nu b_0 + b_0^2 - c_1^2 + 2b_0 + 1)^{1/2}) \end{aligned} ,$$

$$\begin{aligned} b_3 = {} & -\frac{1}{6\beta^2(2\beta^2 b_2 - \beta\nu b_1 + 3\beta b_1 - \nu b_0 + b_0 + 1)}(20\beta^3 b_2^2 - 4\beta^3\nu b_2^2 - 20\beta^2\nu b_1 b_2 \\ & +38\beta^2 b_1 b_2 - 10\beta\nu b_0 b_2 - 9\beta\nu b_1^2 + 10\beta b_0 b_2 + 12\beta b_1^2 - 3\nu b_0 b_1 + 10\beta b_2 + 3b_0 b_1 \\ & +2c_1 c_2 + 3b_1) \end{aligned} ,$$

$$\begin{aligned} b_4 = {} & -\frac{1}{24\beta^2(2\beta^2 b_2 - \beta\nu b_1 + 3\beta b_1 - \nu b_0 + b_0 + 1)}(36\beta^4 b_3^2 - 36\beta^3\nu b_2 b_3 + 204\beta^3 b_2 b_3 \\ & -84\beta^2\nu b_1 b_3 - 52\beta^2\nu b_2^2 + 174\beta^2 b_1 b_3 + 136\beta^2 b_2^2 - 42\beta\nu b_0 b_3 - 86\beta\nu b_1 b_2 + 42\beta b_0 b_3 \\ & +134\beta b_1 b_2 - 16\nu b_0 b_2 - 12\nu b_1^2 + 42\beta b_3 + 16b_0 b_2 + 15b_1^2 + 6c_1 c_3 + 4c_2^2 + 16b_2) \end{aligned} ,$$

$$\begin{aligned} b_5 = {} & -\frac{1}{20\beta^2(2\beta^2 b_2 - \beta\nu b_1 + 3\beta b_1 - \nu b_0 + b_0 + 1)}(72\beta^4 b_3 b_4 - 32\beta^3\nu b_2 b_4 - 18\beta^3\nu b_3^2 \\ & +192\beta^3 b_2 b_4 + 126\beta^3 b_3^2 - 72\beta^2\nu b_1 b_4 - 98\beta^2\nu b_2 b_3 + 156\beta^2 b_1 b_4 + 296\beta^2 b_2 b_3 \\ & -36\beta\nu b_0 b_4 - 78\beta\nu b_1 b_3 - 46\beta\nu b_2^2 + 36\beta b_0 b_4 + 132\beta b_1 b_3 + 90\beta b_2^2 - 15\nu b_0 b_3 \\ & -25\nu b_1 b_2 + 36\beta b_4 + 15b_0 b_3 + 35b_1 b_2 + 4c_1 c_4 + 6c_2 c_3 + 15b_3) \end{aligned} ,$$

$$\begin{aligned} b_6 = {} & -\frac{1}{60\beta^2(2\beta^2 b_2 - \beta\nu b_1 + 3\beta b_1 - \nu b_0 + b_0 + 1)}(240\beta^4 b_3 b_5 + 144\beta^4 b_4^2 - 100\beta^3\nu b_2 b_5 \\ & -120\beta^3\nu b_3 b_4 + 620\beta^3 b_2 b_5 + 936\beta^3 b_3 b_4 - 220\beta^2\nu b_1 b_5 - 316\beta^2\nu b_2 b_4 - 174\beta^2\nu b_3^2 \\ & +490\beta^2 b_1 b_5 + 1036\beta^2 b_2 b_4 + 633\beta^2 b_3^2 - 110\beta\nu b_0 b_5 - 246\beta\nu b_1 b_4 - 314\beta\nu b_2 b_3 \\ & +110\beta b_0 b_5 + 438\beta b_1 b_4 + 698\beta b_2 b_3 - 48\nu b_0 b_4 - 84\nu b_1 b_3 - 48\nu b_2^2 + 110\beta b_5 + 48b_0 b_4 \\ & +126b_1 b_3 + 80b_2^2 + 10c_1 c_5 + 16c_2 c_4 + 9c_3^2 + 48b_4) \end{aligned} ,$$

$$\begin{aligned} b_7 = {} & -\frac{1}{42\beta^2(2\beta^2 b_2 - \beta\nu b_1 + 3\beta b_1 - \nu b_0 + b_0 + 1)}(180\beta^4 b_3 b_6 + 240\beta^4 b_4 b_5 - 72\beta^3\nu b_2 b_6 \\ & -90\beta^3\nu b_3 b_5 - 48\beta^3\nu b_4^2 + 456\beta^3 b_2 b_6 + 750\beta^3 b_3 b_5 + 432\beta^3 b_4^2 - 156\beta^2\nu b_1 b_6 - 78\beta\nu b_0 b_6 \\ & -232\beta^2\nu b_2 b_5 - 270\beta^2\nu b_3 b_4 + 354\beta^2 b_1 b_6 + 802\beta^2 b_2 b_5 + 1098\beta^2 b_3 b_4 + 12c_3 c_4 - 35\nu b_0 b_5 \\ & -178\beta\nu b_1 b_5 - 238\beta\nu b_2 b_4 - 129\beta\nu b_3^2 + 78\beta b_0 b_6 + 328\beta b_1 b_5 + 574\beta b_2 b_4 + 336\beta b_3^2 \\ & -63\nu b_1 b_4 - 77\nu b_2 b_3 + 78\beta b_6 + 35b_0 b_5 + 99b_1 b_4 + 143b_2 b_3 + 6c_1 c_6 + 10c_2 c_5 + 35b_5) \end{aligned} ,$$

$$b_8=-\frac{1}{112\beta^2(2\beta^2b_2-\beta\nu b_1+3\beta b_1-\nu b_0+b_0+1)}(504\beta^4b_3b_7+720\beta^4b_4b_6+400\beta^4b_5^{\ 2}$$
$$-196\beta^3\nu b_2b_7-252\beta^3\nu b_3b_6-280\beta^3\nu b_4b_5+1260\beta^3b_2b_7+2196\beta^3b_3b_6+2760\beta^3b_4b_5$$
$$-420\beta^2\nu b_1b_7-640\beta^2\nu b_2b_6-772\beta^2\nu b_3b_5-408\beta^2\nu b_4^{\ 2}+966\beta^2b_1b_7+2296\beta^2b_2b_6$$
$$+3382\beta^2b_3b_5+1896\beta^2b_4^{\ 2}-210\beta\nu b_0b_7-486\beta\nu b_1b_6-670\beta\nu b_2b_5-762\beta\nu b_3b_4 \quad ,$$
$$+210\beta b_0b_7+918\beta b_1b_6+1710\beta b_2b_5+2202\beta b_3b_4-96\nu b_0b_6-176\nu b_1b_5-224\nu b_2b_4$$
$$-120\nu b_3^{\ 2}+210\beta b_7+96b_0b_6+286b_1b_5+448b_2b_4+255b_3^{\ 2}+14c_1c_7+24c_2c_6$$
$$+30c_3c_5+16c_4^{\ 2}+96b_6)$$

$$b_9=-\frac{1}{72\beta^2(2\beta^2b_2-\beta\nu b_1+3\beta b_1-\nu b_0+b_0+1)}(336\beta^4b_3b_8+504\beta^4b_4b_7+600\beta^4b_5b_6$$
$$-128\beta^3\nu b_2b_8-168\beta^3\nu b_3b_7-192\beta^3\nu b_4b_6-100\beta^3\nu b_5^{\ 2}+832\beta^3b_2b_8+1512\beta^3b_3b_7$$
$$+2016\beta^3b_4b_6+1100\beta^3b_5^{\ 2}-272\beta^2\nu b_1b_8-422\beta^2\nu b_2b_7-522\beta^2\nu b_3b_6-572\beta^2\nu b_4b_5$$
$$+632\beta^2b_1b_8+1556\beta^2b_2b_7+2412\beta^2b_3b_6+2912\beta^2b_4b_5-136\beta\nu b_0b_8-318\beta\nu b_1b_7 \quad ,$$
$$-448\beta\nu b_2b_6-526\beta\nu b_3b_5-276\beta\nu b_4^{\ 2}+136\beta b_0b_8+612\beta b_1b_7+1192\beta b_2b_6$$
$$+1636\beta b_3b_5+900\beta b_4^{\ 2}-63\nu b_0b_7-117\nu b_1b_6-153\nu b_2b_5-171\nu b_3b_4+136\beta b_8$$
$$+63b_0b_7+195b_1b_6+323b_2b_5+399b_3b_4+8c_1c_8+14c_2c_7+18c_3c_6+20c_4c_5+63b_7)$$

$$b_{10}=-\frac{1}{180\beta^2(2\beta^2b_2-\beta\nu b_1+3\beta b_1-\nu b_0+b_0+1)}(864\beta^4b_3b_9+1344\beta^4b_4b_8$$
$$+1680\beta^4b_5b_7+900\beta^4b_6^{\ 2}+2124\beta^3b_2b_9+3984\beta^3b_3b_8+5544\beta^3b_4b_7+6420\beta^3b_5b_6$$
$$-790\beta^2\nu b_5^{\ 2}+1602\beta^2b_1b_9+4052\beta^2b_2b_8+6522\beta^2b_3b_7+8292\beta^2b_4b_6+4465\beta^2b_5^{\ 2}$$
$$+342\beta b_0b_9+1574\beta b_1b_8+3170\beta b_2b_7+4554\beta b_3b_6+5342\beta b_4b_5-160\nu b_0b_8$$
$$-300\nu b_1b_7-400\nu b_2b_6-460\nu b_3b_5-240\nu b_4^{\ 2}+342\beta b_9+160b_0b_8+510b_1b_7 \quad ,$$
$$+880b_2b_6+1150b_3b_5+624b_4^{\ 2}+18c_1c_9+32c_2c_8+42c_3c_7+48c_4c_6+160b_8$$
$$-324\beta^3\nu b_2b_9-432\beta^3\nu b_3b_8-504\beta^3\nu b_4b_7-540\beta^3\nu b_5b_6-684\beta^2\nu b_1b_9$$
$$-1076\beta^2\nu b_2b_8-1356\beta^2\nu b_3b_7-1524\beta^2\nu b_4b_6-342\beta\nu b_0b_9-806\beta\nu b_1b_8$$
$$-1154\beta\nu b_2b_7-1386\beta\nu b_3b_6-1502\beta\nu b_4b_5+25c_5^{\ 2})$$

$$c_1=-\frac{\eta Q}{\sqrt{-Q^2\eta^2+4\beta^2b_0^{\ 2}}},$$

$$c_2=-\frac{1}{c_1(Q^2\eta^2-4\beta^2b_0^{\ 2})}\beta(Q^2\eta c_1^{\ 2}-2\beta b_0b_1c_1^{\ 2}-2b_0^{\ 2}c_1^{\ 2}+Q^2\eta),$$

$$c_3=-\frac{1}{3c_1\left(Q^2\eta^2-4\beta^2b_0^{\ 2}\right)}(2Q^2\beta^2c_1^{\ 2}+8Q^2\beta\eta c_1c_2+2Q^2\eta^2c_2^{\ 2}-8\beta^2b_0^{\ 2}c_2^{\ 2}-16\beta^2b_0b_1c_1c_2 \quad ,$$
$$-4\beta^2b_0b_2c_1^{\ 2}-2\beta^2b_1^{\ 2}c_1^{\ 2}+Q^2\eta c_1^{\ 2}-16\beta b_0^{\ 2}c_1c_2-8\beta b_0b_1c_1^{\ 2}+2Q^2\beta^2-2b_0^{\ 2}c_1^{\ 2}+Q^2\eta)$$

$$c_4=-\frac{1}{2c_1(Q^2\eta^2-4\beta^2b_0^{\ 2})}(4Q^2\beta^2c_1c_2+6Q^2\beta\eta c_1c_3+4Q^2\beta\eta c_2^{\ 2}+3Q^2\eta^2c_2c_3$$
$$-12\beta^2b_0^{\ 2}c_2c_3-12\beta^2b_0b_1c_1c_3-8\beta^2b_0b_1c_2^{\ 2}-8\beta^2b_0b_2c_1c_2-2\beta^2b_0b_3c_1^{\ 2}-4\beta^2b_1^{\ 2}c_1c_2 \quad ,$$
$$-2\beta^2b_1b_2c_1^{\ 2}+Q^2\beta c_1^{\ 2}+2Q^2\eta c_1c_2-12\beta b_0^{\ 2}c_1c_3-8\beta b_0^{\ 2}c_2^{\ 2}-16\beta b_0b_1c_1c_2-4\beta b_0b_2c_1^{\ 2}$$
$$-2\beta b_1^{\ 2}c_1^{\ 2}-4b_0^{\ 2}c_1c_2-2b_0b_1c_1^{\ 2}+Q^2\beta)$$

$$
\begin{aligned}
c_5 = & -\frac{1}{10c_1\left(Q^2\eta^2-4\beta^2 b_0{}^2\right)}(24Q^2\beta^2c_1c_3+16Q^2\beta^2c_2{}^2+32Q^2\beta\eta c_1c_4+48Q^2\beta\eta c_2c_3\\
& +16Q^2\eta^2c_2c_4+9Q^2\eta^2c_3{}^2-64\beta^2b_0{}^2c_2c_4-36\beta^2b_0{}^2c_3{}^2-64\beta^2b_0b_1c_1c_4-96\beta^2b_0b_1c_2c_3\\
& -48\beta^2b_0b_2c_1c_3-32\beta^2b_0b_2c_2{}^2-32\beta^2b_0b_3c_1c_2-8\beta^2b_0b_4c_1{}^2-24\beta^2b_1{}^2c_1c_3-16\beta^2b_1{}^2c_2{}^2\\
& -32\beta^2b_1b_2c_1c_2-8\beta^2b_1b_3c_1{}^2-4\beta^2b_2{}^2c_1{}^2+16Q^2\beta c_1c_2+12Q^2\eta c_1c_3+8Q^2\eta c_2{}^2\\
& -64\beta b_0{}^2c_1c_4-96\beta b_0{}^2c_2c_3-96\beta b_0b_1c_1c_3-64\beta b_0b_1c_2{}^2-64\beta b_0b_2c_1c_2-16\beta b_0b_3c_1{}^2\\
& -32\beta b_1{}^2c_1c_2-16\beta b_1b_2c_1{}^2+Q^2c_1{}^2-24b_0{}^2c_1c_3-16b_0{}^2c_2{}^2-32b_0b_1c_1c_2-8b_0b_2c_1{}^2\\
& -4b_1{}^2c_1{}^2+Q^2),
\end{aligned}
$$

$$
\begin{aligned}
c_6 = & -\frac{1}{3c_1(Q^2\beta^2-4\beta^2b_0{}^2)}(8Q^2\beta^2c_1c_4+12Q^2\beta^2c_2c_3+10Q^2\beta\eta c_1c_5+16Q^2\beta\eta c_2c_4\\
& +9Q^2\beta\eta c_3{}^2+5Q^2\eta^2c_2c_5+6Q^2\eta^2c_3c_4-20\beta^2b_0{}^2c_2c_5-24\beta^2b_0{}^2c_3c_4-20\beta^2b_0b_1c_1c_5\\
& -32\beta^2b_0b_1c_2c_4-18\beta^2b_0b_1c_3{}^2-16\beta^2b_0b_2c_1c_4-24\beta^2b_0b_2c_2c_3-12\beta^2b_0b_3c_1c_3\\
& -8\beta^2b_0b_3c_2{}^2-8\beta^2b_0b_4c_1c_2-2\beta^2b_0b_5c_1{}^2-8\beta^2b_1{}^2c_1c_4-12\beta^2b_1{}^2c_2c_3-12\beta^2b_1b_2c_1c_3\\
& -8\beta^2b_1b_2c_2{}^2-8\beta^2b_1b_3c_1c_2-2\beta^2b_1b_4c_1{}^2-4\beta^2b_2{}^2c_1c_2-2\beta^2b_2b_3c_1{}^2+6Q^2\beta c_1c_3\\
& +4Q^2\beta c_2{}^2+4Q^2\eta c_1c_4+6Q^2\eta c_2c_3-20\beta b_0{}^2c_1c_5-32\beta b_0{}^2c_2c_4-18\beta b_0{}^2c_3{}^2\\
& -32\beta b_0b_1c_1c_4-48\beta b_0b_1c_2c_3-24\beta b_0b_2c_1c_3-16\beta b_0b_2c_2{}^2-16\beta b_0b_3c_1c_2-4\beta b_0b_4c_1{}^2\\
& -12\beta b_1{}^2c_1c_3-8\beta b_1{}^2c_2{}^2-16\beta b_1b_2c_1c_2-4\beta b_1b_3c_1{}^2-2\beta b_2{}^2c_1{}^2+Q^2c_1c_2-8b_0{}^2c_1c_4\\
& -12b_0{}^2c_2c_3-12b_0b_1c_1c_3-8b_0b_1c_2{}^2-8b_0b_2c_1c_2-2b_0b_3c_1{}^2-4b_1{}^2c_1c_2-2b_1b_2c_1{}^2),
\end{aligned}
$$

$$
\begin{aligned}
c_7 = & -\frac{1}{7c_1(Q^2\eta^2-4\beta^2b_0{}^2)}(20Q^2\beta^2c_1c_5+32Q^2\beta^2c_2c_4+18Q^2\beta^2c_3{}^2+24Q^2\beta\eta c_1c_6\\
& +40Q^2\beta\eta c_2c_5+48Q^2\beta\eta c_3c_4+12Q^2\eta^2c_2c_6+15Q^2\eta^2c_3c_5+8Q^2\eta^2c_4{}^2-48\beta^2b_0{}^2c_2c_6\\
& -60\beta^2b_0{}^2c_3c_5-32\beta^2b_0{}^2c_4{}^2-48\beta^2b_0b_1c_1c_6-80\beta^2b_0b_1c_2c_5-96\beta^2b_0b_1c_3c_4\\
& -40\beta^2b_0b_2c_1c_5-64\beta^2b_0b_2c_2c_4-36\beta^2b_0b_2c_3{}^2-32\beta^2b_0b_3c_1c_4-48\beta^2b_0b_3c_2c_3\\
& -24\beta^2b_0b_4c_1c_3-16\beta^2b_0b_4c_2{}^2-16\beta^2b_0b_5c_1c_2-4\beta^2b_0b_6c_1{}^2-20\beta^2b_1{}^2c_1c_5-32\beta^2b_1{}^2c_2c_4\\
& -18\beta^2b_1{}^2c_3{}^2-32\beta^2b_1b_2c_1c_4-48\beta^2b_1b_2c_2c_3-24\beta^2b_1b_3c_1c_3-16\beta^2b_1b_3c_2{}^2-16\beta^2b_1b_4c_1c_2\\
& -4\beta^2b_1b_5c_1{}^2-12\beta^2b_2{}^2c_1c_3-8\beta^2b_2{}^2c_2{}^2-16\beta^2b_2b_3c_1c_2-4\beta^2b_2b_4c_1{}^2-2\beta^2b_3{}^2c_1{}^2\\
& +16Q^2\beta c_1c_4+24Q^2\beta c_2c_3+10Q^2\eta c_1c_5+16Q^2\eta c_2c_4+9Q^2\eta c_3{}^2-48\beta b_0{}^2c_1c_6\\
& -80\beta b_0{}^2c_2c_5-96\beta b_0{}^2c_3c_4-80\beta b_0b_1c_1c_5-128\beta b_0b_1c_2c_4-72\beta b_0b_1c_3{}^2-64\beta b_0b_2c_1c_4\\
& -96\beta b_0b_2c_2c_3-48\beta b_0b_3c_1c_3-32\beta b_0b_3c_2{}^2-32\beta b_0b_4c_1c_2-8\beta b_0b_5c_1{}^2-32\beta b_1{}^2c_1c_4\\
& -48\beta b_1{}^2c_2c_3-48\beta b_1b_2c_1c_3-32\beta b_1b_2c_2{}^2-32\beta b_1b_3c_1c_2-8\beta b_1b_4c_1{}^2-16\beta b_2{}^2c_1c_2\\
& -8\beta b_2b_3c_1{}^2+3Q^2c_1c_3+2Q^2c_2{}^2-20b_0{}^2c_1c_5-32b_0{}^2c_2c_4-18b_0{}^2c_3{}^2-32b_0b_1c_1c_4\\
& -48b_0b_1c_2c_3-24b_0b_2c_1c_3-16b_0b_2c_2{}^2-16b_0b_3c_1c_2-4b_0b_4c_1{}^2-12b_1{}^2c_1c_3-8b_1{}^2c_2{}^2\\
& -16b_1b_2c_1c_2-4b_1b_3c_1{}^2-2b_2{}^2c_1{}^2),
\end{aligned}
$$

$$
\begin{aligned}
c_8 = -\frac{1}{4c_1(Q^2\eta^2 - 4\beta^2 {b_0}^2)}&(12Q^2\beta^2 c_1 c_6 + 20Q^2\beta^2 c_2 c_5 + 24Q^2\beta^2 c_3 c_4 + 14Q^2\beta\eta c_1 c_7 \\
&+24Q^2\beta\eta c_2 c_6 + 30Q^2\beta\eta c_3 c_5 + 16Q^2\beta\eta {c_4}^2 + 7Q^2\eta^2 c_2 c_7 + 9Q^2\eta^2 c_3 c_6 + 10Q^2\eta^2 c_4 c_5 \\
&-28\beta^2 {b_0}^2 c_2 c_7 - 36\beta^2 {b_0}^2 c_3 c_6 - 40\beta^2 {b_0}^2 c_4 c_5 - 28\beta^2 b_0 b_1 c_1 c_7 - 48\beta^2 b_0 b_1 c_2 c_6 \\
&-60\beta^2 b_0 b_1 c_3 c_5 - 32\beta^2 b_0 b_1 {c_4}^2 - 24\beta^2 b_0 b_2 c_1 c_6 - 40\beta^2 b_0 b_2 c_2 c_5 - 48\beta^2 b_0 b_2 c_3 c_4 \\
&-20\beta^2 b_0 b_3 c_1 c_5 - 32\beta^2 b_0 b_3 c_2 c_4 - 18\beta^2 b_0 b_3 {c_3}^2 - 16\beta^2 b_0 b_4 c_1 c_4 - 24\beta^2 b_0 b_4 c_2 c_3 \\
&-12\beta^2 b_0 b_5 c_1 c_3 - 8\beta^2 b_0 b_5 {c_2}^2 - 8\beta^2 b_0 b_6 c_1 c_2 - 2\beta^2 b_0 b_7 {c_1}^2 - 12\beta^2 {b_1}^2 c_1 c_6 - 20\beta^2 {b_1}^2 c_2 c_5 \\
&-24\beta^2 {b_1}^2 c_3 c_4 - 20\beta^2 b_1 b_2 c_1 c_5 - 32\beta^2 b_1 b_2 c_2 c_4 - 18\beta^2 b_1 b_2 {c_3}^2 - 16\beta^2 b_1 b_3 c_1 c_4 \\
&-24\beta^2 b_1 b_3 c_2 c_3 - 12\beta^2 b_1 b_4 c_1 c_3 - 8\beta^2 b_1 b_4 {c_2}^2 - 8\beta^2 b_1 b_5 c_1 c_2 - 2\beta^2 b_1 b_6 {c_1}^2 - 8\beta^2 {b_2}^2 c_1 c_4 \\
&-12\beta^2 {b_2}^2 c_2 c_3 - 12\beta^2 b_2 b_3 c_1 c_3 - 8\beta^2 b_2 b_3 {c_2}^2 - 8\beta^2 b_2 b_4 c_1 c_2 - 2\beta^2 b_2 b_5 {c_1}^2 - 4\beta^2 {b_3}^2 c_1 c_2 \\
&-2\beta^2 b_3 b_4 {c_1}^2 + 10Q^2\beta c_1 c_5 + 16Q^2\beta c_2 c_4 + 9Q^2\beta {c_3}^2 + 6Q^2\eta c_1 c_6 + 10Q^2\eta c_2 c_5 \\
&+12Q^2\eta c_3 c_4 - 28\beta {b_0}^2 c_1 c_7 - 48\beta {b_0}^2 c_2 c_6 - 60\beta {b_0}^2 c_3 c_5 - 32\beta {b_0}^2 {c_4}^2 - 48\beta b_0 b_1 c_1 c_6 \\
&-80\beta b_0 b_1 c_2 c_5 - 96\beta b_0 b_1 c_3 c_4 - 40\beta b_0 b_2 c_1 c_5 - 64\beta b_0 b_2 c_2 c_4 - 36\beta b_0 b_2 {c_3}^2 - 32\beta b_0 b_3 c_1 c_4 \\
&-48\beta b_0 b_3 c_2 c_3 - 24\beta b_0 b_4 c_1 c_3 - 16\beta b_0 b_4 {c_2}^2 - 16\beta b_0 b_5 c_1 c_2 - 4\beta b_0 b_6 {c_1}^2 - 20\beta {b_1}^2 c_1 c_5 \\
&-32\beta {b_1}^2 c_2 c_4 - 18\beta {b_1}^2 {c_3}^2 - 32\beta b_1 b_2 c_1 c_4 - 48\beta b_1 b_2 c_2 c_3 - 24\beta b_1 b_3 c_1 c_3 - 16\beta b_1 b_3 {c_2}^2 \\
&-16\beta b_1 b_4 c_1 c_2 - 4\beta b_1 b_5 {c_1}^2 - 12\beta {b_2}^2 c_1 c_3 - 8\beta {b_2}^2 {c_2}^2 - 16\beta b_2 b_3 c_1 c_2 - 4\beta b_2 b_4 {c_1}^2 \\
&-2\beta {b_3}^2 {c_1}^2 + 2Q^2 c_1 c_4 + 3Q^2 c_2 c_3 - 12{b_0}^2 c_1 c_6 - 20{b_0}^2 c_2 c_5 - 24{b_0}^2 c_3 c_4 - 20 b_0 b_1 c_1 c_5 \\
&-32 b_0 b_1 c_2 c_4 - 18 b_0 b_1 {c_3}^2 - 16 b_0 b_2 c_1 c_4 - 24 b_0 b_2 c_2 c_3 - 12 b_0 b_3 c_1 c_3 - 8 b_0 b_3 {c_2}^2 \\
&-8 b_0 b_4 c_1 c_2 - 2 b_0 b_5 {c_1}^2 - 8{b_1}^2 c_1 c_4 - 12{b_1}^2 c_2 c_3 - 12 b_1 b_2 c_1 c_3 - 8 b_1 b_2 {c_2}^2 - 8 b_1 b_3 c_1 c_2 \\
&-2 b_1 b_4 {c_1}^2 - 4{b_2}^2 c_1 c_2 - 2 b_2 b_3 {c_1}^2)
\end{aligned}
$$

$$
\begin{aligned}
c_9 = &-\frac{1}{18c_1(Q^2\eta^2-4\beta^2{b_0}^2)}(56Q^2\beta^2c_1c_7+96Q^2\beta^2c_2c_6+120Q^2\beta^2c_3c_5+64Q^2\beta^2{c_4}^2 \\
&+64Q^2\beta\eta c_1c_8+112Q^2\beta\eta c_2c_7+144Q^2\beta\eta c_3c_6+160Q^2\beta\eta c_4c_5+32Q^2\eta^2c_2c_8+42Q^2\eta^2c_3c_7 \\
&+48Q^2\eta^2c_4c_6+25Q^2\eta^2{c_5}^2-128\beta^2{b_0}^2c_2c_8-168\beta^2{b_0}^2c_3c_7-192\beta^2{b_0}^2c_4c_6-100\beta^2{b_0}^2{c_5}^2 \\
&-128\beta^2b_0b_1c_1c_8-224\beta^2b_0b_1c_2c_7-288\beta^2b_0b_1c_3c_6-320\beta^2b_0b_1c_4c_5-112\beta^2b_0b_2c_1c_7 \\
&-192\beta^2b_0b_2c_2c_6-240\beta^2b_0b_2c_3c_5-128\beta^2b_0b_2{c_4}^2-96\beta^2b_0b_3c_1c_6-160\beta^2b_0b_3c_2c_5 \\
&-192\beta^2b_0b_3c_3c_4-80\beta^2b_0b_4c_1c_5-128\beta^2b_0b_4c_2c_4-72\beta^2b_0b_4{c_3}^2-64\beta^2b_0b_5c_1c_4-96\beta^2b_0b_5c_2c_3 \\
&-48\beta^2b_0b_6c_1c_3-32\beta^2b_0b_6{c_2}^2-32\beta^2b_0b_7c_1c_2-8\beta^2b_0b_8{c_1}^2-56\beta^2{b_1}^2c_1c_7-96\beta^2{b_1}^2c_2c_6 \\
&-120\beta^2{b_1}^2c_3c_5-64\beta^2{b_1}^2{c_4}^2-96\beta^2b_1b_2c_1c_6-160\beta^2b_1b_2c_2c_5-192\beta^2b_1b_2c_3c_4-80\beta^2b_1b_3c_1c_5 \\
&-128\beta^2b_1b_3c_2c_4-72\beta^2b_1b_3{c_3}^2-64\beta^2b_1b_4c_1c_4-96\beta^2b_1b_4c_2c_3-48\beta^2b_1b_5c_1c_3-32\beta^2b_1b_5{c_2}^2 \\
&-32\beta^2b_1b_6c_1c_2-8\beta^2b_1b_7{c_1}^2-40\beta^2{b_2}^2c_1c_5-64\beta^2{b_2}^2c_2c_4-36\beta^2{b_2}^2{c_3}^2-64\beta^2b_2b_3c_1c_4 \\
&-96\beta^2b_2b_3c_2c_3-48\beta^2b_2b_4c_1c_3-32\beta^2b_2b_4{c_2}^2-32\beta^2b_2b_5c_1c_2-8\beta^2b_2b_6{c_1}^2-24\beta^2{b_3}^2c_1c_3 \\
&-16\beta^2{b_3}^2{c_2}^2-32\beta^2b_3b_4c_1c_2-8\beta^2b_3b_5{c_1}^2-4\beta^2{b_4}^2{c_1}^2+48Q^2\beta c_1c_6+80Q^2\beta c_2c_5+96Q^2\beta c_3c_4 \\
&+28Q^2\eta c_1c_7+48Q^2\eta c_2c_6+60Q^2\eta c_3c_5+32Q^2\eta{c_4}^2-128\beta{b_0}^2c_1c_8-224\beta{b_0}^2c_2c_7 \\
&-288\beta{b_0}^2c_3c_6-320\beta{b_0}^2c_4c_5-224\beta b_0b_1c_1c_7-384\beta b_0b_1c_2c_6-480\beta b_0b_1c_3c_5-256\beta b_0b_1{c_4}^2 \\
&-192\beta b_0b_2c_1c_6-320\beta b_0b_2c_2c_5-384\beta b_0b_2c_3c_4-160\beta b_0b_3c_1c_5-256\beta b_0b_3c_2c_4-144\beta b_0b_3{c_3}^2 \\
&-128\beta b_0b_4c_1c_4-192\beta b_0b_4c_2c_3-96\beta b_0b_5c_1c_3-64\beta b_0b_5{c_2}^2-64\beta b_0b_6c_1c_2-16\beta b_0b_7{c_1}^2 \\
&-96\beta{b_1}^2c_1c_6-160\beta{b_1}^2c_2c_5-192\beta{b_1}^2c_3c_4-160\beta b_1b_2c_1c_5-256\beta b_1b_2c_2c_4-144\beta b_1b_2{c_3}^2 \\
&-128\beta b_1b_3c_1c_4-192\beta b_1b_3c_2c_3-96\beta b_1b_4c_1c_3-64\beta b_1b_4{c_2}^2-64\beta b_1b_5c_1c_2-16\beta b_1b_6{c_1}^2 \\
&-64\beta{b_2}^2c_1c_4-96\beta{b_2}^2c_2c_3-96\beta b_2b_3c_1c_3-64\beta b_2b_3{c_2}^2-64\beta b_2b_4c_1c_2-16\beta b_2b_5{c_1}^2 \\
&-32\beta{b_3}^2c_1c_2-16\beta b_3b_4{c_1}^2+10Q^2c_1c_5+16Q^2c_2c_4+9Q^2{c_3}^2-56{b_0}^2c_1c_7-96{b_0}^2c_2c_6 \\
&-120{b_0}^2c_3c_5-64{b_0}^2{c_4}^2-96b_0b_1c_1c_6-160b_0b_1c_2c_5-192b_0b_1c_3c_4-80b_0b_2c_1c_5-128b_0b_2c_2c_4 \\
&-72b_0b_2{c_3}^2-64b_0b_3c_1c_4-96b_0b_3c_2c_3-48b_0b_4c_1c_3-32b_0b_4{c_2}^2-32b_0b_5c_1c_2-8b_0b_6{c_1}^2 \\
&-40{b_1}^2c_1c_5-64{b_1}^2c_2c_4-36{b_1}^2{c_3}^2-64b_1b_2c_1c_4-96b_1b_2c_2c_3-48b_1b_3c_1c_3-32b_1b_3{c_2}^2 \\
&-32b_1b_4c_1c_2-8b_1b_5{c_1}^2-24{b_2}^2c_1c_3-16{b_2}^2{c_2}^2-32b_2b_3c_1c_2-8b_2b_4{c_1}^2-4{b_3}^2{c_1}^2)
\end{aligned}
,
$$

$$
\begin{aligned}
c_{10} = &-\frac{1}{5c_1(Q^2\eta^2-4\beta^2{b_0}^2)}(16Q^2\beta^2c_1c_8+28Q^2\beta^2c_2c_7+36Q^2\beta^2c_3c_6+40Q^2\beta^2c_4c_5 \\
&+18Q^2\beta\eta c_1c_9+32Q^2\beta\eta c_2c_8+42Q^2\beta\eta c_3c_7+48Q^2\beta\eta c_4c_6+25Q^2\beta\eta{c_5}^2+9Q^2\eta^2c_2c_9 \\
&+12Q^2\eta^2c_3c_8+14Q^2\eta^2c_4c_7+15Q^2\eta^2c_5c_6-36\beta^2{b_0}^2c_2c_9-48\beta^2{b_0}^2c_3c_8-56\beta^2{b_0}^2c_4c_7 \\
&-60\beta^2{b_0}^2c_5c_6-36\beta^2b_0b_1c_1c_9-64\beta^2b_0b_1c_2c_8-84\beta^2b_0b_1c_3c_7-96\beta^2b_0b_1c_4c_6-50\beta^2b_0b_1{c_5}^2 \\
&-32\beta^2b_0b_2c_1c_8-56\beta^2b_0b_2c_2c_7-72\beta^2b_0b_2c_3c_6-80\beta^2b_0b_2c_4c_5-28\beta^2b_0b_3c_1c_7-48\beta^2b_0b_3c_2c_6 \\
&+14Q^2\eta c_2c_7-64\beta b_0b_2{c_4}^2-60\beta{b_1}^2c_3c_5-18\beta^2b_1b_4{c_3}^2-8b_1b_4{c_2}^2-20\beta^2b_1b_4c_1c_5-48\beta b_0b_5c_2c_3 \\
&-32\beta{b_2}^2c_2c_4-32\beta b_0b_5c_1c_4-24\beta b_2b_4c_1c_3-48\beta{b_1}^2c_2c_6-28\beta^2b_1b_2c_1c_7-80\beta b_1b_2c_2c_5 \\
&-24\beta b_1b_5c_1c_3-112\beta b_0b_1c_2c_7-4\beta b_2b_6{c_1}^2-24\beta^2b_2b_4c_2c_3-2\beta{b_4}^2{c_1}^2-40\beta b_1b_3c_1c_5-2b_2b_5{c_1}^2 \\
&-48\beta b_1b_2c_1c_6-12\beta{b_3}^2c_1c_3-16b_1b_3c_1c_4-40\beta^2b_0b_4c_2c_5-48\beta^2b_0b_4c_3c_4-20\beta^2b_0b_5c_1c_5 \\
&-24b_1b_3c_2c_3-28b_0b_1c_1c_7-24b_0b_4c_2c_3-16\beta b_1b_6c_1c_2+18Q^2\eta c_3c_6-16\beta^2b_2b_4c_1c_4-96\beta b_0b_2c_2c_6
\end{aligned}
,
$$

$$
\begin{aligned}
&-2\beta^2 b_4 b_5 c_1{}^2 + 14Q^2\beta c_1 c_7 - 12b_2 b_3 c_1 c_3 - 20b_1 b_2 c_1 c_5 - 16\beta b_3 b_4 c_1 c_2 - 40\beta^2 b_1 b_3 c_2 c_5 - 12b_1 b_4 c_1 c_3 \\
&-12\beta^2 b_2{}^2 c_1 c_6 - 20\beta^2 b_2{}^2 c_2 c_5 - 32b_1 b_2 c_2 c_4 - 96\beta b_0{}^2 c_4 c_6 - 20b_1{}^2 c_2 c_5 - 48\beta b_2 b_3 c_2 c_3 - 60b_0 b_1 c_3 c_5 \\
&-64\beta b_0{}^2 c_2 c_8 + 30Q^2\beta c_3 c_5 - 24\beta b_0 b_6 c_1 c_3 - 32\beta b_1{}^2 c_4{}^2 - 16\beta^2 b_0 b_6 c_1 c_4 - 24\beta^2 b_0 b_6 c_2 c_3 \\
&-8\beta^2 b_1 b_7 c_1 c_2 - 48\beta b_0 b_3 c_1 c_6 + 20Q^2\eta c_4 c_5 - 8\beta^2 b_0 b_8 c_1 c_2 - 12\beta^2 b_2 b_5 c_1 c_3 - 24b_0 b_2 c_1 c_6 \\
&-2\beta^2 b_3 b_6 c_1{}^2 - 56\beta b_0 b_2 c_1 c_7 - 8b_2{}^2 c_1 c_4 - 32b_0 b_1 c_4{}^2 - 24\beta^2 b_1 b_3 c_1 c_6 - 16\beta b_2 b_4 c_2{}^2 - 8\beta^2 b_2 b_5 c_2{}^2 \\
&-4b_3{}^2 c_1 c_2 - 8b_2 b_4 c_1 c_2 - 8\beta^2 b_3 b_5 c_1 c_2 - 48\beta^2 b_1 b_3 c_3 c_4 - 28b_0{}^2 c_2 c_7 - 32b_0 b_3 c_2 c_4 - 24\beta^2 b_1 b_5 c_2 c_3 \\
&-32\beta^2 b_2 b_3 c_2 c_4 - 18\beta^2 b_2 b_3 c_3{}^2 - 16b_0 b_4 c_1 c_4 - 12b_0 b_5 c_1 c_3 - 36\beta b_0{}^2 c_1 c_9 - 18b_0 b_3 c_3{}^2 - 8b_0 b_5 c_2{}^2 \\
&-48\beta^2 b_1 b_2 c_2 c_6 + 24Q^2\beta c_2 c_6 - 64\beta b_0 b_1 c_1 c_8 - 20\beta b_2{}^2 c_1 c_5 - 32\beta^2 b_1 b_4 c_2 c_4 - 28\beta b_1{}^2 c_1 c_7 \\
&-8b_1 b_5 c_1 c_2 - 20b_0 b_3 c_1 c_5 + 5Q^2 c_2 c_5 + 6Q^2 c_3 c_4 - 16b_0{}^2 c_1 c_8 - 18b_1 b_2 c_3{}^2 - 48b_0 b_2 c_3 c_4 - 8b_2 b_3 c_2{}^2 \\
&-36\beta b_1 b_3 c_3{}^2 - 12\beta^2 b_3 b_4 c_1 c_3 - 64\beta b_1 b_3 c_2 c_4 - 2b_3 b_4 c_1{}^2 - 40b_0{}^2 c_4 c_5 - 60\beta^2 b_0 b_3 c_3 c_5 \\
&-24\beta^2 b_0 b_4 c_1 c_6 - 4\beta b_3 b_5 c_1{}^2 - 8\beta b_3{}^2 c_2{}^2 - 12b_1{}^2 c_1 c_6 - 8\beta^2 b_1 b_6 c_2{}^2 - 2\beta^2 b_1 b_8 c_1{}^2 - 4\beta^2 b_4{}^2 c_1 c_2 \\
&-2b_0 b_7 c_1{}^2 - 84\beta b_0{}^2 c_3 c_7 - 36\beta b_0 b_4 c_3{}^2 - 144\beta b_0 b_1 c_3 c_6 - 16\beta b_2 b_5 c_1 c_2 - 40\beta^2 b_1{}^2 c_4 c_5 \\
&-48b_0 b_1 c_2 c_6 - 40\beta b_0 b_4 c_1 c_5 - 2\beta^2 b_2 b_7 c_1{}^2 - 8\beta^2 b_3{}^2 c_1 c_4 - 32\beta b_2 b_3 c_1 c_4 - 24\beta^2 b_2{}^2 c_3 c_4 \\
&-4\beta b_0 b_8 c_1{}^2 + 16Q^2\beta c_4{}^2 - 4\beta b_1 b_7 c_1{}^2 - 24b_1{}^2 c_3 c_4 - 16\beta b_0 b_7 c_1 c_2 - 2\beta^2 b_0 b_9 c_1{}^2 - 16\beta^2 b_1{}^2 c_1 c_8 \\
&-28\beta^2 b_1{}^2 c_2 c_7 - 36\beta^2 b_1{}^2 c_3 c_6 - 96\beta b_0 b_3 c_3 c_4 - 48\beta b_1 b_4 c_2 c_3 - 16\beta^2 b_1 b_5 c_1 c_4 - 160\beta b_0 b_1 c_4 c_5 \\
&-12\beta^2 b_1 b_6 c_1 c_3 - 8\beta^2 b_3 b_4 c_2{}^2 - 120\beta b_0 b_2 c_3 c_5 - 96\beta b_1 b_2 c_3 c_4 - 32\beta^2 b_0 b_3 c_4{}^2 - 18\beta^2 b_0 b_5 c_3{}^2 \\
&-8\beta^2 b_0 b_7 c_2{}^2 - 60\beta^2 b_1 b_2 c_3 c_5 - 64\beta b_0 b_4 c_2 c_4 - 50\beta b_0{}^2 c_5{}^2 - 36b_0{}^2 c_3 c_6 + 8Q^2\eta c_1 c_8 \\
&-12\beta^2 b_0 b_7 c_1 c_3 - 12\beta^2 b_3{}^2 c_2 c_3 - 8b_0 b_6 c_1 c_2 - 40b_0 b_2 c_2 c_5 - 80\beta b_0 b_3 c_2 c_5 - 8\beta^2 b_2 b_6 c_1 c_2 \\
&-2b_1 b_6 c_1{}^2 - 16\beta b_1 b_5 c_2{}^2 - 12b_2{}^2 c_2 c_3 + 3Q^2 c_1 c_6 - 18\beta b_2{}^2 c_3{}^2 - 32\beta^2 b_1 b_2 c_4{}^2 - 32\beta^2 b_0 b_5 c_2 c_4 \\
&-32\beta b_1 b_4 c_1 c_4 - 16\beta b_0 b_6 c_2{}^2 - 20\beta^2 b_2 b_3 c_1 c_5)
\end{aligned}
,
$$

where $\beta = (1+\alpha)/2$ and $\eta = (1+2\alpha-3\alpha^2)/4$.

S.3. An Example of Design and Numerical Calibration Based on Analytical Solutions

Assumed that a capacitive pressure sensor uses a circular conductive membrane with radius a = 100 mm, thickness h = 1 mm, Young's modulus of elasticity E = 7.84 MPa, Poisson's ratio ν = 0.47, and yield strength σ_y = 2.4 MPa. The insulator layer is assumed to take 0.1 mm of polystyrene, then t = 0.1 mm and ε_{r1} = 2.7. In addition, the vacuum permittivity $\varepsilon_0 = 8.854 \times 10^{-3}$ pF/mm, and the air relative permittivity ε_{r2} = 1.00053. The initial air parallel gap g takes 10 mm, 20 mm, 30 mm, 37 mm, respectively.

The total capacitance C of the capacitive pressure sensor under different pressure is determined by Equation (1) before the circular conductive membrane touches the insulator layer, and by Equation (7) after the circular conductive membrane touches the insulator layer. The power series expression for the deflection $w(r)$ in Equation (1) is determined by using the analytical solution given in reference [47], and that for the deflection $w(r)$ in Equation (7) is determined by using the analytical solution given in this Supplementary Materials S.1. The calculation results are listed in Tables S1 and S2 when g = 10 mm, in Tables S3 and S4 when g = 20 mm, in Tables S5 and S6 when g = 30 mm, and in Tables S7 and S8 when g = 37 mm.

Table S1. The calculation results of a circular capacitive pressure sensor operating in non-touch mode, where a = 100 mm, h = 1 mm, t = 0.1 mm, E = 7.84 MPa, ν = 0.47, and g = 10 mm.

q/KPa	w_m/mm	σ_m/MPa	C/pF
0	0.000	0.000	27.692
0.10	6.564	0.042	45.794
0.15	7.515	0.055	52.375
0.20	8.273	0.067	60.300

0.25	8.913	0.078	71.125
0.30	9.473	0.088	89.390
0.35	9.974	0.098	115.762
0.399	9.993	0.098	151.298

Table S2. The calculation results of a circular capacitive pressure sensor operating in touch mode, where a = 100 mm, h = 1 mm, t = 0.1 mm, E = 7.84 MPa, ν = 0.47, and g = 10 mm.

q/KPa	d/mm	b_0	b_1	c_0	σ_m/ MPa	C/pF
0.4	0.052	0.01351	−0.00210	0.07271	0.106	160.066
0.5	20.396	0.01395	−0.00248	0.07380	0.113	650.827
1	39.082	0.01885	−0.00261	0.07383	0.150	1533.881
5	65.422	0.03640	−0.00316	0.07536	0.287	3371.792
10	72.648	0.04896	−0.00355	0.07640	0.385	4028.670
20	78.224	0.06745	−0.00415	0.07779	0.530	4588.201
30	80.876	0.08254	−0.00480	0.07881	0.649	4872.141
40	82.538	0.09587	−0.00552	0.07965	0.753	5056.359
50	83.722	0.10804	−0.00631	0.08037	0.849	5190.674
60	84.628	0.11933	−0.00715	0.08102	0.938	5295.641
70	85.358	0.12992	−0.00803	0.08161	1.021	5381.536
80	85.966	0.13992	−0.00893	0.08215	1.100	5454.181
90	86.484	0.14942	−0.00985	0.08265	1.175	5517.197
100	86.936	0.15848	−0.01077	0.08313	1.246	5572.979
110	87.336	0.16715	−0.01171	0.08358	1.315	5623.225
120	87.694	0.17547	−0.01264	0.08401	1.380	5669.235
130	88.018	0.18347	−0.01358	0.08443	1.443	5712.097
140	88.312	0.19119	−0.01451	0.08482	1.504	5752.928
150	88.584	0.19864	−0.01544	0.08520	1.563	5793.245
160	88.834	0.20584	−0.01636	0.08557	1.619	5836.530
171	89.088	0.21351	−0.01737	0.08597	1.680	5880.785

Table S3. The calculation results of a circular capacitive pressure sensor operating in non-touch mode, where a = 100 mm, h = 1 mm, t = 0.1 mm, E = 7.84 MPa, ν = 0.47, and g = 20 mm.

q/KPa	w_m/mm	σ_m/MPa	C/pF
0	0.000	0.000	13.874
0.1	6.564	0.042	16.928
0.5	11.237	0.124	20.679
1.0	14.173	0.198	24.705
1.5	16.237	0.261	29.472
2.0	17.884	0.317	36.369
2.2	18.466	0.338	40.486
2.4	19.014	0.359	46.396
2.6	19.532	0.379	56.890
2.779	19.991	0.394	100.702

Table S4. The calculation results of a circular capacitive pressure sensor operating in touch mode, where a = 100 mm, h = 1 mm, t = 0.1 mm, E = 7.84 MPa, ν = 0.47, and g = 20 mm.

q/KPa	d/mm	b_0	b_1	c_0	σ_m/ MPa	C/pF
2.780	0.794	0.04857	−0.00817	0.15437	0.396	124.485
3	8.508	0.05026	−0.00876	0.15053	0.409	195.880
4	20.960	0.05758	−0.00927	0.14909	0.463	532.318
5	27.848	0.06400	−0.00936	0.14921	0.511	808.994
6	32.742	0.06971	−0.00938	0.14953	0.555	1039.723
7	36.518	0.07490	−0.00938	0.14990	0.595	1236.596
8	39.568	0.07970	−0.00938	0.15028	0.632	1407.265
9	42.106	0.08418	−0.00939	0.15065	0.666	1557.601
10	44.268	0.08841	−0.00940	0.15101	0.699	1691.536
15	51.726	0.10704	−0.00951	0.15264	0.844	2198.899
20	56.302	0.12307	−0.00968	0.15401	0.970	2546.781
30	61.918	0.15095	−0.01016	0.15627	1.189	3018.101
40	65.414	0.17540	−0.01084	0.15813	1.381	3344.773
50	67.900	0.19758	−0.01169	0.15974	1.556	3612.687
55	68.910	0.20799	−0.01216	0.16049	1.638	3735.351
57.65	69.400	0.21334	−0.01243	0.16087	1.680	3805.312

Table S5. The calculation results of a circular capacitive pressure sensor operating in non-touch mode, where a = 100 mm, h = 1 mm, t = 0.1 mm, E = 7.84 MPa, ν = 0.47, and g = 30 mm.

q/KPa	w_m/mm	σ_m/MPa	C/pF
0	0.000	0.000	9.255
0.1	6.564	0.042	10.489
0.3	9.473	0.088	11.198
0.5	11.237	0.124	11.698
1.0	14.173	0.198	12.686
2.0	17.884	0.317	14.352
3.0	20.496	0.419	15.984
4.0	22.579	0.511	17.759
5.0	24.342	0.596	19.826
6.0	25.884	0.677	22.401
7.0	27.264	0.755	25.916
8.0	28.519	0.829	31.568
8.5	29.108	0.865	36.493
9.0	29.674	0.901	46.739
9.2	29.895	0.916	58.646
9.25	29.949	0.919	66.467
9.29	29.993	0.922	79.656
9.293	29.999	0.922	89.325

Table S6. The calculation results of a circular capacitive pressure sensor operating in touch mode, where a = 100 mm, h = 1 mm, t = 0.1 mm, E = 7.84 MPa, ν = 0.47, and g = 30 mm.

q/KPa	d/mm	b_0	b_1	c_0	σ_m/ MPa	C/pF

9.294	1.854	0.11188	−0.01698	0.23288	0.924	97.515
9.4	4.626	0.11309	−0.01743	0.23056	0.930	111.892
9.5	5.596	0.11370	−0.01758	0.22996	0.933	122.263
10.0	9.186	0.11669	−0.01802	0.22839	0.955	175.823
12.5	19.112	0.13094	−0.01860	0.22717	1.051	430.828
15.0	25.142	0.14394	−0.01856	0.22766	1.149	656.294
17.5	29.592	0.15593	−0.01840	0.22842	1.241	855.083
20.0	33.114	0.16713	−0.01822	0.22925	1.327	1031.797
22.5	36.014	0.17770	−0.01806	0.23010	1.408	1191.072
25.0	38.466	0.18777	−0.01792	0.23093	1.486	1335.964
27.5	40.580	0.19741	−0.01781	0.23174	1.561	1470.357
30.0	42.432	0.20669	−0.01772	0.23252	1.634	1596.175
30.5	42.776	0.20851	−0.01771	0.23267	1.648	1620.956
31.0	43.112	0.21031	−0.01770	0.23283	1.662	1645.565
31.6	43.504	0.21246	−0.01768	0.23301	1.679	1674.881
31.64	43.530	0.21261	−0.01768	0.23302	1.680	1676.712

Table S7. The calculation results of a circular capacitive pressure sensor operating in non-touch mode, where a = 100 mm, h = 1 mm, t = 0.1 mm, E = 7.84 MPa, ν = 0.47, and g = 37 mm.

q/KPa	w_m/mm	σ_m/MPa	C/pF
0	0.000	0.000	7.504
0.1	6.564	0.042	8.289
1	14.173	0.198	9.552
3	20.496	0.419	11.143
5	24.342	0.596	12.576
7	27.264	0.755	14.107
10	30.746	0.972	16.925
13	33.587	1.173	21.201
15	35.244	1.302	26.335
17	36.760	1.426	43.287
17.332	36.999	1.446	90.792

Table S8. The calculation results of a circular capacitive pressure sensor operating in touch mode, where a = 100 mm, h = 1 mm, t = 0.1 mm, E = 7.84 MPa, ν = 0.47, and g = 37 mm.

q/KPa	d/mm	b_0	b_1	c_0	σ_m/ MPa	C/pF
17.333	3.140	0.17574	-0.02449	0.28691	1.447	110.091
18	7.972	0.17938	-0.02496	0.28530	1.449	147.723
19	11.110	0.18478	-0.02533	0.28429	1.489	206.103
20	13.600	0.19007	-0.02551	0.28390	1.529	264.187
21	15.712	0.19526	-0.02559	0.28380	1.568	321.486
22	17.568	0.20034	-0.02561	0.28384	1.606	377.856
23	19.230	0.20532	-0.02559	0.28398	1.643	433.448
23.99	20.726	0.21015	-0.02554	0.28417	1.680	487.552

S.4. Effect of Membrane Thickness on Capacitance–Pressure Relationships

In this section, the thickness of the circular conductive membrane is first increased from the reference thickness h = 1 mm to h = 1.5 mm, and then further to h = 2 mm. The initial air parallel gap g still takes 10 mm, 20 mm, 30 mm and 37 mm. The calculation results are listed in Tables S9–S16 when h = 1.5 mm, and in Tables S17–S24 when h = 2 mm. Figure S3 shows the input capacitance–output pressure relationships for g = 10 mm and h = 1 mm, 1.5 mm and 2 mm, Figure S4 for g = 20 mm and h = 1 mm, 1.5 mm and 2 mm, Figure S5 for g = 30 mm and h = 1 mm, 1.5 mm and 2 mm, and Figure S6 for g = 37 mm and h = 1 mm, 1.5 mm and 2 mm.

From Figures S3–S6 it can clearly be seen that increasing the thickness of the circular conductive membrane can increase the range of the output pressure q, but the range of the input capacitance C is almost unchanged. Therefore, increasing the thickness of the circular conductive membrane can also increase the output pressure per unit capacitance to some extent, because the range of the output pressure q increases while the range of the input capacitance C remains constant. For instance, as the thickness h increases from 1 mm to 1.5 mm and then to 2 mm, the output pressure per unit capacitance for g = 10 mm increases from 0.029 KPa/pF to 0.044KPa/pF and then to 0.058 KPa/pF, that for g = 20 mm increases from 0.015 KPa/pF to 0.023 KPa/pF and then to 0.030 KPa/pF, that for g = 30 mm increases from 0.019 KPa/pF to 0.028 KPa/pF and then to 0.038 KPa/pF, and that for g = 37 mm increases from 0.050 KPa/pF to 0.075 KPa/pF and then to 0.10 KPa/pF, which are calculated from Tables S1–S24 .

Table S9. The calculation results of a circular capacitive pressure sensor operating in non-touch mode, where a = 100 mm, h = 1.5 mm, t = 0.1 mm, E = 7.84 MPa, ν = 0.47, and g = 10 mm.

q/KPa	w_m/mm	σ_m/MPa	C/pF
0	0.000	0.000	27.692
0.15	6.564	0.042	45.794
0.225	7.515	0.055	52.375
0.3	8.273	0.067	60.300
0.375	8.913	0.078	71.125
0.45	9.473	0.088	89.390
0.525	9.974	0.098	115.762
0.5985	9.993	0.098	151.298

Table S10. The calculation results of a circular capacitive pressure sensor operating in touch mode, where a = 100 mm, h = 1.5 mm, t = 0.1 mm, E = 7.84 MPa, ν = 0.47, and g = 10 mm.

q/KPa	d/mm	b_0	b_1	c_0	σ_m/ MPa	C/pF
0.6	0.052	0.01351	−0.00210	0.07271	0.106	160.066
0.75	20.396	0.01395	−0.00248	0.07380	0.113	650.827
1.5	39.082	0.01885	−0.00261	0.07383	0.150	1533.881
7.5	65.422	0.03640	−0.00316	0.07536	0.287	3371.792
15	72.648	0.04896	−0.00355	0.07640	0.385	4028.670
30	78.224	0.06745	−0.00415	0.07779	0.530	4588.201
45	80.876	0.08254	−0.00480	0.07881	0.649	4872.141
60	82.538	0.09587	−0.00552	0.07965	0.753	5056.359
75	83.722	0.10804	−0.00631	0.08037	0.849	5190.674
90	84.628	0.11933	−0.00715	0.08102	0.938	5295.641
105	85.358	0.12992	−0.00803	0.08161	1.021	5381.536
120	85.966	0.13992	−0.00893	0.08215	1.100	5454.181

135	86.484	0.14942	−0.00985	0.08265	1.175	5517.197
150	86.936	0.15848	−0.01077	0.08313	1.246	5572.979
165	87.336	0.16715	−0.01171	0.08358	1.315	5623.225
180	87.694	0.17547	−0.01264	0.08401	1.380	5669.235
195	88.018	0.18347	−0.01358	0.08443	1.443	5712.097
210	88.312	0.19119	−0.01451	0.08482	1.504	5752.928
225	88.584	0.19864	−0.01544	0.08520	1.563	5793.245
240	88.834	0.20584	−0.01636	0.08557	1.619	5836.530
256.5	89.088	0.21351	−0.01737	0.08597	1.680	5880.785

Table S11. The calculation results of a circular capacitive pressure sensor operating in non-touch mode, where a = 100 mm, h = 1.5 mm, t = 0.1 mm, E = 7.84 MPa, ν = 0.47, and g = 20 mm.

q/KPa	w_m/mm	σ_m/MPa	C/pF
0	0	0	13.874
0.15	6.564	0.042	16.928
0.75	11.237	0.124	20.679
1.5	14.173	0.198	24.705
2.25	16.237	0.261	29.472
3	17.884	0.317	36.369
3.3	18.466	0.338	40.486
3.6	19.014	0.359	46.396
3.9	19.532	0.379	56.890
4.1685	19.991	0.394	100.702

Table S12. The calculation results of a circular capacitive pressure sensor operating in touch mode, where a = 100 mm, h = 1.5 mm, t = 0.1 mm, E = 7.84 MPa, ν = 0.47, and g = 20 mm.

q/KPa	d/mm	b_0	b_1	c_0	σ_m/ MPa	C/pF
4.17	0.794	0.04857	−0.00817	0.15437	0.396	124.485
4.5	8.508	0.05026	−0.00876	0.15053	0.409	195.880
6	20.960	0.05758	−0.00927	0.14909	0.463	532.318
7.5	27.848	0.064	−0.00936	0.14921	0.511	808.994
9	32.742	0.06971	−0.00938	0.14953	0.555	1039.723
10.5	36.518	0.0749	−0.00938	0.1499	0.595	1236.596
12	39.568	0.0797	−0.00938	0.15028	0.632	1407.265
13.5	42.106	0.08418	−0.00939	0.15065	0.666	1557.601
15	44.268	0.08841	−0.00940	0.15101	0.699	1691.536
22.5	51.726	0.10704	−0.00951	0.15264	0.844	2198.899
30	56.302	0.12307	−0.00968	0.15401	0.970	2546.781
45	61.918	0.15095	−0.01016	0.15627	1.189	3018.101
60	65.414	0.1754	−0.01084	0.15813	1.381	3344.773
75	67.900	0.19758	−0.01169	0.15974	1.556	3612.687
82.5	68.910	0.20799	−0.01216	0.16049	1.638	3735.351
86.475	69.400	0.21334	−0.01243	0.16087	1.680	3805.312

Table S13. The calculation results of a circular capacitive pressure sensor operating in non-touch mode, where a = 100 mm, h = 1.5 mm, t = 0.1 mm, E = 7.84 MPa, ν = 0.47, and g = 30 mm.

q/KPa	w_m/mm	σ_m/MPa	C/pF
0	0	0	9.255
0.15	6.564	0.042	10.489
0.45	9.473	0.088	11.198
0.75	11.237	0.124	11.698
1.5	14.173	0.198	12.686
3	17.884	0.317	14.352
4.5	20.496	0.419	15.984
6	22.579	0.511	17.759
7.5	24.342	0.596	19.826
9	25.884	0.677	22.401
10.5	27.264	0.755	25.916
12	28.519	0.829	31.568
12.75	29.108	0.865	36.493
13.5	29.674	0.901	46.739
13.8	29.895	0.916	58.646
13.875	29.949	0.919	66.467
13.935	29.993	0.922	79.656
13.9395	29.999	0.922	89.325

Table S14. The calculation results of a circular capacitive pressure sensor operating in touch mode, where a = 100 mm, h = 1.5 mm, t = 0.1 mm, E = 7.84 MPa, ν = 0.47, and g = 30 mm.

q/KPa	d/mm	b_0	b_1	c_0	σ_m/ MPa	C/pF
13.941	1.854	0.11188	−0.01698	0.23288	0.924	97.515
14.1	4.626	0.11309	−0.01743	0.23056	0.93	111.892
14.25	5.596	0.1137	−0.01758	0.22996	0.933	122.263
15	9.186	0.11669	−0.01802	0.22839	0.955	175.823
18.75	19.112	0.13094	−0.01860	0.22717	1.051	430.828
22.5	25.142	0.14394	−0.01856	0.22766	1.149	656.294
26.25	29.592	0.15593	−0.01840	0.22842	1.241	855.083
30	33.114	0.16713	−0.01822	0.22925	1.327	1031.797
33.75	36.014	0.1777	−0.01806	0.2301	1.408	1191.072
37.5	38.466	0.18777	−0.01792	0.23093	1.486	1335.964
41.25	40.580	0.19741	−0.01781	0.23174	1.561	1470.357
45	42.432	0.20669	−0.01772	0.23252	1.634	1596.175
45.75	42.776	0.20851	−0.01771	0.23267	1.648	1620.956
46.5	43.112	0.21031	−0.01770	0.23283	1.662	1645.565
47.4	43.504	0.21246	−0.01768	0.23301	1.679	1674.881
47.46	43.530	0.21261	−0.01768	0.23302	1.68	1676.712

Table S15. The calculation results of a circular capacitive pressure sensor operating in non-touch mode, where a = 100 mm, h = 1.5 mm, t = 0.1 mm, E = 7.84 MPa, ν = 0.47, and g = 37 mm.

q/KPa	w_m/mm	σ_m/MPa	C/pF

0	0.000	0.000	7.504
0.15	6.564	0.042	8.289
1.5	14.173	0.198	9.552
4.5	20.496	0.419	11.143
7.5	24.342	0.596	12.576
10.5	27.264	0.755	14.107
15	30.746	0.972	16.925
19.5	33.587	1.173	21.201
22.5	35.244	1.302	26.335
25.5	36.760	1.426	43.287
25.998	36.999	1.446	90.792

Table S16. The calculation results of a circular capacitive pressure sensor operating in touch mode, where a = 100 mm, h = 1.5 mm, t = 0.1 mm, E = 7.84 MPa, ν = 0.47, and g = 37 mm.

q/KPa	d/mm	b_0	b_1	c_0	σ_m/ MPa	C/pF
25.9995	3.140	0.17574	-0.02449	0.28691	1.447	110.091
27	7.972	0.17938	-0.02496	0.28530	1.449	147.723
28.5	11.110	0.18478	-0.02533	0.28429	1.489	206.103
30	13.600	0.19007	-0.02551	0.28390	1.529	264.187
31.5	15.712	0.19526	-0.02559	0.28380	1.568	321.486
33	17.568	0.20034	-0.02561	0.28384	1.606	377.856
34.5	19.230	0.20532	-0.02559	0.28398	1.643	433.448
35.985	20.726	0.21015	-0.02554	0.28417	1.680	487.552

Table S17. The calculation results of a circular capacitive pressure sensor operating in non-touch mode, where a = 100 mm, h = 2 mm, t = 0.1 mm, E = 7.84 MPa, ν = 0.47, and g = 10 mm.

q/KPa	w_m/mm	σ_m/MPa	C/pF
0	0.000	0.000	27.692
0.2	6.564	0.042	45.794
0.3	7.515	0.055	52.375
0.4	8.273	0.067	60.300
0.5	8.913	0.078	71.125
0.6	9.473	0.088	89.390
0.7	9.974	0.098	115.762
0.798	9.993	0.098	151.298

Table S18. The calculation results of a circular capacitive pressure sensor operating in touch mode, where a = 100 mm, h = 2 mm, t = 0.1 mm, E = 7.84 MPa, ν = 0.47, and g = 10 mm.

q/KPa	d/mm	b_0	b_1	c_0	σ_m/ MPa	C/pF
0.8	0.052	0.01351	−0.00210	0.07271	0.106	160.066
1	20.396	0.01395	−0.00248	0.07380	0.113	650.827
2	39.082	0.01885	−0.00261	0.07383	0.150	1533.881
10	65.422	0.03640	−0.00316	0.07536	0.287	3371.792
20	72.648	0.04896	−0.00355	0.07640	0.385	4028.670
40	78.224	0.06745	−0.00415	0.07779	0.530	4588.201

60	80.876	0.08254	−0.00480	0.07881	0.649	4872.141
80	82.538	0.09587	−0.00552	0.07965	0.753	5056.359
100	83.722	0.10804	−0.00631	0.08037	0.849	5190.674
120	84.628	0.11933	−0.00715	0.08102	0.938	5295.641
140	85.358	0.12992	−0.00803	0.08161	1.021	5381.536
160	85.966	0.13992	−0.00893	0.08215	1.100	5454.181
180	86.484	0.14942	−0.00985	0.08265	1.175	5517.197
200	86.936	0.15848	−0.01077	0.08313	1.246	5572.979
220	87.336	0.16715	−0.01171	0.08358	1.315	5623.225
240	87.694	0.17547	−0.01264	0.08401	1.380	5669.235
260	88.018	0.18347	−0.01358	0.08443	1.443	5712.097
280	88.312	0.19119	−0.01451	0.08482	1.504	5752.928
300	88.584	0.19864	−0.01544	0.08520	1.563	5793.245
320	88.834	0.20584	−0.01636	0.08557	1.619	5836.530
342	89.088	0.21351	−0.01737	0.08597	1.680	5880.785

Table S19. The calculation results of a circular capacitive pressure sensor operating in non-touch mode, where a = 100 mm, h = 2 mm, t = 0.1 mm, E = 7.84 MPa, ν = 0.47, and g = 20 mm.

q/KPa	w_m/mm	σ_m/MPa	C/pF
0	0	0	13.874
0.2	6.564	0.042	16.928
1	11.237	0.124	20.679
2	14.173	0.198	24.705
3	16.237	0.261	29.472
4	17.884	0.317	36.369
4.4	18.466	0.338	40.486
4.8	19.014	0.359	46.396
5.2	19.532	0.379	56.890
5.558	19.991	0.394	100.702

Table S20. The calculation results of a circular capacitive pressure sensor operating in touch mode, where a = 100 mm, h = 2 mm, t = 0.1 mm, E = 7.84 MPa, ν = 0.47, and g = 20 mm.

q/KPa	d/mm	b_0	b_1	c_0	σ_m/ MPa	C/pF
5.56	0.794	0.04857	−0.00817	0.15437	0.396	124.485
6	8.508	0.05026	−0.00876	0.15053	0.409	195.880
8	20.960	0.05758	−0.00927	0.14909	0.463	532.318
10	27.848	0.064	−0.00936	0.14921	0.511	808.994
12	32.742	0.06971	−0.00938	0.14953	0.555	1039.723
14	36.518	0.0749	−0.00938	0.1499	0.595	1236.596
16	39.568	0.0797	−0.00938	0.15028	0.632	1407.265
18	42.106	0.08418	−0.00939	0.15065	0.666	1557.601
20	44.268	0.08841	−0.00940	0.15101	0.699	1691.536
30	51.726	0.10704	−0.00951	0.15264	0.844	2198.899
40	56.302	0.12307	−0.00968	0.15401	0.970	2546.781

60	61.918	0.15095	−0.01016	0.15627	1.189	3018.101
80	65.414	0.1754	−0.01084	0.15813	1.381	3344.773
100	67.900	0.19758	−0.01169	0.15974	1.556	3612.687
110	68.910	0.20799	−0.01216	0.16049	1.638	3735.351
115.3	69.400	0.21334	−0.01243	0.16087	1.680	3805.312

Table S21. The calculation results of a circular capacitive pressure sensor operating in non-touch mode, where a = 100 mm, h = 2 mm, t = 0.1 mm, E = 7.84 MPa, ν = 0.47, and g = 30 mm.

q/KPa	w_m/mm	σ_m/MPa	C/pF
0	0	0	9.255
0.2	6.564	0.042	10.489
0.6	9.473	0.088	11.198
1	11.237	0.124	11.698
2	14.173	0.198	12.686
4	17.884	0.317	14.352
6	20.496	0.419	15.984
8	22.579	0.511	17.759
10	24.342	0.596	19.826
12	25.884	0.677	22.401
14	27.264	0.755	25.916
16	28.519	0.829	31.568
17	29.108	0.865	36.493
18	29.674	0.901	46.739
18.4	29.895	0.916	58.646
18.5	29.949	0.919	66.467
18.58	29.993	0.922	79.656
18.586	29.999	0.922	89.325

Table S22. The calculation results of a circular capacitive pressure sensor operating in touch mode, where a = 100 mm, h = 2 mm, t = 0.1 mm, E = 7.84 MPa, ν = 0.47, and g = 30 mm.

q/KPa	d/mm	b_0	b_1	c_0	σ_m/ MPa	C/pF
18.588	1.854	0.11188	−0.01698	0.23288	0.924	97.515
18.8	4.626	0.11309	−0.01743	0.23056	0.930	111.892
19	5.596	0.1137	−0.01758	0.22996	0.933	122.263
20	9.186	0.11669	−0.01802	0.22839	0.955	175.823
25	19.112	0.13094	−0.01860	0.22717	1.051	430.828
30	25.142	0.14394	−0.01856	0.22766	1.149	656.294
35	29.592	0.15593	−0.01840	0.22842	1.241	855.083
40	33.114	0.16713	−0.01822	0.22925	1.327	1031.797
45	36.014	0.1777	−0.01806	0.2301	1.408	1191.072
50	38.466	0.18777	−0.01792	0.23093	1.486	1335.964
55	40.580	0.19741	−0.01781	0.23174	1.561	1470.357
60	42.432	0.20669	−0.01772	0.23252	1.634	1596.175
61	42.776	0.20851	−0.01771	0.23267	1.648	1620.956

62	43.112	0.21031	−0.01770	0.23283	1.662	1645.565
63.2	43.504	0.21246	−0.01768	0.23301	1.679	1674.881
63.28	43.530	0.21261	−0.01768	0.23302	1.680	1676.712

Table S23. The calculation results of a circular capacitive pressure sensor operating in non-touch mode, where a = 100 mm, h = 2 mm, t = 0.1 mm, E = 7.84 MPa, ν = 0.47, and g = 37 mm.

q/KPa	w_m/mm	σ_m/MPa	C/pF
0	0.000	0.000	7.504
0.2	6.564	0.042	8.289
2	14.173	0.198	9.552
6	20.496	0.419	11.143
10	24.342	0.596	12.576
14	27.264	0.755	14.107
20	30.746	0.972	16.925
26	33.587	1.173	21.201
30	35.244	1.302	26.335
34	36.760	1.426	43.287
34.664	36.999	1.446	90.792

Table S24. The calculation results of a circular capacitive pressure sensor operating in touch mode, where a = 100 mm, h = 2 mm, t = 0.1 mm, E = 7.84 MPa, ν = 0.47, and g = 37 mm.

q/KPa	d/mm	b_0	b_1	c_0	σ_m/ MPa	C/pF
34.666	3.140	0.17574	-0.02449	0.28691	1.447	110.091
36	7.972	0.17938	-0.02496	0.28530	1.449	147.723
38	11.110	0.18478	-0.02533	0.28429	1.489	206.103
40	13.600	0.19007	-0.02551	0.28390	1.529	264.187
42	15.712	0.19526	-0.02559	0.28380	1.568	321.486
44	17.568	0.20034	-0.02561	0.28384	1.606	377.856
46	19.230	0.20532	-0.02559	0.28398	1.643	433.448
47.98	20.726	0.21015	-0.02554	0.28417	1.680	487.552

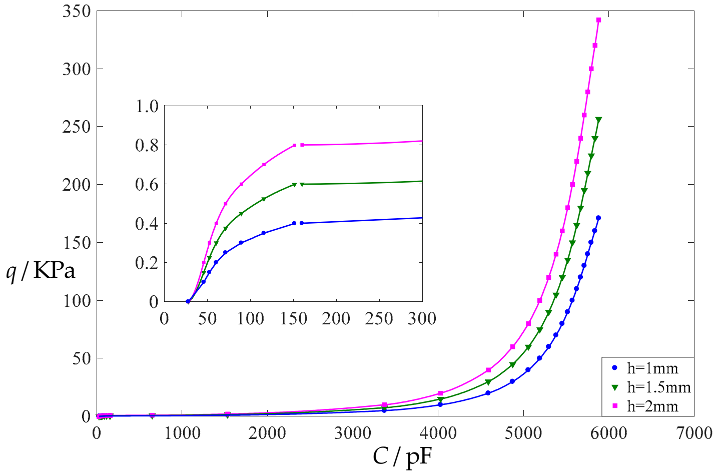

Figure S3. The capacitance-pressure relationships of a circular capacitive pressure sensor from non-touch mode of operation to touch mode of operation, where a = 100 mm, h = 1 mm,1.5 mm and 2 mm, t = 0.1 mm, E = 7.84 MPa, ν = 0.47, and g = 10 mm.

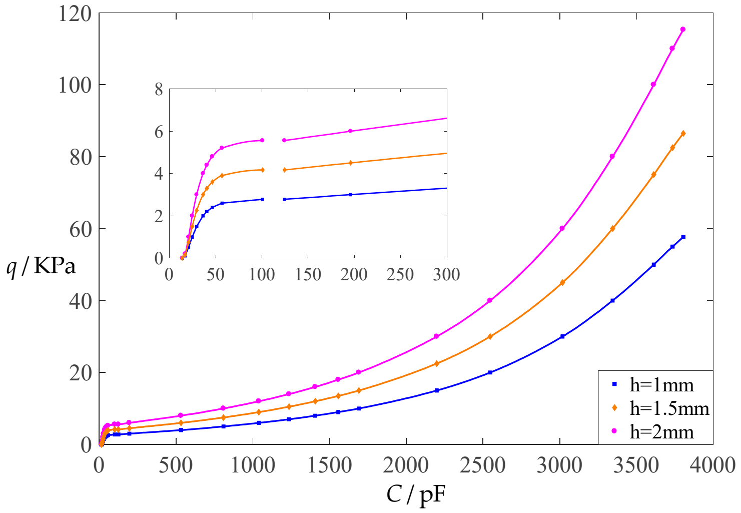

Figure S4. The capacitance-pressure relationships of a circular capacitive pressure sensor from non-touch mode of operation to touch mode of operation, where $a = 100$ mm, $h = 1$ mm, 1.5 mm and 2 mm, $t = 0.1$ mm, $E = 7.84$ MPa, $\nu = 0.47$, and $g = 20$ mm.

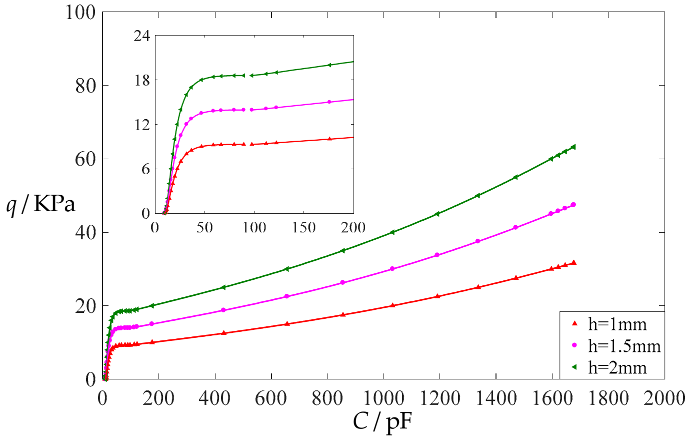

Figure S5. The capacitance-pressure relationships of a circular capacitive pressure sensor from non-touch mode of operation to touch mode of operation, where $a = 100$ mm, $h = 1$ mm, 1.5 mm and 2 mm, $t = 0.1$ mm, $E = 7.84$ MPa, $\nu = 0.47$, and $g = 30$ mm.

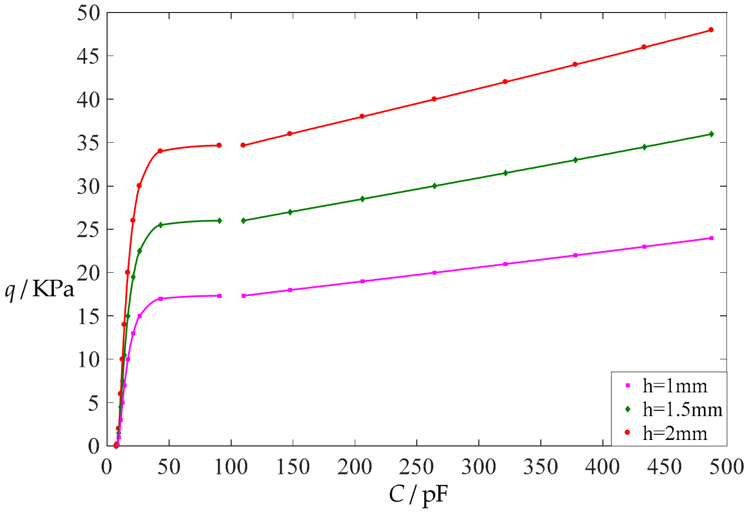

Figure S6. The capacitance-pressure relationships of a circular capacitive pressure sensor from non-touch mode of operation to touch mode of operation, where $a = 100$ mm, $h = 1$ mm, 1.5 mm and 2 mm, $t = 0.1$ mm, $E = 7.84$ MPa, $\nu = 0.47$, and $g = 37$ mm.

S.5. Effect of Young's Modulus of Elasticity on Capacitance–Pressure Relationships

In this section, the Young's modulus of elasticity E of the circular conductive membrane is first decreased from the reference value E = 7.84 MPa to E = 5 MPa, and then further to E = 2.5 MPa. The initial air parallel gap g still takes 10 mm, 20 mm, 30 mm and 37 mm. The calculation results are listed in Tables S25–S32 when E = 5 MPa, and in Tables S33–S40 when E = 2.5 MPa. Figure S7 shows the input capacitance–output pressure relationships for g = 10 mm and E = 7.84 MPa, 5 MPa and 2.5 MPa, Figure S8 for g = 20 mm and E = 7.84 MPa, 5 MPa and 2.5 MPa, Figure S9 for g = 30 mm and E = 7.84 MPa, 5 MPa and 2.5 MPa, and Figure S10 for g = 37 mm and E = 7.84 MPa, 5 MPa and 2.5 MPa.

From Figures S7–S10 it can clearly be seen that decreasing the Young's modulus of elasticity E of the circular conductive membrane can increase both the range of the output pressure q and the range of the input capacitance C. Therefore, the effect of decreasing the Young's modulus of elasticity E on the output pressure per unit capacitance is uncertain. For instance, as the Young's modulus of elasticity E decreases from 7.84 MPa to 5 MPa and then to 2.5 MPa, the output pressure per unit capacitance for g = 10 mm increases from 0.029 KPa/pF to 0.041 KPa/pF and then to 0.082 KPa/pF, that for g = 20 mm increases from 0.015 KPa/pF to 0.018 KPa/pF and then to 0.030 KPa/pF, that for g = 30 mm first decreases from 0.019 KPa/pF to 0.016 KPa/pF and then increases to 0.019 KPa/pF, and that for g = 37 mm decreases from 0.050 KPa/pF to 0.021 KPa/pF and then to 0.16 KPa/pF, which are calculated from Tables S1–S8 and S25–S40.

Table S25. The calculation results of a circular capacitive pressure sensor operating in non-touch mode, where a = 100 mm, h = 1 mm, t = 0.1 mm, E = 5 MPa, ν = 0.47, and g = 10 mm.

q/KPa	w_m/mm	σ_m/MPa	C/pF
0	0.000	0.000	27.692
0.01	3.538	0.008	34.402
0.03	5.103	0.016	39.165
0.05	6.052	0.023	43.122
0.1	7.627	0.036	53.350
0.15	8.733	0.048	67.462
0.2	9.614	0.058	97.671
0.224	9.986	0.062	190.966

Table S26. The calculation results of a circular capacitive pressure sensor operating in touch mode, where a = 100 mm, h = 1 mm, t = 0.1 mm, E = 5 MPa, ν = 0.47, and g = 10 mm.

q/KPa	d/mm	b_0	b_1	c_0	σ_m/ MPa	C/pF
0.225	0.802	0.01197	-0.00216	0.07666	0.063	204.605
0.23	4.248	0.01208	-0.00224	0.07551	0.063	221.935
0.25	10.226	0.01252	-0.00235	0.07445	0.065	322.252
0.3	18.224	0.01357	-0.00246	0.07387	0.070	544.414
0.5	33.352	0.01700	-0.00257	0.07372	0.086	1181.280
1	48.192	0.02269	-0.00271	0.07414	0.114	2060.232
5	70.322	0.04403	-0.00340	0.07600	0.221	3887.385
10	76.428	0.06007	-0.00390	0.07725	0.301	4511.028
20	81.144	0.08444	-0.00489	0.07893	0.423	5030.404
50	85.874	0.13835	-0.00878	0.08206	0.694	5585.772
100	88.756	0.20356	-0.01607	0.08546	1.021	5940.881
150	90.274	0.25368	-0.02304	0.08805	1.273	6133.083

180	90.926	0.27920	-0.02696	0.08941	1.401	6216.609
200	91.294	0.29478	-0.02945	0.09026	1.479	6264.084
220	91.622	0.30938	-0.03186	0.09106	1.552	6306.503
250	92.056	0.32969	-0.03532	0.09221	1.654	6362.597
258	92.160	0.33482	-0.03622	0.09250	1.680	6376.278

Table S27. The calculation results of a circular capacitive pressure sensor operating in non-touch mode, where a = 100 mm, h = 1 mm, t = 0.1 mm, E = 5 MPa, ν = 0.47, and g = 20 mm.

q/KPa	w_m/mm	σ_m/MPa	C/pF
0	0.000	0.000	13.874
0.1	7.627	0.036	17.613
0.3	11.010	0.076	20.441
0.5	13.063	0.107	22.933
0.7	14.622	0.134	25.546
1	16.481	0.171	30.239
1.3	17.998	0.205	37.070
1.5	18.885	0.226	44.730
1.6	19.299	0.236	51.134
1.7	19.697	0.246	63.194
1.75	19.890	0.251	78.293
1.779	20.000	0.253	108.545

Table S28. The calculation results of a circular capacitive pressure sensor operating in touch mode, where a = 100 mm, h = 1 mm, t = 0.1 mm, E = 5 MPa, ν = 0.47, and g = 20 mm.

q/KPa	d/mm	b_0	b_1	c_0	σ_m/ MPa	C/pF
1.78	1.770	0.04865	-0.00826	0.15361	0.254	116.725
2	11.044	0.05131	-0.00891	0.14996	0.266	249.068
5	39.118	0.07895	-0.00938	0.15022	0.399	1389.495
10	52.468	0.10934	-0.00953	0.15284	0.550	2285.940
20	62.480	0.15444	-0.01024	0.15654	0.776	3115.739
30	67.238	0.19121	-0.01142	0.15928	0.960	3560.097
40	70.258	0.22331	-0.01296	0.16157	1.122	3860.599
50	72.446	0.25208	-0.01476	0.16358	1.266	4088.533
60	74.150	0.27826	-0.01672	0.16540	1.398	4274.411
70	75.544	0.30232	-0.01879	0.16710	1.520	4436.418
80	76.718	0.32460	-0.02092	0.16869	1.632	4590.293
84.5	77.192	0.33411	-0.02189	0.16938	1.680	4658.715

Table S29. The calculation results of a circular capacitive pressure sensor operating in non-touch mode, where a = 100 mm, h = 1 mm, t = 0.1 mm, E = 5 MPa, ν = 0.47, and g = 30 mm.

q/KPa	w_m/mm	σ_m/MPa	C/pF
0	0.000	0.000	9.255
0.2	9.614	0.058	11.236
0.5	13.063	0.107	12.286
1	16.481	0.171	13.652

2	20.804	0.275	16.214
3	23.846	0.364	19.173
4	26.274	0.445	23.236
5	28.326	0.521	30.407
5.5	29.250	0.558	38.225
5.8	29.779	0.579	50.782
5.89	29.995	0.580	66.748

Table S30. The calculation results of a circular capacitive pressure sensor operating in touch mode, where a = 100 mm, h = 1 mm, t = 0.1 mm, E = 5 MPa, ν = 0.47, and g = 30 mm.

q/KPa	d/mm	b_0	b_1	c_0	σ_m/ MPa	C/pF
5.9	2.762	0.11219	-0.01714	0.23201	0.581	96.252
6	4.708	0.11314	-0.01745	0.23051	0.586	114.869
10	26.472	0.14729	-0.01852	0.22785	0.749	719.988
15	37.062	0.18187	-0.01800	0.23044	0.919	1246.977
20	43.348	0.21160	-0.01769	0.23293	1.066	1631.139
25	47.708	0.23840	-0.01763	0.23519	1.200	1930.742
30	50.998	0.26310	-0.01778	0.23725	1.324	2176.752
35	53.618	0.28614	-0.01811	0.23915	1.439	2387.671
40	55.786	0.30780	-0.01859	0.24093	1.548	2577.525
46.4	58.100	0.33381	-0.01939	0.24307	1.679	2838.314
46.45	58.118	0.33400	-0.01940	0.24308	1.680	2841.388

Table S31. The calculation results of a circular capacitive pressure sensor operating in non-touch mode, where a = 100 mm, h = 1 mm, t = 0.1 mm, E = 5 MPa, ν = 0.47, and g = 37 mm.

q/KPa	w_m/mm	σ_m/MPa	C/pF
0	0.000	0.000	7.504
0.1	7.627	0.036	8.438
0.5	13.063	0.107	9.335
1	16.481	0.171	10.055
3	23.846	0.364	12.361
5	28.326	0.521	14.814
7	31.726	0.662	18.083
9	34.528	0.794	23.642
10	35.774	0.857	29.255
11	36.939	0.919	55.575
11.053	36.999	0.923	91.811

Table S32. The calculation results of a circular capacitive pressure sensor operating in touch mode, where a = 100 mm, h = 1 mm, t = 0.1 mm, E = 5 MPa, ν = 0.47, and g = 37 mm.

q/KPa	d/mm	b_0	b_1	c_0	σ_m/ MPa	C/pF
11.054	5.102	0.17570	-0.02448	0.28694	0.926	111.714
12	10.594	0.18380	-0.02528	0.28441	0.945	203.020
13	14.448	0.19208	-0.02555	0.28384	0.985	294.598
15	20.032	0.20787	-0.02556	0.28407	1.060	465.302

17	24.204	0.22275	-0.02533	0.28486	1.132	621.535
20	29.032	0.24367	-0.02487	0.28631	1.235	832.798
23	32.802	0.26325	-0.02443	0.28784	1.331	1021.066
25	34.920	0.27570	-0.02418	0.28886	1.393	1136.352
30	39.266	0.30506	-0.02372	0.29134	1.539	1398.010
35	42.686	0.33239	-0.02349	0.29369	1.675	1641.374
35.2	42.808	0.33345	-0.02349	0.29378	1.680	1651.509

Table S33. The calculation results of a circular capacitive pressure sensor operating in non-touch mode, where a = 100 mm, h = 1 mm, t = 0.1 mm, E = 2.5 MPa, ν = 0.47, and g = 10 mm.

q/KPa	w_m/mm	σ_m/MPa	C/pF
0	0.000	0.000	27.692
0.005	3.538	0.004	34.402
0.01	4.458	0.006	36.991
0.03	6.431	0.013	45.058
0.05	7.627	0.018	53.350
0.06	8.106	0.021	58.240
0.07	8.534	0.023	64.047
0.08	8.923	0.025	71.359
0.09	9.282	0.027	81.403
0.1	9.614	0.029	97.671
0.11	9.926	0.031	143.624
0.1123	9.994	0.031	183.810

Table S34. The calculation results of a circular capacitive pressure sensor operating in touch mode, where a = 100 mm, h = 1 mm, t = 0.1 mm, E = 2.5 MPa, ν = 0.47, and g = 10 mm.

q/KPa	d/mm	b_0	b_1	c_0	σ_m/ MPa	C/pF
0.113	1.864	0.01200	-0.00218	0.07624	0.032	206.007
0.5	48.192	0.02269	-0.00271	0.07414	0.057	2258.342
1	59.390	0.03006	-0.00295	0.07480	0.076	3183.107
5	76.428	0.06007	-0.00390	0.07725	0.151	4925.238
10	81.144	0.08444	-0.00489	0.07893	0.212	5480.927
20	84.842	0.12227	-0.00738	0.08118	0.307	5939.478
30	86.676	0.15316	-0.01022	0.08285	0.384	6174.429
50	88.756	0.20356	-0.01607	0.08546	0.511	6446.820
70	90.022	0.24450	-0.02169	0.08757	0.613	6605.947
100	91.294	0.29478	-0.02945	0.09026	0.740	6728.208
150	92.656	0.36005	-0.04071	0.09397	0.903	6855.519
200	93.404	0.40163	-0.04847	0.09651	1.007	6921.600
250	94.030	0.43152	-0.05384	0.09906	1.122	6944.498
568	98.136	0.56472	-0.07086	0.12437	1.680	6952.120

Table S35. The calculation results of a circular capacitive pressure sensor operating in non-touch mode, where a = 100 mm, h = 1 mm, t = 0.1 mm, E = 2.5 MPa, ν = 0.47, and g = 20 mm.

q/KPa	w_m/mm	σ_m/MPa	C/pF

0	0.000	0.000	13.874
0.01	4.458	0.006	15.762
0.05	7.627	0.018	17.613
0.1	9.614	0.029	19.130
0.3	13.886	0.061	24.209
0.5	16.481	0.086	30.239
0.7	18.452	0.108	40.372
0.8	19.299	0.118	51.134
0.85	19.697	0.123	63.194
0.87	19.851	0.125	73.803
0.889	19.996	0.126	129.696

Table S36. The calculation results of a circular capacitive pressure sensor operating in touch mode, where a = 100 mm, h = 1 mm, t = 0.1 mm, E = 2.5 MPa, ν = 0.47, and g = 20 mm.

q/KPa	d/mm	b_0	b_1	c_0	σ_m/ MPa	C/pF
0.89	1.770	0.04865	-0.00826	0.15361	0.127	135.408
0.9	3.468	0.04889	-0.00840	0.15253	0.127	156.983
1	11.044	0.05131	-0.00891	0.14996	0.133	311.516
5	52.468	0.10934	-0.00953	0.15284	0.275	2551.112
10	62.480	0.15444	-0.01024	0.15654	0.388	3442.698
20	70.258	0.22331	-0.01296	0.16157	0.561	4231.557
30	74.150	0.27826	-0.01672	0.16540	0.699	4658.056
50	78.626	0.36476	-0.02526	0.17164	0.917	5174.847
70	81.378	0.43187	-0.03386	0.17688	1.086	5506.541
90	83.338	0.48653	-0.04200	0.18153	1.224	5749.120
100	84.134	0.51042	-0.04586	0.18369	1.284	5849.103
130	86.040	0.57198	-0.05658	0.18962	1.438	6092.211
150	87.032	0.60653	-0.06307	0.19320	1.525	6220.484
170	87.868	0.63718	-0.06909	0.19655	1.602	6329.697
190	88.586	0.66467	-0.07470	0.19969	1.671	6424.178
193	88.684	0.66855	-0.07551	0.20015	1.681	6437.269

Table S37. The calculation results of a circular capacitive pressure sensor operating in non-touch mode, where a = 100 mm, h = 1 mm, t = 0.1 mm, E = 2.5 MPa, ν = 0.47, and g = 30 mm.

q/KPa	w_m/mm	σ_m/MPa	C/pF
0	0.000	0.000	9.255
0.1	9.614	0.029	11.236
0.5	16.481	0.086	13.652
1	20.804	0.138	16.214
1.5	23.846	0.182	19.173
2	26.274	0.223	23.236
2.4	27.939	0.253	28.473
2.7	29.070	0.275	36.081
2.9	29.779	0.286	50.782

2.95	29.951	0.288	66.748
2.964	29.999	0.289	104.366

Table S38. The calculation results of a circular capacitive pressure sensor operating in touch mode, where a = 100 mm, h = 1 mm, t = 0.1 mm, E = 2.5 MPa, ν = 0.47, and g = 30 mm.

q/KPa	d/mm	b_0	b_1	c_0	σ_m/ MPa	C/pF
2.965	3.436	0.11248	-0.01725	0.23144	0.291	134.910
2.97	3.638	0.11257	-0.01728	0.23128	0.292	137.881
5	26.472	0.14729	-0.01852	0.22785	0.375	855.448
10	43.348	0.21160	-0.01769	0.23293	0.533	1844.731
20	55.786	0.30780	-0.01859	0.24093	0.774	2823.887
30	61.918	0.38379	-0.02165	0.24723	0.966	3385.950
40	65.964	0.44710	-0.02587	0.25272	1.125	3786.025
50	68.972	0.50120	-0.03068	0.25773	1.262	4098.880
60	71.354	0.54825	-0.03576	0.26239	1.381	4356.314
70	73.312	0.58972	-0.04091	0.26676	1.486	4575.191
80	74.968	0.62668	-0.04603	0.27091	1.579	4766.437
85	75.704	0.64372	-0.04855	0.27290	1.622	4854.925
90	76.390	0.65992	-0.05105	0.27485	1.663	4942.695
92	76.652	0.66618	-0.05203	0.27561	1.679	4982.083
92.2	76.678	0.66680	-0.05213	0.27569	1.680	4986.705

Table S39. The calculation results of a circular capacitive pressure sensor operating in non-touch mode, where a = 100 mm, h = 1 mm, t = 0.1 mm, E = 2.5 MPa, ν = 0.47, and g = 37 mm.

q/KPa	w_m/mm	σ_m/MPa	C/pF
0	0.000	0.000	7.504
0.1	9.614	0.029	8.737
0.5	16.481	0.086	10.055
1	20.804	0.138	11.241
3	30.121	0.297	16.294
5	35.774	0.429	29.255
5.5	36.939	0.460	55.575
5.526	36.998	0.461	85.442

Table S40. The calculation results of a circular capacitive pressure sensor operating in touch mode, where a = 100 mm, h = 1 mm, t = 0.1 mm, E = 2.5 MPa, ν = 0.47, and g = 37 mm.

q/KPa	d/mm	b_0	b_1	c_0	σ_m/ MPa	C/pF
5.527	5.102	0.17570	-0.02448	0.28694	0.463	124.189
6	10.594	0.18380	-0.02528	0.28441	0.473	271.612
10	29.032	0.24367	-0.02487	0.28631	0.617	974.853
20	45.492	0.35805	-0.02348	0.29593	0.902	1996.925
30	53.352	0.44788	-0.02512	0.30398	1.128	2618.337
40	58.502	0.52243	-0.02861	0.31112	1.316	3074.940
50	62.324	0.58576	-0.03318	0.31769	1.476	3445.600
60	65.348	0.64044	-0.03835	0.32384	1.614	3801.335

65	66.646	0.66511	-0.04106	0.32679	1.677	3958.460
65.2	66.696	0.66607	-0.04117	0.32691	1.680	3967.224

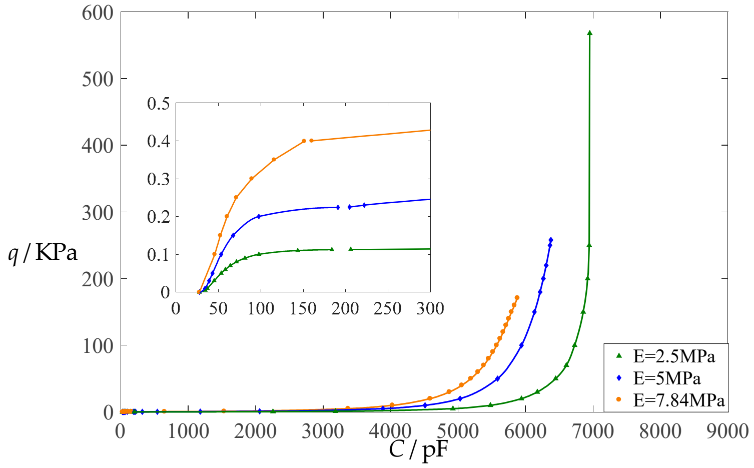

Figure S7. The capacitance-pressure relationships of a circular capacitive pressure sensor from non-touch mode of operation to touch mode of operation, where a = 100 mm, h = 1 mm, t = 0.1 mm, E = 7.84 MPa, 5 MPa and 2.5 MPa, ν = 0.47, and g = 10 mm.

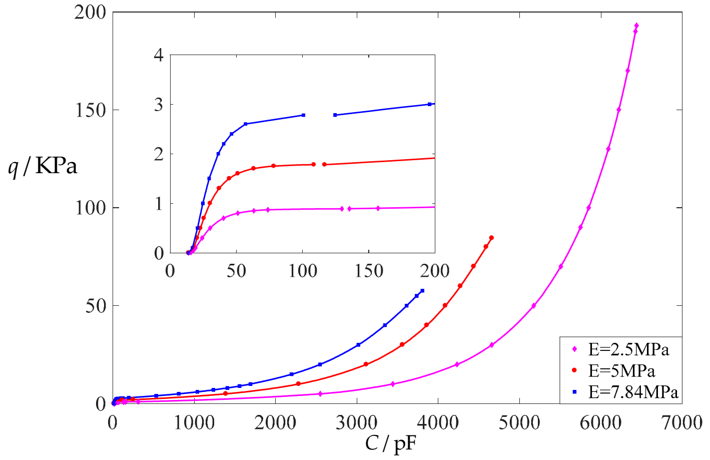

Figure S8. The capacitance-pressure relationships of a circular capacitive pressure sensor from non-touch mode of operation to touch mode of operation, where a = 100 mm, h = 1 mm, t = 0.1 mm, E = 7.84 MPa, 5 MPa and 2.5 MPa, ν = 0.47, and g = 20 mm.

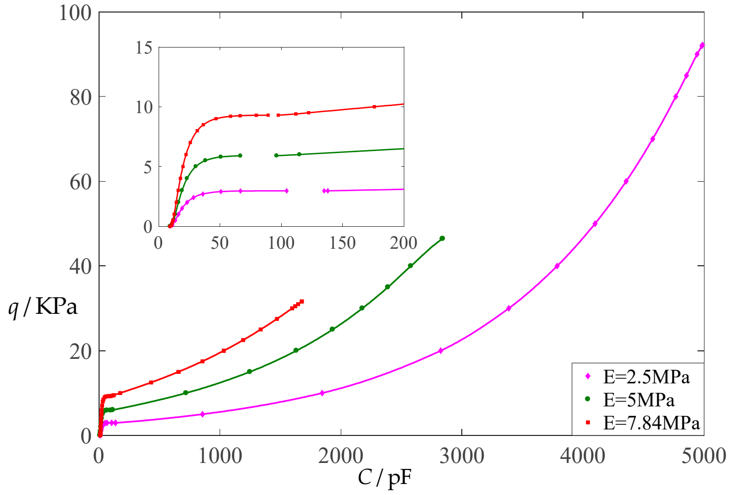

Figure S9. The capacitance-pressure relationships of a circular capacitive pressure sensor from non-touch mode of operation to touch mode of operation, where a = 100 mm, h = 1 mm, t = 0.1 mm, E = 7.84 MPa, 5 MPa and 2.5 MPa, ν = 0.47, and g = 30 mm.

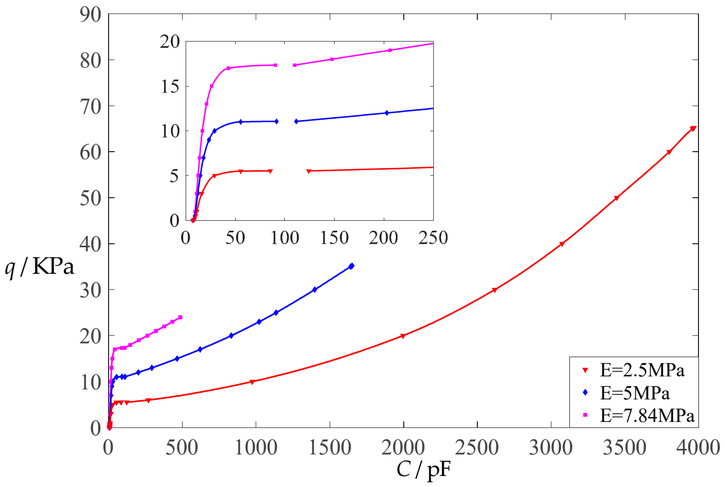

Figure S10. The capacitance-pressure relationships of a circular capacitive pressure sensor from non-touch mode of operation to touch mode of operation, where a = 100 mm, h = 1 mm, t = 0.1 mm, E = 7.84 MPa, 5 MPa and 2.5 MPa, ν = 0.47, and g = 37 mm.

S.6. Effect of Thickness of Insulator Layer on Capacitance–Pressure Relationships

In this section, the insulator layer thickness t is first increased from the reference value t = 0.1 mm to t = 0.15 mm, and then further to t = 0.3 mm. The initial air parallel gap g still takes 10 mm, 20 mm, 30 mm and 37 mm. The calculation results are listed in Tables S41 –S48 when t = 0.15 mm, and in Tables S49–S56 when t = 0.3 mm. Figure S11 shows the input capacitance–output pressure relationships for g = 10 mm and t = 0.1 mm, 0.15 mm and 0.3 mm, Figure S12 for g = 20 mm and t = 0.1 mm, 0.15 mm and 0.3 mm, Figure S13 for g = 30 mm and t = 0.1 mm, 0.15 mm and 0.3 mm, and Figure S14 for g = 37 mm and t = 0.1 mm, 0.15 mm and 0.3 mm.

From Figures S11–S14 it can clearly be seen that increasing the insulator layer thickness t can only increase the range of the input capacitance C, but the range of the output pressure q is almost unchanged. Therefore, increasing the insulator layer thickness t can decrease the output pressure per unit capacitance, because the range of the input capacitance C increases while the range of the output pressure q remains constant. For instance, as the Young's modulus of elasticity E decreases from 7.84 MPa to 5 MPa and then to 2.5 MPa, the output pressure per unit capacitance for g = 10 mm increases from 0.029 KPa/pF to 0.043 KPa/pF and then to 0.083 KPa/pF, that for g = 20 mm increases from 0.015 KPa/pF to 0.022 KPa/pF and then to 0.039 KPa/pF, that for g = 30 mm increases from 0.019 KPa/pF to 0.026 KPa/pF and then to 0.042 KPa/pF, and that for g = 37 mm increases from 0.050 KPa/pF to 0.063 KPa/pF and then to 0.89 KPa/pF, which are calculated from Tables S1–S8 and S41–S56.

Table S41. The calculation results of a circular capacitive pressure sensor operating in non-touch mode, where a = 100 mm, h = 1 mm, t = 0.15 mm, E = 7.84 MPa, ν = 0.47, and g = 10 mm.

q/KPa	w_m/mm	σ_m/MPa	C/pF
0	0.000	0.000	27.637
0.10	6.564	0.042	45.644
0.15	7.515	0.055	52.179
0.20	8.273	0.067	60.040
0.25	8.913	0.078	70.763
0.30	9.473	0.088	88.819

0.35	9.974	0.098	114.806
0.399	9.993	0.098	149.670

Table S42. The calculation results of a circular capacitive pressure sensor operating in touch mode, where a = 100 mm, h = 1 mm, t = 0.15 mm, E = 7.84 MPa, ν = 0.47, and g = 10 mm.

q/KPa	d/mm	b_0	b_1	c_0	σ_m/ MPa	C/pF
0.4	0.052	0.01351	−0.00210	0.07271	0.106	158.244
0.5	20.396	0.01395	−0.00248	0.07380	0.113	544.852
1	39.082	0.01885	−0.00261	0.07383	0.150	1161.675
5	65.422	0.03640	−0.00316	0.07536	0.287	2360.961
10	72.648	0.04896	−0.00355	0.07640	0.385	2786.742
20	78.224	0.06745	−0.00415	0.07779	0.530	3150.473
30	80.876	0.08254	−0.00480	0.07881	0.649	3335.573
40	82.538	0.09587	−0.00552	0.07965	0.753	3455.823
50	83.722	0.10804	−0.00631	0.08037	0.849	3543.552
60	84.628	0.11933	−0.00715	0.08102	0.938	3612.208
70	85.358	0.12992	−0.00803	0.08161	1.021	3668.413
80	85.966	0.13992	−0.00893	0.08215	1.100	3715.990
90	86.484	0.14942	−0.00985	0.08265	1.175	3757.340
100	86.936	0.15848	−0.01077	0.08313	1.246	3793.977
110	87.336	0.16715	−0.01171	0.08358	1.315	3827.040
120	87.694	0.17547	−0.01264	0.08401	1.380	3857.409
130	88.018	0.18347	−0.01358	0.08443	1.443	3885.821
140	88.312	0.19119	−0.01451	0.08482	1.504	3913.107
150	88.584	0.19864	−0.01544	0.08520	1.563	3940.322
160	88.834	0.20584	−0.01636	0.08557	1.619	3970.218
171	89.088	0.21351	−0.01737	0.08597	1.680	4000.602

Table S43. The calculation results of a circular capacitive pressure sensor operating in non-touch mode, where a = 100 mm, h = 1 mm, t = 0.15 mm, E = 7.84 MPa, ν = 0.47, and g = 20 mm.

q/KPa	w_m/mm	σ_m/MPa	C/pF
0	0.000	0.000	13.860
0.1	6.564	0.042	16.907
0.5	11.237	0.124	20.648
1.0	14.173	0.198	24.661
1.5	16.237	0.261	29.410
2.0	17.884	0.317	36.274
2.2	18.466	0.338	40.368
2.4	19.014	0.359	46.242
2.6	19.532	0.379	56.658
2.779	19.991	0.394	99.978

Table S44. The calculation results of a circular capacitive pressure sensor operating in touch mode, where a = 100 mm, h = 1 mm, t = 0.15 mm, E = 7.84 MPa, ν = 0.47, and g = 20 mm.

q/KPa	d/mm	b_0	b_1	c_0	σ_m/ MPa	C/pF

2.780	0.794	0.04857	−0.00817	0.15437	0.396	123.242
3	8.508	0.05026	−0.00876	0.15053	0.409	177.583
4	20.960	0.05758	−0.00927	0.14909	0.463	426.680
5	27.848	0.06400	−0.00936	0.14921	0.511	623.679
6	32.742	0.06971	−0.00938	0.14953	0.555	784.416
7	36.518	0.07490	−0.00938	0.14990	0.595	919.744
8	39.568	0.07970	−0.00938	0.15028	0.632	1035.936
9	42.106	0.08418	−0.00939	0.15065	0.666	1137.683
10	44.268	0.08841	−0.00940	0.15101	0.699	1227.901
15	51.726	0.10704	−0.00951	0.15264	0.844	1567.832
20	56.302	0.12307	−0.00968	0.15401	0.970	1800.088
30	61.918	0.15095	−0.01016	0.15627	1.189	2115.543
40	65.414	0.17540	−0.01084	0.15813	1.381	2336.543
50	67.900	0.19758	−0.01169	0.15974	1.556	2523.081
55	68.910	0.20799	−0.01216	0.16049	1.638	2610.383
57.65	69.400	0.21334	−0.01243	0.16087	1.680	2661.760

Table S45. The calculation results of a circular capacitive pressure sensor operating in non-touch mode, where a = 100 mm, h = 1 mm, t = 0.15 mm, E = 7.84 MPa, ν = 0.47, and g = 30 mm.

q/KPa	w_m/mm	σ_m/MPa	C/pF
0	0.000	0.000	9.249
0.1	6.564	0.042	10.481
0.3	9.473	0.088	11.189
0.5	11.237	0.124	11.688
1.0	14.173	0.198	12.674
2.0	17.884	0.317	14.337
3.0	20.496	0.419	15.966
4.0	22.579	0.511	17.736
5.0	24.342	0.596	19.798
6.0	25.884	0.677	22.365
7.0	27.264	0.755	25.868
8.0	28.519	0.829	31.497
8.5	29.108	0.865	36.397
9.0	29.674	0.901	46.582
9.2	29.895	0.916	58.400
9.25	29.949	0.919	66.151
9.29	29.993	0.922	79.202
9.293	29.999	0.922	88.755

Table S46. The calculation results of a circular capacitive pressure sensor operating in touch mode, where a = 100 mm, h = 1 mm, t = 0.15 mm, E = 7.84 MPa, ν = 0.47, and g = 30 mm.

q/KPa	d/mm	b_0	b_1	c_0	σ_m/ MPa	C/pF
9.294	1.854	0.11188	−0.01698	0.23288	0.924	96.072
9.4	4.626	0.11309	−0.01743	0.23056	0.930	106.258

9.5	5.596	0.11370	−0.01758	0.22996	0.933	114.281
10.0	9.186	0.11669	−0.01802	0.22839	0.955	155.277
12.5	19.112	0.13094	−0.01860	0.22717	1.051	343.857
15.0	25.142	0.14394	−0.01856	0.22766	1.149	506.224
17.5	29.592	0.15593	−0.01840	0.22842	1.241	647.418
20.0	33.114	0.16713	−0.01822	0.22925	1.327	771.892
22.5	36.014	0.17770	−0.01806	0.23010	1.408	883.683
25.0	38.466	0.18777	−0.01792	0.23093	1.486	985.235
27.5	40.580	0.19741	−0.01781	0.23174	1.561	1079.791
30.0	42.432	0.20669	−0.01772	0.23252	1.634	1168.749
30.5	42.776	0.20851	−0.01771	0.23267	1.648	1186.444
31.0	43.112	0.21031	−0.01770	0.23283	1.662	1204.059
31.6	43.504	0.21246	−0.01768	0.23301	1.679	1225.118
31.64	43.530	0.21261	−0.01768	0.23302	1.680	1226.406

Table S47. The calculation results of a circular capacitive pressure sensor operating in non-touch mode, where a = 100 mm, h = 1 mm, t = 0.15 mm, E = 7.84 MPa, ν = 0.47, and g = 37 mm.

q/KPa	w_m/mm	σ_m/MPa	C/pF
0	0.000	0.000	7.500
0.1	6.564	0.042	8.284
1	14.173	0.198	9.545
3	20.496	0.419	11.134
5	24.342	0.596	12.565
7	27.264	0.755	14.093
10	30.746	0.972	16.904
13	33.587	1.173	21.169
15	35.244	1.302	26.285
17	36.760	1.426	43.153
17.332	36.999	1.446	90.203

Table S48. The calculation results of a circular capacitive pressure sensor operating in touch mode, where a = 100 mm, h = 1 mm, t = 0.15 mm, E = 7.84 MPa, ν = 0.47, and g = 37 mm.

q/KPa	d/mm	b_0	b_1	c_0	σ_m/ MPa	C/pF
17.333	3.140	0.17574	-0.02449	0.28691	1.447	107.044
18	7.972	0.17938	-0.02496	0.28530	1.449	132.222
19	11.110	0.18478	-0.02533	0.28429	1.489	176.448
20	13.600	0.19007	-0.02551	0.28390	1.529	219.981
21	15.712	0.19526	-0.02559	0.28380	1.568	262.626
22	17.568	0.20034	-0.02561	0.28384	1.606	304.362
23	19.230	0.20532	-0.02559	0.28398	1.643	345.441
23.99	20.726	0.21015	-0.02554	0.28417	1.680	385.338

Table S49. The calculation results of a circular capacitive pressure sensor operating in non-touch mode, where a = 100 mm, h = 1 mm, t = 0.3 mm, E = 7.84 MPa, ν = 0.47, and g = 10 mm.

q/KPa	w_m/mm	σ_m/MPa	C/pF

0	0.000	0.000	27.473
0.10	6.564	0.042	45.199
0.15	7.515	0.055	51.598
0.20	8.273	0.067	59.272
0.25	8.913	0.078	69.699
0.30	9.473	0.088	87.149
0.35	9.974	0.098	112.032
0.399	9.993	0.098	147.158

Table S50. The calculation results of a circular capacitive pressure sensor operating in touch mode, where a = 100 mm, h = 1 mm, t = 0.3 mm, E = 7.84 MPa, ν = 0.47, and g = 10 mm.

q/KPa	d/mm	b_0	b_1	c_0	σ_m/ MPa	C/pF
0.4	0.052	0.01351	−0.00210	0.07271	0.106	153.020
0.5	20.396	0.01395	−0.00248	0.07380	0.113	422.586
1	39.082	0.01885	−0.00261	0.07383	0.150	760.671
5	65.422	0.03640	−0.00316	0.07536	0.287	1321.990
10	72.648	0.04896	−0.00355	0.07640	0.385	1517.669
20	78.224	0.06745	−0.00415	0.07779	0.530	1685.522
30	80.876	0.08254	−0.00480	0.07881	0.649	1771.182
40	82.538	0.09587	−0.00552	0.07965	0.753	1826.819
50	83.722	0.10804	−0.00631	0.08037	0.849	1867.348
60	84.628	0.11933	−0.00715	0.08102	0.938	1899.069
70	85.358	0.12992	−0.00803	0.08161	1.021	1924.993
80	85.966	0.13992	−0.00893	0.08215	1.100	1946.919
90	86.484	0.14942	−0.00985	0.08265	1.175	1965.993
100	86.936	0.15848	−0.01077	0.08313	1.246	1982.878
110	87.336	0.16715	−0.01171	0.08358	1.315	1998.127
120	87.694	0.17547	−0.01264	0.08401	1.380	2012.169
130	88.018	0.18347	−0.01358	0.08443	1.443	2025.359
140	88.312	0.19119	−0.01451	0.08482	1.504	2038.148
150	88.584	0.19864	−0.01544	0.08520	1.563	2051.047
160	88.834	0.20584	−0.01636	0.08557	1.619	2065.602
171	89.088	0.21351	−0.01737	0.08597	1.680	2080.158

Table S51. The calculation results of a circular capacitive pressure sensor operating in non-touch mode, where a = 100 mm, h = 1 mm, t = 0.3 mm, E = 7.84 MPa, ν = 0.47, and g = 20 mm.

q/KPa	w_m/mm	σ_m/MPa	C/pF
0	0.000	0.000	13.819
0.1	6.564	0.042	16.846
0.5	11.237	0.124	20.557
1.0	14.173	0.198	24.531
1.5	16.237	0.261	29.224
2.0	17.884	0.317	35.993
2.2	18.466	0.338	40.020

2.4	19.014	0.359	45.785
2.6	19.532	0.379	55.974
2.779	19.991	0.394	97.867

Table S52. The calculation results of a circular capacitive pressure sensor operating in touch mode, where a = 100 mm, h = 1 mm, t = 0.3 mm, E = 7.84 MPa, ν = 0.47, and g = 20 mm.

q/KPa	d/mm	b_0	b_1	c_0	σ_m/ MPa	C/pF
2.780	0.794	0.04857	−0.00817	0.15437	0.396	119.919
3	8.508	0.05026	−0.00876	0.15053	0.409	156.434
4	20.960	0.05758	−0.00927	0.14909	0.463	314.163
5	27.848	0.06400	−0.00936	0.14921	0.511	428.550
6	32.742	0.06971	−0.00938	0.14953	0.555	517.257
7	36.518	0.07490	−0.00938	0.14990	0.595	589.580
8	39.568	0.07970	−0.00938	0.15028	0.632	650.239
9	42.106	0.08418	−0.00939	0.15065	0.666	702.582
10	44.268	0.08841	−0.00940	0.15101	0.699	748.447
15	51.726	0.10704	−0.00951	0.15264	0.844	918.840
20	56.302	0.12307	−0.00968	0.15401	0.970	1034.068
30	61.918	0.15095	−0.01016	0.15627	1.189	1191.091
40	65.414	0.17540	−0.01084	0.15813	1.381	1303.259
50	67.900	0.19758	−0.01169	0.15974	1.556	1402.818
55	68.910	0.20799	−0.01216	0.16049	1.638	1450.855
57.65	69.400	0.21334	−0.01243	0.16087	1.680	1480.379

Table S53. The calculation results of a circular capacitive pressure sensor operating in non-touch mode, where a = 100 mm, h = 1 mm, t = 0.3 mm, E = 7.84 MPa, ν = 0.47, and g = 30 mm.

q/KPa	w_m/mm	σ_m/MPa	C/pF
0	0.000	0.000	9.230
0.1	6.564	0.042	10.457
0.3	9.473	0.088	11.162
0.5	11.237	0.124	11.659
1.0	14.173	0.198	12.640
2.0	17.884	0.317	14.293
3.0	20.496	0.419	15.911
4.0	22.579	0.511	17.669
5.0	24.342	0.596	19.714
6.0	25.884	0.677	22.258
7.0	27.264	0.755	25.724
8.0	28.519	0.829	31.284
8.5	29.108	0.865	36.114
9.0	29.674	0.901	46.119
9.2	29.895	0.916	57.673
9.25	29.949	0.919	65.220
9.29	29.993	0.922	77.872

9.293	29.999	0.922	87.088

Table S54. The calculation results of a circular capacitive pressure sensor operating in touch mode, where a = 100 mm, h = 1 mm, t = 0.3 mm, E = 7.84 MPa, ν = 0.47, and g = 30 mm.

q/KPa	d/mm	b_0	b_1	c_0	σ_m/ MPa	C/pF
9.294	1.854	0.11188	−0.01698	0.23288	0.924	93.387
9.4	4.626	0.11309	−0.01743	0.23056	0.930	99.332
9.5	5.596	0.11370	−0.01758	0.22996	0.933	104.914
10.0	9.186	0.11669	−0.01802	0.22839	0.955	132.854
12.5	19.112	0.13094	−0.01860	0.22717	1.051	252.626
15.0	25.142	0.14394	−0.01856	0.22766	1.149	349.726
17.5	29.592	0.15593	−0.01840	0.22842	1.241	431.398
20.0	33.114	0.16713	−0.01822	0.22925	1.327	501.901
22.5	36.014	0.17770	−0.01806	0.23010	1.408	564.567
25.0	38.466	0.18777	−0.01792	0.23093	1.486	621.181
27.5	40.580	0.19741	−0.01781	0.23174	1.561	674.188
30.0	42.432	0.20669	−0.01772	0.23252	1.634	724.418
30.5	42.776	0.20851	−0.01771	0.23267	1.648	734.588
31.0	43.112	0.21031	−0.01770	0.23283	1.662	744.752
31.6	43.504	0.21246	−0.01768	0.23301	1.679	756.974
31.64	43.530	0.21261	−0.01768	0.23302	1.680	757.691

Table S55. The calculation results of a circular capacitive pressure sensor operating in non-touch mode, where a = 100 mm, h = 1 mm, t = 0.3 mm, E = 7.84 MPa, ν = 0.47, and g = 37 mm.

q/KPa	w_m/mm	σ_m/MPa	C/pF
0	0.000	0.000	7.488
0.1	6.564	0.042	8.269
1	14.173	0.198	9.526
3	20.496	0.419	11.107
5	24.342	0.596	12.531
7	27.264	0.755	14.050
10	30.746	0.972	16.843
13	33.587	1.173	21.073
15	35.244	1.302	26.137
17	36.760	1.426	42.755
17.332	36.999	1.446	88.482

Table S56. The calculation results of a circular capacitive pressure sensor operating in touch mode, where a = 100 mm, h = 1 mm, t = 0.3 mm, E = 7.84 MPa, ν = 0.47, and g = 37 mm.

q/KPa	d/mm	b_0	b_1	c_0	σ_m/ MPa	C/pF
17.333	3.140	0.17574	-0.02449	0.28691	1.447	102.541
18	7.972	0.17938	-0.02496	0.28530	1.449	118.987
19	11.110	0.18478	-0.02533	0.28429	1.489	144.811
20	13.600	0.19007	-0.02551	0.28390	1.529	173.261
21	15.712	0.19526	-0.02559	0.28380	1.568	200.701

22	17.568	0.20034	-0.02561	0.28384	1.606	227.233
23	19.230	0.20532	-0.02559	0.28398	1.643	253.196
23.99	20.726	0.21015	-0.02554	0.28417	1.680	278.267

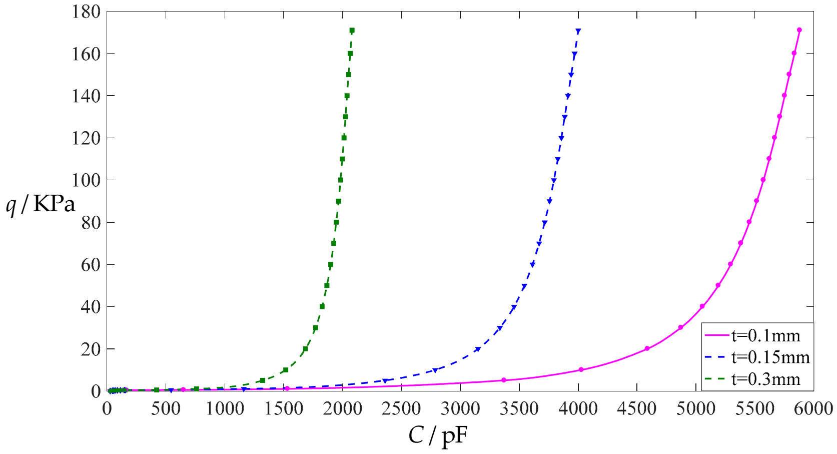

(a)

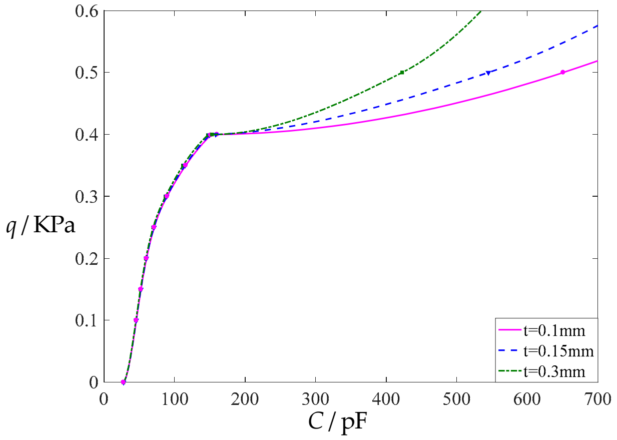

(b)

Figure S11. The capacitance-pressure relationships of a circular capacitive pressure sensor from non-touch mode of operation to touch mode of operation, where a = 100 mm, h = 1 mm, t = 0.1 mm, 0.15 mm and 0.3 mm, E = 7.84 MPa, ν = 0.47, and g = 10 mm, (a) Overall; (b) Local.

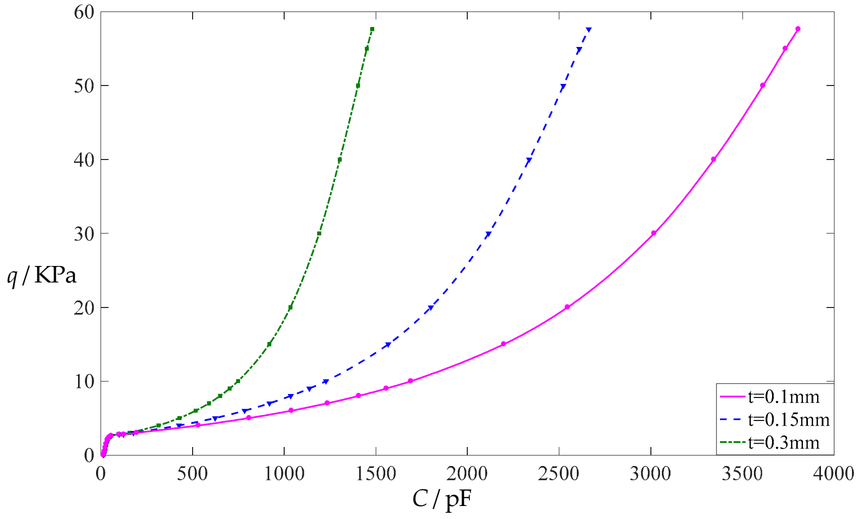

(a)

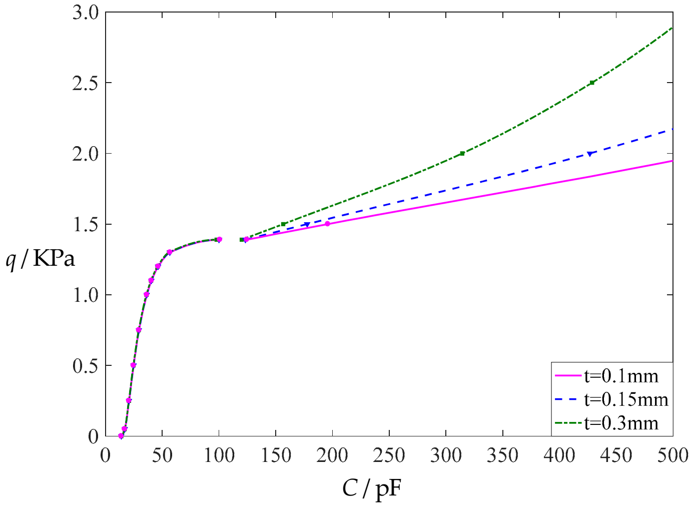

(b)

Figure S12. The capacitance-pressure relationships of a circular capacitive pressure sensor from non-touch mode of operation to touch mode of operation, where a = 100 mm, h = 1 mm, t = 0.1 mm, 0.15 mm and 0.3 mm, E = 7.84 MPa, ν = 0.47, and g = 20 mm, (a) Overall; (b) Local.

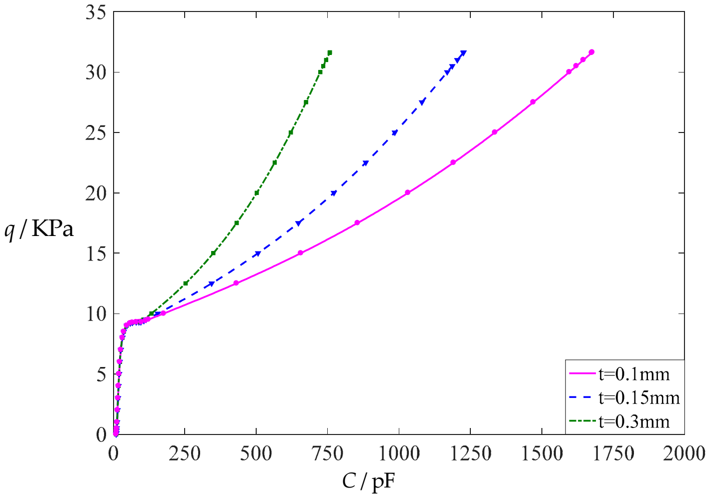

(a)

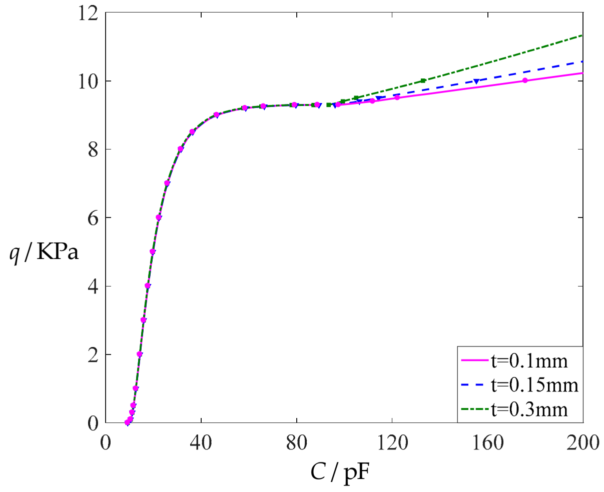

(b)

Figure S13. The capacitance-pressure relationships of a circular capacitive pressure sensor from non-touch mode of operation to touch mode of operation, where a = 100 mm, h = 1 mm, t = 0.1 mm, 0.15 mm and 0.3 mm, E = 7.84 MPa, ν = 0.47, and g = 30 mm, (a) Overall; (b) Local.

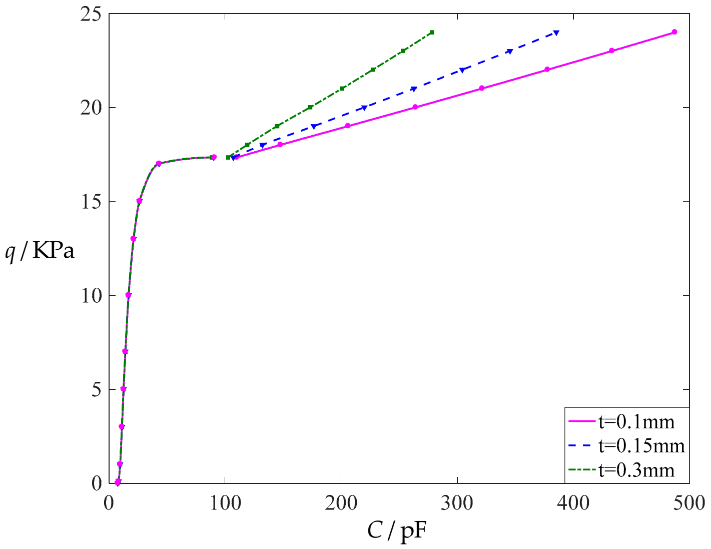

Figure S14. The capacitance-pressure relationships of a circular capacitive pressure sensor from non-touch mode of operation to touch mode of operation, where a = 100 mm, h = 1 mm, t = 0.1 mm, 0.15 mm and 0.3 mm, E = 7.84 MPa, ν = 0.47, and g = 37 mm.

S.7. Effect of Membrane Radius on Capacitance–Pressure Relationships

In this section, the radius a of the circular conductive membrane is first decreased from the reference value a = 100 mm to a = 50 mm, and then further to a = 10 mm. The initial air parallel gap g takes 5 mm, 10 mm, 15 mm and 18.5 mm for a = 50 mm, and takes 1 mm, 2 mm, 3 mm and 3.7 mm for a = 10 mm. The calculation results are listed in Tables S57 –S64 when a = 50 mm, and in Tables S65–S72 when a = 10 mm. Figure S15 shows the input capacitance–output pressure relationships for a = 100 mm and g = 10 mm, a = 50 mm and g = 5 mm, and a = 10 mm g = 1 mm, Figure S16 for a = 100 mm and g = 20 mm, a = 50 mm and g = 10 mm, and a = 10 mm g = 2 mm, Figure S17 for a = 100 mm and g = 30 mm, a = 50 mm and g = 15 mm, and a = 10 mm g = 3 mm, and Figure S18 for a = 100 mm and g = 37 mm, a = 50 mm and g = 18.5 mm, and a = 10 mm g = 3.7 mm.

from Figures S15–S18 it can clearly be seen that decreasing the membrane radius a can increase the range of the output pressure q but decreases the range of the input capacitance C. Therefore, decreasing the membrane radius a can greatly increase the output pressure per unit capacitance, because the range of the output pressure q increases since the thickness h of the circular conductive membrane is kept constant at 1 mm, while the range of the input capacitance C is greatly decreased since the area of the circular conductive membrane (movable electrode plate) is greatly reduced. For instance, as the membrane radius a decreases from 100 mm to 50 mm and then to 10 mm, the output pressure per unit capacitance increases from 0.029 KPa/pF to 0.228 KPa/pF and then to 21.385 KPa/pF for a = 100 mm and g = 10 mm, a = 50 mm and g = 5 mm, and a = 10 mm g = 1 mm, from 0.015 KPa/pF to 0.120 KPa/pF and then to 10.541 KPa/pF for a = 100 mm and g = 20 mm, a = 50 mm and g = 10 mm, and a = 10 mm g = 2 mm, from 0.019 KPa/pF to 0.146 KPa/pF and then to 11.029 KPa/pF for a = 100 mm and g = 30 mm, a = 50 mm and g = 15 mm, and a = 10 mm g = 3 mm, and from 0.050 KPa/pF to 0.345 KPa/pF and then to 18.647 KPa/pF for a = 100 mm and g = 37 mm, a = 50 mm and g = 18.5 mm, and a = 10 mm g = 3.7 mm, which are calculated from Tables S1–S8 and S57–S72.

Table S57. The calculation results of a circular capacitive pressure sensor operating in non-touch mode, where a = 50 mm, h = 1 mm, t = 0.1 mm, E = 7.84 MPa, ν = 0.47, and g = 5 mm.

q/KPa	w_m/mm	σ_m/MPa	C/pF

0	0.000	0.000	13.764
0.2	3.282	0.042	22.673
0.3	3.757	0.055	25.895
0.4	4.136	0.067	29.763
0.5	4.456	0.078	35.025
0.6	4.736	0.088	43.849
0.7	4.987	0.098	65.741
0.798	4.999	0.098	115.257

Table S58. The calculation results of a circular capacitive pressure sensor operating in touch mode, where a = 50 mm, h = 1 mm, t = 0.1 mm, E = 7.84 MPa, ν = 0.47, and g = 5 mm.

q/KPa	d/mm	b_0	b_1	c_0	σ_m/ MPa	C/pF
0.8	0.026	0.01351	-0.00210	0.07271	0.106	148.420
1	10.198	0.01395	-0.00248	0.07380	0.113	203.070
2	19.541	0.01885	-0.00261	0.07383	0.150	421.546
10	32.711	0.03640	-0.00316	0.07536	0.287	889.915
40	39.112	0.06745	-0.00415	0.07779	0.530	1200.640
100	41.861	0.10804	-0.00631	0.08037	0.849	1353.508
140	42.679	0.12992	-0.00803	0.08161	1.021	1401.391
200	43.468	0.15848	-0.01077	0.08313	1.246	1448.600
240	43.847	0.17547	-0.01264	0.08401	1.380	1471.633
300	44.292	0.19864	-0.01544	0.08520	1.563	1498.971
320	44.417	0.20584	-0.01636	0.08557	1.619	1506.706
342	44.544	0.21351	-0.01737	0.08597	1.680	1514.594

Table S59. The calculation results of a circular capacitive pressure sensor operating in non-touch mode, where a = 50 mm, h = 1 mm, t = 0.1 mm, E = 7.84 MPa, ν = 0.47, and g = 10 mm.

q/KPa	w_m/mm	σ_m/MPa	C/pF
0	0.000	0.000	6.916
0.2	3.282	0.042	8.433
1	5.619	0.124	10.294
2	7.086	0.198	12.287
3	8.119	0.261	14.643
4	8.942	0.317	18.043
4.4	9.233	0.338	20.068
4.8	9.507	0.359	22.968
5.2	9.766	0.379	28.100
5.558	9.987	0.397	49.280

Table S60. The calculation results of a circular capacitive pressure sensor operating in touch mode, where a = 50 mm, h = 1 mm, t = 0.1 mm, E = 7.84 MPa, ν = 0.47, and g = 10 mm.

q/KPa	d/mm	b_0	b_1	c_0	σ_m/ MPa	C/pF
5.56	0.397	0.04857	-0.00817	0.15437	0.396	52.122
6	4.254	0.05026	-0.00876	0.15053	0.409	73.193
8	10.480	0.05758	-0.00927	0.14909	0.463	162.345

10	13.924	0.06400	-0.00936	0.14921	0.512	233.008
14	18.259	0.07490	-0.00938	0.14990	0.595	341.681
20	22.134	0.08841	-0.00940	0.15101	0.699	457.463
30	25.863	0.10704	-0.00951	0.15264	0.845	586.746
40	28.151	0.12307	-0.00968	0.15401	0.970	675.018
60	30.959	0.15095	-0.01016	0.15627	1.189	793.120
80	32.707	0.17540	-0.01084	0.15813	1.381	872.319
100	33.950	0.19758	-0.01169	0.15974	1.556	931.351
110	34.455	0.20799	-0.01216	0.16049	1.638	956.100
115.3	34.700	0.21334	-0.01243	0.16087	1.680	968.224

Table S61. The calculation results of a circular capacitive pressure sensor operating in non-touch mode, where a = 50 mm, h = 1 mm, t = 0.1 mm, E = 7.84 MPa, v = 0.47, and g = 15 mm.

q/KPa	w_m/mm	σ_m/MPa	C/pF
0	0.000	0.000	4.618
0.6	4.736	0.088	5.586
1	5.619	0.124	5.834
2	7.086	0.198	6.326
4	8.942	0.317	7.154
6	10.248	0.419	7.965
8	11.290	0.511	8.846
10	12.171	0.596	9.871
12	12.942	0.677	11.146
14	13.632	0.755	12.886
16	14.260	0.829	15.677
18	14.837	0.901	23.136
18.5	14.975	0.919	32.764
18.586	14.998	0.922	39.234

Table S62. The calculation results of a circular capacitive pressure sensor operating in touch mode, where a = 50 mm, h = 1 mm, t = 0.1 mm, E = 7.84 MPa, v = 0.47, and g = 15 mm.

q/KPa	d/mm	b_0	b_1	c_0	σ_m/ MPa	C/pF
18.6	1.729	0.11249	-0.01725	0.23142	0.924	42.034
20	4.593	0.11669	-0.01802	0.22839	0.955	63.639
25	9.556	0.13094	-0.01860	0.22717	1.051	131.784
30	12.571	0.14394	-0.01856	0.22766	1.149	189.585
35	14.796	0.15593	-0.01840	0.22842	1.241	239.552
40	16.557	0.16713	-0.01822	0.22925	1.327	283.497
45	18.007	0.17770	-0.01806	0.23010	1.408	322.775
50	19.233	0.18777	-0.01792	0.23093	1.486	357.986
55	20.290	0.19741	-0.01781	0.23174	1.561	389.990
60	21.216	0.20669	-0.01772	0.23252	1.634	419.177
63.28	21.765	0.21261	-0.01768	0.23302	1.680	437.042

Table S63. The calculation results of a circular capacitive pressure sensor operating in non-touch mode, where a = 50 mm, h = 1 mm, t = 0.1 mm, E = 7.84 MPa, ν = 0.47, and g = 18.5 mm.

q/KPa	w_m/mm	σ_m/MPa	C/pF
0	0.000	0.000	3.747
0.2	3.282	0.042	4.138
2	7.086	0.198	4.768
6	10.248	0.419	5.560
10	12.171	0.596	6.274
14	13.632	0.755	7.035
20	15.373	0.972	8.437
26	16.793	1.173	10.560
30	17.622	1.302	13.106
34	18.380	1.426	21.476
34.664	18.499	1.446	39.016

Table S64. The calculation results of a circular capacitive pressure sensor operating in touch mode, where a = 50 mm, h = 1 mm, t = 0.1 mm, E = 7.84 MPa, ν = 0.47, and g = 18.5 mm.

q/KPa	d/mm	b_0	b_1	c_0	σ_m/ MPa	C/pF
34.666	2.570	0.17574	-0.02449	0.28691	1.447	42.612
36	3.986	0.17938	-0.02496	0.28530	1.449	53.851
38	5.555	0.18478	-0.02533	0.28429	1.489	70.070
40	6.800	0.19007	-0.02551	0.28390	1.529	85.636
42	7.856	0.19526	-0.02559	0.28380	1.568	100.692
44	8.784	0.20034	-0.02561	0.28384	1.606	115.156
46	9.615	0.20532	-0.02559	0.28398	1.643	129.215
47.98	10.363	0.21015	-0.02554	0.28417	1.680	142.658

Table S65. The calculation results of a circular capacitive pressure sensor operating in non-touch mode, where a = 10 mm, h = 1 mm, t = 0.1 mm, E = 7.84 MPa, ν = 0.47, and g = 1 mm.

q/KPa	w_m/mm	σ_m/MPa	C/pF
0	0.000	0.000	2.648
1	0.656	0.042	4.257
1.5	0.751	0.055	4.820
2	0.827	0.067	5.483
2.5	0.891	0.078	6.364
3	0.947	0.088	7.787
3.5	0.997	0.098	10.496
3.99	0.999	0.098	15.656

Table S66. The calculation results of a circular capacitive pressure sensor operating in touch mode, where a = 10 mm, h = 1 mm, t = 0.1 mm, E = 7.84 MPa, ν = 0.47, and g = 1 mm.

q/KPa	d/mm	b_0	b_1	c_0	σ_m/ MPa	C/pF
4	0.005	0.01351	-0.00210	0.07271	0.106	16.530
5	2.040	0.01395	-0.00248	0.07380	0.113	17.687
10	3.908	0.01885	-0.00261	0.07383	0.150	28.626
50	6.542	0.03640	-0.00316	0.07536	0.287	51.959

200	7.822	0.06745	-0.00415	0.07779	0.530	67.195
500	8.372	0.10804	-0.00631	0.08037	0.849	74.662
700	8.536	0.12992	-0.00803	0.08161	1.021	77.014
1000	8.694	0.15848	-0.01077	0.08313	1.246	79.345
1200	8.769	0.17547	-0.01264	0.08401	1.380	80.485
1500	8.858	0.19864	-0.01544	0.08520	1.563	81.838
1600	8.883	0.20584	-0.01636	0.08557	1.619	82.221
1710	8.909	0.21351	-0.01737	0.08597	1.680	82.610

Table S67. The calculation results of a circular capacitive pressure sensor operating in non-touch mode, where a = 10 mm, h = 1 mm, t = 0.1 mm, E = 7.84 MPa, ν = 0.47, and g = 2 mm.

q/KPa	w_m/mm	σ_m/MPa	C/pF
0	0.000	0.000	1.356
1	0.656	0.042	1.647
5	1.124	0.124	2.000
10	1.417	0.198	2.374
15	1.624	0.261	2.810
20	1.788	0.317	3.431
22	1.847	0.338	3.794
24	1.901	0.359	4.309
26	1.953	0.379	5.200
27.79	1.997	0.397	7.032

Table S68. The calculation results of a circular capacitive pressure sensor operating in touch mode, where a = 10 mm, h = 1 mm, t = 0.1 mm, E = 7.84 MPa, ν = 0.47, and g = 2 mm.

q/KPa	d/mm	b_0	b_1	c_0	σ_m/ MPa	C/pF
27.8	0.079	0.04857	-0.00817	0.15437	0.396	7.522
30	0.851	0.05026	-0.00876	0.15053	0.409	9.625
40	2.096	0.05758	-0.00927	0.14909	0.463	15.078
50	2.785	0.06400	-0.00936	0.14921	0.512	18.974
70	3.652	0.07490	-0.00938	0.14990	0.595	24.755
100	4.427	0.08841	-0.00940	0.15101	0.699	30.768
150	5.173	0.10704	-0.00951	0.15264	0.845	37.329
200	5.630	0.12307	-0.00968	0.15401	0.970	41.740
300	6.192	0.15095	-0.01016	0.15627	1.189	47.565
400	6.541	0.17540	-0.01084	0.15813	1.381	51.423
500	6.790	0.19758	-0.01169	0.15974	1.556	54.277
550	6.891	0.20799	-0.01216	0.16049	1.638	55.463
576.5	6.940	0.21334	-0.01243	0.16087	1.680	56.044

Table S69. The calculation results of a circular capacitive pressure sensor operating in non-touch mode, where a = 10 mm, h = 1 mm, t = 0.1 mm, E = 7.84 MPa, ν = 0.47, and g = 3 mm.

q/KPa	w_m/mm	σ_m/MPa	C/pF
0	0.000	0.000	0.912
3	0.947	0.088	1.099

5	1.124	0.124	1.148
10	1.243	0.198	1.243
20	1.788	0.317	1.402
30	2.050	0.419	1.557
40	2.258	0.511	1.725
50	2.434	0.596	1.920
60	2.588	0.677	2.160
70	2.726	0.755	2.485
80	2.852	0.829	3.000
90	2.967	0.901	4.338
92.5	2.995	0.919	5.988
92.93	2.999	0.922	6.161

Table S70. The calculation results of a circular capacitive pressure sensor operating in touch mode, where a = 10 mm, h = 1 mm, t = 0.1 mm, E = 7.84 MPa, ν = 0.47, and g = 3 mm.

q/KPa	d/mm	b_0	b_1	c_0	σ_m/ MPa	C/pF
93	0.346	0.11249	-0.01725	0.23142	0.924	6.838
100	0.919	0.11669	-0.01802	0.22839	0.955	8.706
125	1.911	0.13094	-0.01860	0.22717	1.051	13.086
150	2.514	0.14394	-0.01856	0.22766	1.149	16.453
175	2.959	0.15593	-0.01840	0.22842	1.241	19.221
200	3.311	0.16713	-0.01822	0.22925	1.327	21.598
225	3.601	0.17770	-0.01806	0.23010	1.408	23.679
250	3.847	0.18777	-0.01792	0.23093	1.486	25.527
275	4.058	0.19741	-0.01781	0.23174	1.561	27.186
300	4.243	0.20669	-0.01772	0.23252	1.634	28.688
316.4	4.353	0.21261	-0.01768	0.23302	1.680	29.601

Table S71. The calculation results of a circular capacitive pressure sensor operating in non-touch mode, where a = 10 mm, h = 1 mm, t = 0.1 mm, E = 7.84 MPa, ν = 0.47, and g = 3.7 mm.

q/KPa	w_m/mm	σ_m/MPa	C/pF
0	0.000	0.000	0.741
1	0.656	0.042	0.818
10	1.417	0.198	0.941
30	2.050	0.419	1.095
50	2.434	0.596	1.232
70	2.726	0.755	1.379
100	3.075	0.972	1.647
130	3.359	1.173	2.050
150	3.524	1.302	2.526
170	3.676	1.426	4.045
173.32	3.699	1.446	5.992

Table S72. The calculation results of a circular capacitive pressure sensor operating in touch mode, where a = 10 mm, h = 1 mm, t = 0.1 mm, E = 7.84 MPa, ν = 0.47, and g = 3.7 mm.

q/KPa	d/mm	b_0	b_1	c_0	σ_m/ MPa	C/pF
173.33	0.514	0.17574	-0.02449	0.28691	1.447	6.874
180	0.797	0.17938	-0.02496	0.28530	1.449	7.830
190	1.111	0.18478	-0.02533	0.28429	1.489	9.038
200	1.360	0.19007	-0.02551	0.28390	1.529	10.098
210	1.571	0.19526	-0.02559	0.28380	1.568	11.066
220	1.757	0.20034	-0.02561	0.28384	1.606	11.967
230	1.923	0.20532	-0.02559	0.28398	1.643	12.813
239.9	2.073	0.21015	-0.02554	0.28417	1.680	13.606

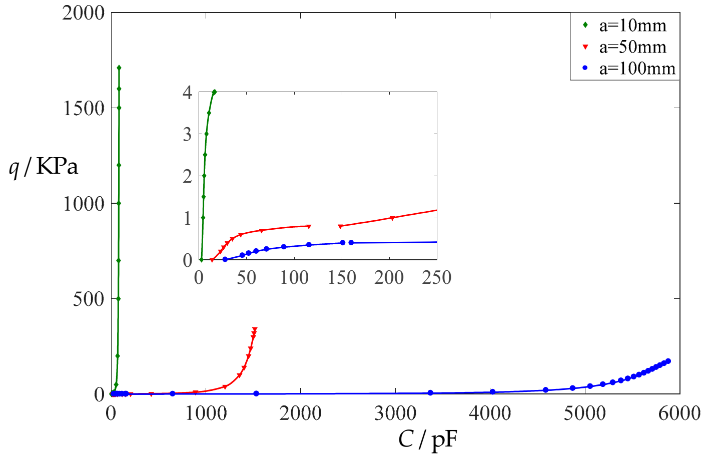

Figure S15. The capacitance-pressure relationships of a circular capacitive pressure sensor from non-touch mode of operation to touch mode of operation, where a = 100 mm, 50 mm and 10 mm, h = 1 mm, t = 0.1 mm, E = 7.84 MPa, ν = 0.47, and g = 10 mm for a = 100 mm, g = 5 mm for a = 50 mm, and g = 1 mm for a = 10 mm.

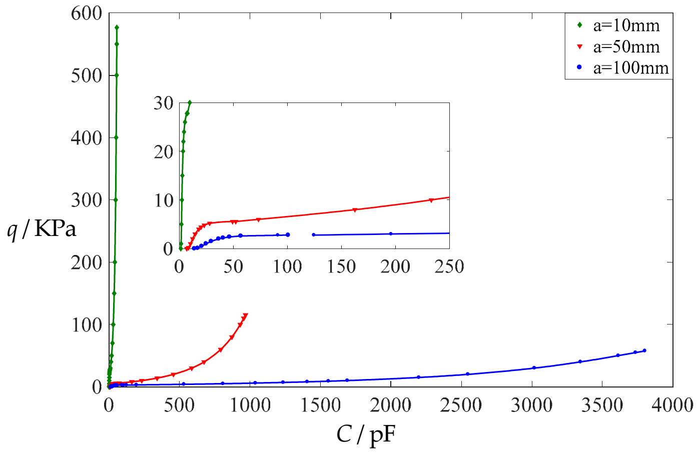

Figure S16. The capacitance-pressure relationships of a circular capacitive pressure sensor from non-touch mode of operation to touch mode of operation, where a = 100 mm, 50 mm and 10 mm, h = 1 mm, t = 0.1 mm, E = 7.84 MPa, ν = 0.47, and g = 20 mm for a = 100 mm, g = 10 mm for a = 50 mm, and g = 2 mm for a = 10 mm.

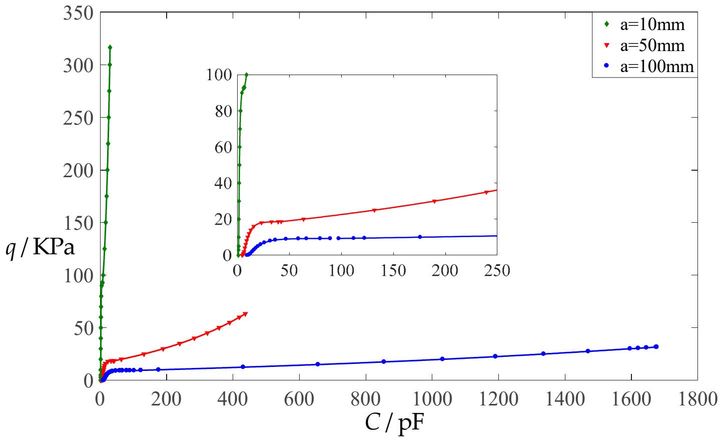

Figure S17. The capacitance-pressure relationships of a circular capacitive pressure sensor from non-touch mode of operation to touch mode of operation, where a = 100 mm, 50 mm and 10 mm, h = 1 mm, t = 0.1 mm, E = 7.84 MPa, ν = 0.47, and g = 30 mm for a = 100 mm, g = 15 mm for a = 50 mm, and g = 3 mm for a = 10 mm.

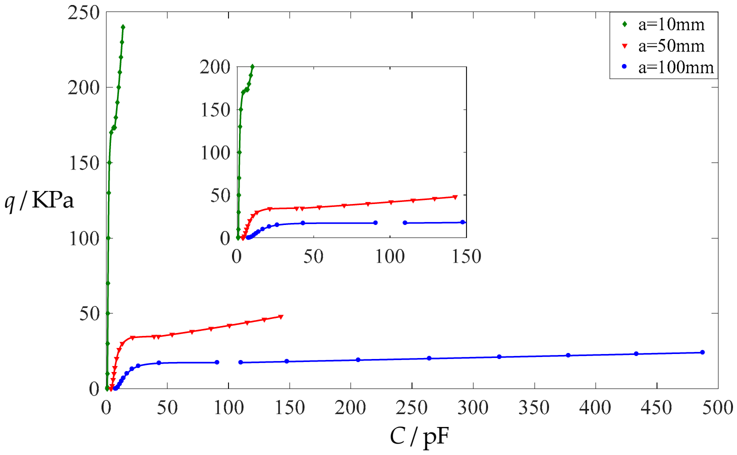

Figure S18. The capacitance-pressure relationships of a circular capacitive pressure sensor from non-touch mode of operation to touch mode of operation, where a = 100 mm, 50 mm and 10 mm, h = 1 mm, t = 0.1 mm, E = 7.84 MPa, ν = 0.47, and g = 37 mm for a = 100 mm, g = 18.5 mm for a = 50 mm, and g = 3.7 mm for a = 10 mm.
